# Thermodynamic Assessment of Sacrificial Oxidant Potential, H_2_O/O_2_ Potential, and Rate–Overpotential Relationship to Examine Catalytic Water Oxidation in Nonaqueous Solvents

**DOI:** 10.1021/acs.inorgchem.4c03897

**Published:** 2024-11-11

**Authors:** Shun-Chien Hsiao, Ting-Yi Chuang, Sharad V. Kumbhar, Tzuhsiung Yang, Yu-Heng Wang

**Affiliations:** Department of Chemistry, National Tsing Hua University, Hsinchu 30013, Taiwan

## Abstract

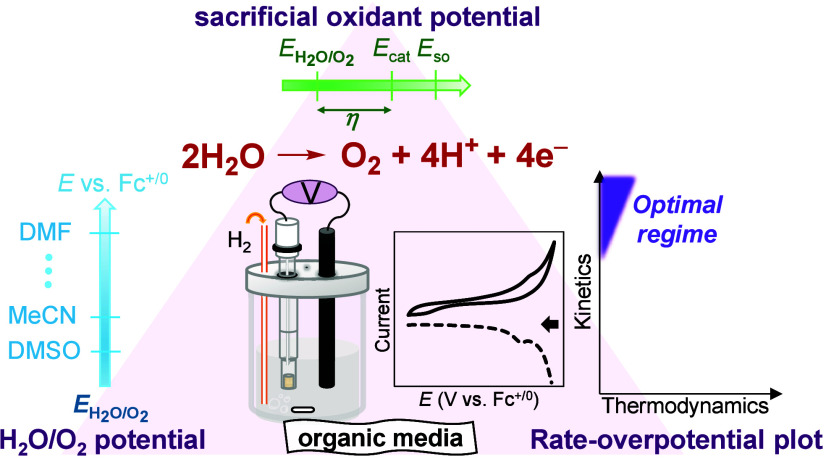

The water oxidation reaction (WOR), which is pivotal to storing energy in chemical bonds, requires a catalyst to overcome its inherent kinetic barrier. In bulk solutions, sacrificial oxidants (SOs) can regenerate the catalysts to ensure that the homogeneous WOR can be operated with long-term consistent performance. To implement this strategy for organic WOR systems, we modified four common SOs with tetra-*n*-butylammonium ([NBu_4_]^+^)—[NBu_4_]_2_[Ce(NO_3_)_6_], [NBu_4_][IO_4_], [NBu_4_][HSO_5_], and [NBu_4_]_2_[S_2_O_8_]—and examined their chemical stability and electrochemical behaviors in various organic solvents. We also derived the organic-solvent-associated redox potential of H_2_O/O_2_ in organic media (*E*_H_2_O/O_2_(org)_) using open-circuit potential measurements of the H^+^/H_2_ redox couple and the related thermochemical cycle. Our findings indicate that the *E*_H_2_O/O_2_(org)_ varies with solvent identity and can be adjusted by changing the [H_2_O], [acid], and [base] levels; thus, the SO should be carefully selected for WOR, because the innate redox potentials of SOs are not always higher than *E*_H_2_O/O_2_(org)_ under the studied conditions. Lastly, we obtained catalyst-performance-related insights via a rate–overpotential free-energy relationship by calculating the overpotentials of previously studied WOR systems in organic media.

## Abstract 2

We investigated catalytic water oxidation in nonaqueous solvents using modified sacrificial oxidants (SOs). The stability, redox behavior, and overpotentials of four common SOs in various organic solvents were assessed, and the results clarify the impact of the solvent on SO potentials. The rate-overpotential relationship of water oxidation was established on the basis of these findings, providing valuable insights into the catalyst performance of the molecular catalysts studied in organic media.

## Introduction

**Figure 1:**
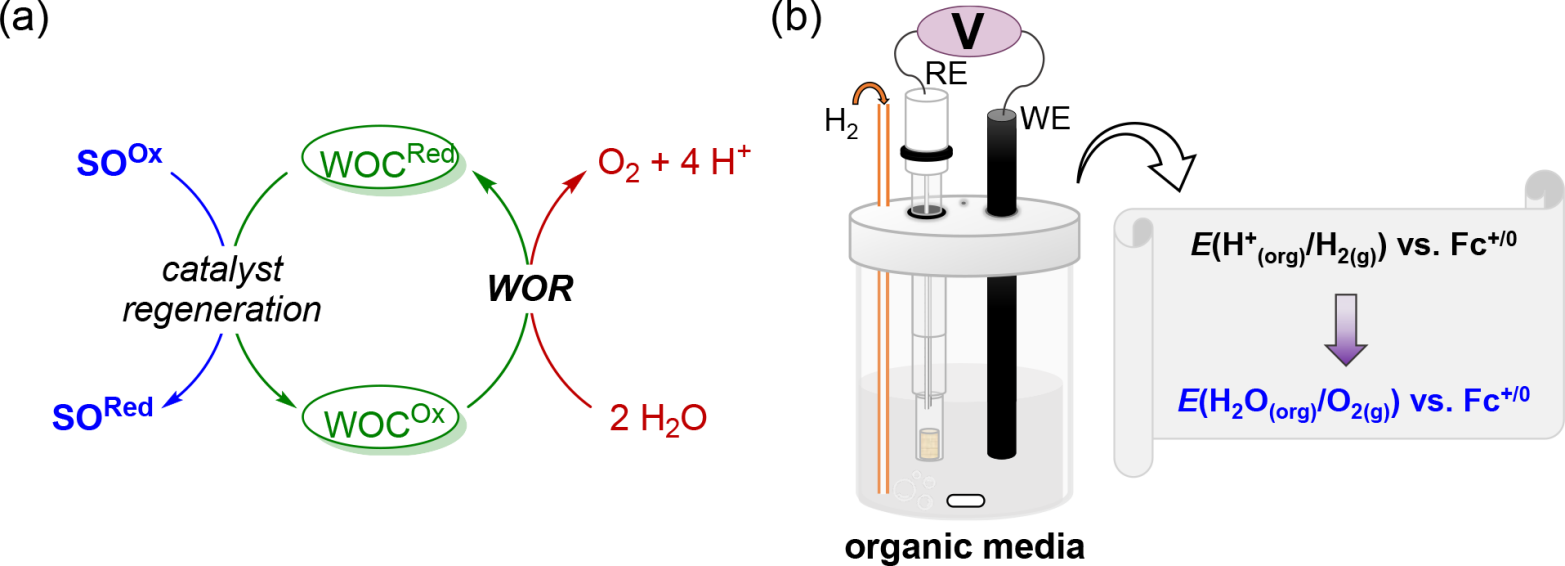
(a) Illustration describing the use of SOs to regenerate active catalysts, which is a common strategy in studies on the catalytic WOR (Ox, oxidized form; Red, reduced form). (b) Schematic of OCP measurements for determining *E*_H_2_O/O_2_(org)_ in organic media. The OCP is the potential at which no current flows between the working electrode (WE) and reference electrode (RE).

Mitigating the greenhouse effect and achieving carbon neutrality by 2050 can help alleviate climate change; specifically, the use of renewable energy sources instead of fossil fuels can limit carbon emissions.^1,2^ The water-splitting reaction is a noteworthy energy-conversion scheme for producing green H_2_ fuel.^3−5^ In artificial systems, the water oxidation reaction (WOR) is considered an anodic water-splitting reaction.^6,7^ The transfer of four electrons and protons and the evolution of an O_2_ molecule via O–O bond formation occur in a single turnover of the WOR (eq 1).^8,9^ However, catalysts are needed to overcome the kinetic barrier and thereby increase the WOR efficiency;^10−12^ thus, the performance and regeneration of WOR catalysts have attracted considerable interest. One common strategy for homogeneous WOR systems is to use sacrificial oxidants (SOs), such as cerium(IV) ammonium nitrate (CAN; [NH_4_]_2_[Ce(NO_3_)_6_]), sodium periodate (NaIO_4_), potassium peroxymonosulfate (KHSO_5_), and sodium peroxydisulfate (Na_2_S_2_O_8_), to regenerate the active catalyst for catalyzing the WOR (Figure 1a).^13^
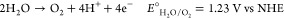
1

Compared to homogeneous electrochemical and photochemical WOR systems, the SO-assisted WOR (that is, the chemical WOR) offers several benefits, including cost-effectiveness of the electron source, operational simplicity, and rapid screening to optimize the reaction conditions and catalyst design.^14^ Although the four above-mentioned SOs have been comprehensively studied in terms of their oxidation potentials (*E*_so_) in aqueous solutions and Pourbaix diagrams, chemical and photochemical WOR systems have rarely been analyzed in nonaqueous solvents, thereby hindering the applicability of molecular-based water oxidation catalysts (MWOCs) in homogeneous catalysis.^14^ Nevertheless, upon replacing the alkali or ammonium cations with tetra-*n*-butylammonium ([NBu_4_]^+^), the four SOs can dissolve in common organic solvents, facilitating the investigation of their chemical stability and redox behavior in nonaqueous solvents, as achieved in the present study.

In addition to SOs, understanding the thermodynamic redox potential of water in organic media (*E*_H_2_O/O_2_(org)_) is a prerequisite for analyzing the overpotential (η)^15,16^ of MWOCs in water/organic solvent mixtures. Since the late 2000s, MWOCs based on first-row transition metals have attracted widespread attention for the WOR owing to their low cost and abundance.^17^ However, the η values of first-row-transition-metal-based MWOCs have generally not been reported in organic solvents, owing to an insufficient understanding of *E*_H_2_O/O_2_(org)_.^15,16^ In nonaqueous solvents, *E*_H_2_O/O_2_(org)_ is suggested to be derived from the redox potential of the corresponding H^+^/H_2_ redox couple (*E*_H^+^/H_2__), which can be determined through open-circuit potential (OCP; or equilibrium potential) measurements (Figure 1b).^18,19^ During OCP measurements, the electroactive components of the solution reach an equilibrium at the working electrode, with the resultant potential measured against a reference electrode, effectively turning the potentiostat into a voltmeter.

The measured OCP is derived from a combination of solvation energy, electrostatic interactions, and other solution-related factors. It is, therefore, a solely thermodynamic parameter.^20,21^ This approach has been validated in studies on *E*_H^+^/H_2__ in acetonitrile (MeCN) and dimethylformamide (DMF) and further extended to estimate the thermodynamic redox potentials of O_2_/H_2_O, NH_3_/N_2_, CO_2_/CO, and CO_2_/CH_4_ under nonstandard-state conditions.^19,22−25^ Therefore, in the present study, the thermodynamic potentials of H_2_O/O_2_ in different organic solvents were estimated using OCP measurements of *E*_H^+^/H_2__ and the related thermochemical cycle. This approach permits a comparison between *E*_so_ and *E*_H_2_O/O_2_(org)_ to ascertain the capacity for executing the catalytic WOR in nonaqueous solvents using a given SO. In addition, a rate–overpotential free-energy relationship was established by calculating the overpotentials of previously studied organic WOR systems, which facilitates benchmarking of the catalyst performance of MWOCs. The crucial thermodynamic insights into oxidative reactions in organic media can be extended to fields such as energy conversion and organic transformation.

## Results and Discussion

### Synthesis, Stability, and Electrochemical Properties of Sacrificial Oxidants

**Scheme 1:**
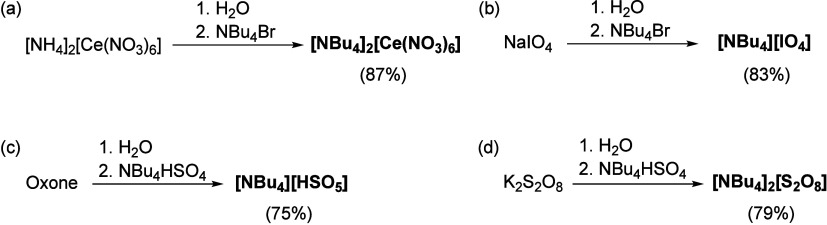
Synthesis Procedures for the Four Modified SOs: (a) [NBu_4_]_2_[Ce(NO_3_)_6_], (b) [NBu_4_][IO_4_], (c) [NBu_4_][HSO_5_], and (d) [NBu_4_]_2_[S_2_O_8_] The percentages in parentheses represent the yield; see Section S2 for details.

**Figure 2:**
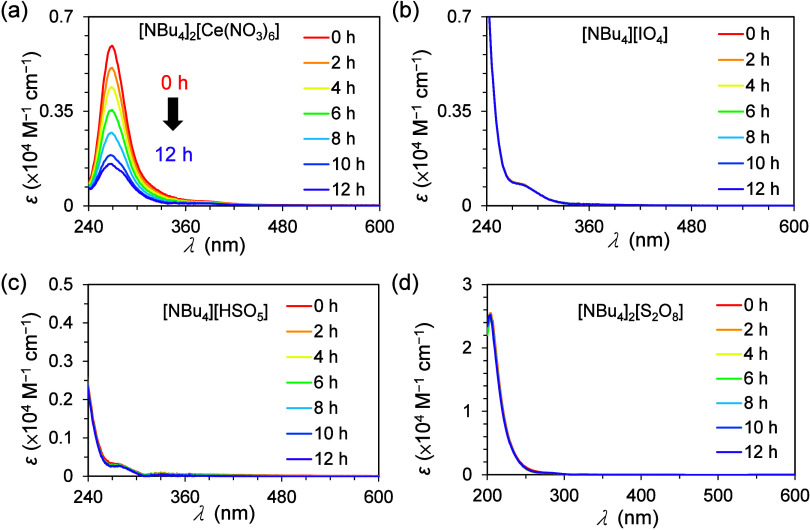
UV–vis spectra of the (a) [NBu_4_]_2_[Ce(NO_3_)_6_], (b) [NBu_4_][IO_4_], (c) [NBu_4_][HSO_5_], and (d) [NBu_4_]_2_[S_2_O_8_] SOs in MeCN. See Section S3 for the UV–vis spectra of these SOs in the selected 12 organic solvents.

The above-mentioned SOs ([NH_4_]_2_[Ce(NO_3_)_6_], NaIO_4_, KHSO_5_, and Na_2_S_2_O_8_) have been extensively employed to investigate the homogeneous chemical WOR under aqueous conditions;^13,17^ unfortunately, their chemical properties and redox potentials in organic solvents have not been thoroughly assessed. To fill this knowledge gap and facilitate their application in water oxidation and redox chemistry in mixtures of water and organic solvents, the four SOs were altered using [NBu_4_]^+^ as the countercation (Scheme 1 and Section S2).^26−29^ This modification allowed the four resulting SOs—[NBu_4_]_2_[Ce(NO_3_)_6_], [NBu_4_][IO_4_], [NBu_4_][HSO_5_], and [NBu_4_]_2_[S_2_O_8_]—to be soluble in various organic solvents, laying the foundation for the present study. The stability of the four modified SOs in 12 organic solvents was evaluated by ultraviolet–visible (UV–vis) spectroscopy over 12 h. The organic solvents were dichloromethane (DCM), isopropanol (IPA), tetrahydrofuran (THF), ethyl acetate (EA), dioxane, acetone, methanol (MeOH), ethanol (EtOH), MeCN, DMF, *N,N*-dimethylacetamide (DMA), and dimethyl sulfoxide (DMSO). The UV–vis spectra of [NBu_4_]_2_[Ce(NO_3_)_6_] in MeCN revealed significant decomposition (Figure 2a and Table 1); this observation is consistent with the reported stability of CAN under acidic conditions (pH < 3).^30^ In contrast, [NBu_4_][IO_4_] exhibited good chemical stability in various organic solvents, as suggested by the near-identical UV–vis spectra acquired over 12 h (Figure 2b and Table 1). Furthermore, the stability of [NBu_4_][HSO_5_] and [NBu_4_]_2_[S_2_O_8_] varied with the solvent (Figures 2c, 2d and Table 1); however, they were generally more stable in the organic solutions than [NBu_4_]_2_[Ce(NO_3_)_6_]. For SOs that are unstable in certain organic solvents, their half-lifetime stability are summarized in Table 1.

**Table 1:** Chemical Stability of the SOs in 12 Organic Solvents

	SO			
Solvent	[NBu_4_]_2_[Ce(NO_3_)_6_]	[NBu_4_][IO_4_]	[NBu_4_][HSO_5_]	[NBu_4_]_2_[S_2_O_8_]
DCM	◇ (4)	○^a^	◇ (8)^b^	○
IPA	△^c^	○	×^d^	◇ (6)
THF	△	○	○	◇ (1)
EA	○	○	×	×
Dioxane	△	○	×	◇ (6)
Acetone	△	△	○	◇ (2)
MeOH	○	○	◇ (4)	◇ (6)
EtOH	◇ (2)	○	○	◇ (2)
MeCN	◇ (6)	○	○	○
DMF	◇ (2)	○	◇ (6)	◇ (2)
DMA	◇ (4)	◇ (4)	◇ (2)	◇ (2)
DMSO	△	○	◇ (4)	◇ (6)

a ○ represents a stability of at least 12 h.

b ◇ represents a gradual decay within 12 h, where the number in parentheses represents the half-lifetime stability in h.

c △ represents complete decay in less than 1 h.

d × represents insolubility.

**Figure 3:**
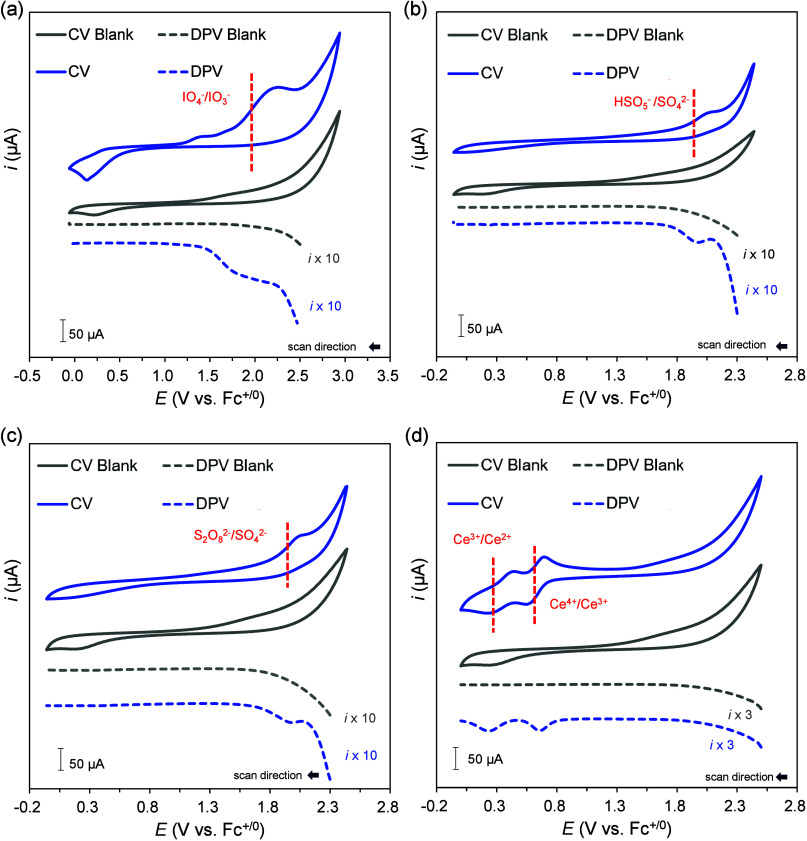
CV and DPV profiles of four SOs in MeCN (reference electrode, Ag^+^/Ag (0.01 M AgNO_3_)/0.1 M [NBu_4_][PF_6_] in MeCN; counter electrode, Pt wire; working electrode, Pt disk): (a) [NBu_4_][IO_4_], (b) [NBu_4_][HSO_5_], (c) [NBu_4_]_2_[S_2_O_8_], and (d) [NBu_4_]_2_[Ce(NO_3_)_6_].

The electrochemical behaviors of [NBu_4_]_2_[Ce(NO_3_)_6_], [NBu_4_][IO_4_], [NBu_4_][HSO_5_], and [NBu_4_]_2_[S_2_O_8_] were subsequently investigated by cyclic voltammetry (CV) and differential pulse voltammetry (DPV). Unless stated otherwise, all potentials referenced in this study are relative to the ferrocene/ferrocenium redox couple (Fc^+/0^), and all measurements were performed in triplicate to ensure the reproducibility and accuracy of the experimental results. The four modified SOs all exhibited oxidation peaks in MeCN; however, the results in other solvents varied (see Table 2 and Section S4b for the CV and DPV data for all 12 organic solvents). The CV curves of [NBu_4_][IO_4_] and [NBu_4_][HSO_5_] in MeCN exhibited irreversible redox features, which were used to identify their *E*_so_ values (Figures 3a, 3b). DPV provided sharper voltammetric peaks than those derived from CV; therefore, the DPV measurements helped to substantiate the *E*_so_ values, yielding *E*_so_ values of 1.99 and 1.83 V for [NBu_4_][IO_4_] and [NBu_4_][HSO_5_] in MeCN, respectively. Notably, [NBu_4_][IO_4_] and [NBu_4_][HSO_5_] showed only one anodic peak in most of the studied organic solvents, despite being recognized as two-electron oxidants in aqueous solutions.^14^ The second anodic potential of [NBu_4_][IO_4_] and [NBu_4_][HSO_5_] is possibly beyond the electrochemical window of most organic solvents.^31^ The oxidation of [NBu_4_]_2_[S_2_O_8_] manifested only in MeCN (Figure 3c and Section S4b). Furthermore, although CAN is widely considered a one-electron oxidant in redox catalysis, [NBu_4_]_2_[Ce(NO_3_)_6_] exhibited two quasi-reversible redox events in THF, acetone, and MeCN (Figure 3d and Section S4b). This likely resulted from the instability of [NBu_4_]_2_[Ce(NO_3_)_6_], leading to the formation of cerium-based oxide species.^14^ Additionally, in solvents where [NBu_4_]_2_[Ce(NO_3_)_6_] exhibited only a single oxidation peak (DCM, MeOH, DMF and DMSO), the *E*_so_ values were 0.27–0.63 V (0.92–1.28 V vs NHE); these values are several hundred millivolts lower than those previously reported for CAN in aqueous solutions.^14,32^ Overall, the CV and DPV measurements demonstrated that the solvent environment significantly impacted *E*_so_ in organic media.

**Table 2:** *E*_so_ Values of the Four SOs in Various Organic Solvents

	SO			
Solvent	[NBu_4_]_2_[Ce(NO_3_)_6_]	[NBu_4_][IO_4_]	[NBu_4_][HSO_5_]	[NBu_4_]_2_[S_2_O_8_]
DCM	0.49^a^	-	-	-
IPA	-	-	×^d^	-
THF	0.29^a^, 0.63^a^	-	-	-
EA	-	-	×	×
Dioxane	-	-	×	-
Acetone	0.41^b^, 0.53^a^	-	0.44^b^, 0.63^b^	-
MeOH	0.46^a^	-	-	-
EtOH	-	-	-	-
MeCN	0.27^a^, 0.58^a^	1.99^c^	1.83^c^	1.99^c^
DMF	0.51^b^	-	-	-
DMA	-	-	-	-
DMSO	0.51^b^	-	-	-

a Quasi-reversible redox wave.

b Irreversible redox wave.

c Only oxidative peak wave.

d × indicates insolubility in the solvent. The CV and DPV profiles are presented in Section S4.

### Thermodynamic Redox Potentials of H_2_O/O_2_ in Organic Solvents

For comparison with the *E*_so_ values of the four SOs, the *E*_H_2_O/O_2_(org)_ values were estimated from OCP measurements conducted using the different organic solvents. A four-electrode cell was constructed for the OCP measurements of *E*_H^+^/H_2__ versus Fc^+/0^ (Figure 1b and Section S5a).^20^ To prevent solvent evaporation, hydrogen gas was steadily bubbled through the cell via a purging tube for several minutes while stirring.^33^ The OCP was measured every second until the recorded potential showed no obvious fluctuations (<5 mV); this was continued for 60 s. Upon completion of the OCP measurements, Fc was introduced into each organic solvent, followed by CV to identify the Fc^+/0^ redox couple. Finally, the measured OCP was referenced to the Fc^+/0^ potential to reflect the actual reference electrode/potential used in the experiments.^20^

**Scheme 2:**
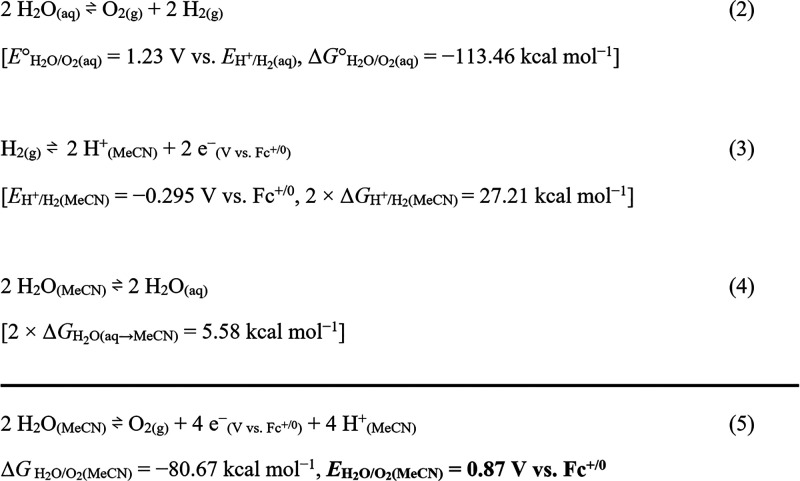
Representative Scheme for Estimating the *E*_H_2_O/O_2_(org)_ in MeCN via a Thermochemical Cycle

The thermochemical cycle for estimating *E*_H_2_O/O_2_(org)_ featured the following parameters (see eqs 2–4 in Scheme 2): (i) the redox potential of H_2_O/O_2_ relative to that of H^+^/H_2_ in the standard state (eq 2), (ii) the OCP of the H^+^/H_2_ redox couple in each organic solvent (eq 3), and (iii) the difference in solvation free energy of H_2_O between H_2_O and each organic solvent (eq 4). The +1.23 V value for (i; eq 2) was extracted from the literature.^34^ The *E*_H^+^/H_2__ values for the different organic solvents (ii, eq 3) were obtained via OCP measurements, with the potential referenced to Fc^+/0^ (Section S5b). The difference in the solvation free energy of H_2_O between H_2_O and each organic solvent (iii; eq 4) was calculated using density functional theory (DFT); this value contributed several kilocalories per mole (that is, 3–102 mV) to the resulting *E*_H_2_O/O_2_(org)_ (Section S5e). Finally, the sum of eqs 2–4 affords the *E*_H_2_O/O_2_(org)_ in organic media (eq 5 in Scheme 2). For example, the redox potential of H_2_O/O_2_ in MeCN (*E*_H_2_O/O_2_(MeCN)_) is derived to be 0.87 V vs Fc^+/0^. This thermodynamic analysis permits a comparison between *E*_H_2_O/O_2_(org)_ and *E*_so_ and guides the selection of the SO for the WOR. The *E*_H_2_O/O_2_(org)_ values obtained using the different anhydrous organic solvents are summarized in Table 3, and the OCP measurements of *E*_H^+^/H_2__ are provided in Section S5b.

**Table 3:**
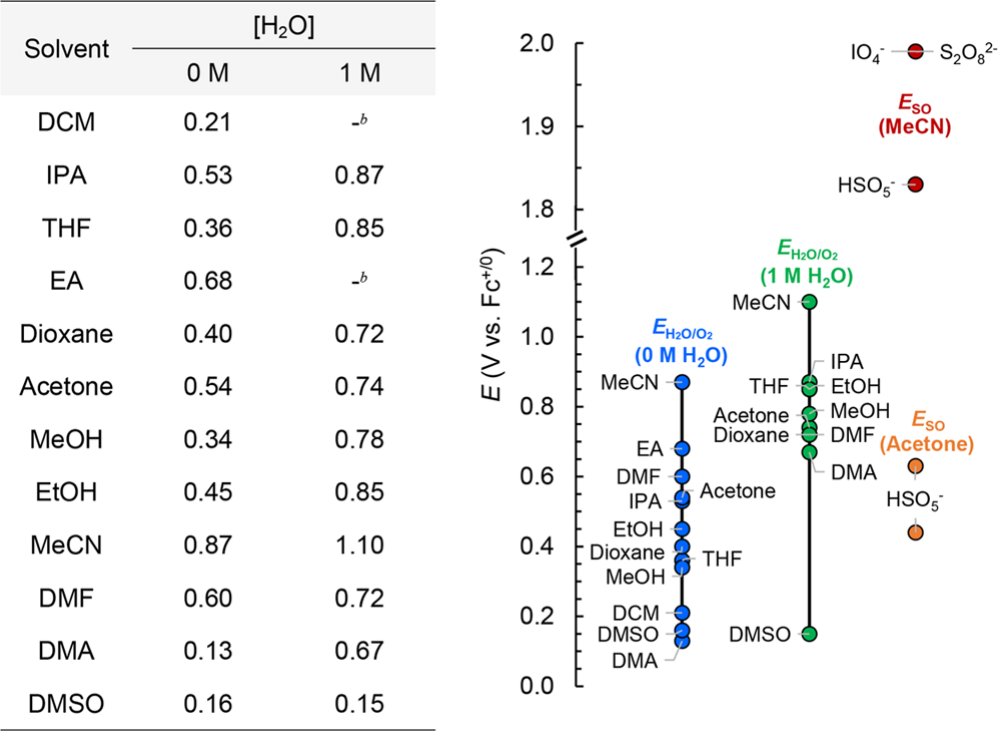
*E*_H_2_O/O_2_(org)_ Values of the 12 Investigated Organic Solvents in the Absence and Presence of 1 M [H_2_O] and a Comparison of *E*_H_2_O/O_2_(org)_ and *E*_so_^a^

a The *E*_so_ values of [NBu_4_]_2_[Ce(NO_3_)_6_] are not included in the comparison owing to its chemical instability.

b 1 M H_2_O is immiscible with DCM and EA.

Because H_2_O is the substrate in the WOR, the effects of the presence and concentration of H_2_O on the *E*_H_2_O/O_2_(org)_ value of the mixed water/organic solvent solutions must be investigated to further assess the feasibility of using the four SOs to drive the WOR. Therefore, *E*_H^+^/H_2__ was measured as a function of [H_2_O] for each organic solvent to establish a correlation between the OCP and [H_2_O]. In this series of OCP measurements, [H_2_O] was varied between 0 to 10 M (Section S5e), based on previously reported WOR studies conducted under various concentrations of H_2_O in organic media.^17^ Compared to the *E*_H_2_O/O_2_(org)_ values determined in the anhydrous organic solvents (i.e., 0 M H_2_O), the value for each mixed water/organic solvent under standard state conditions of 1 M H_2_O showed a substantial anodic shift (Table 3). This was presumably linked to 1) the increase in [H^+^] due to the self-ionization of H_2_O,^35^ which leads to *E*_H^+^/H_2__ becoming thermodynamically favorable, and 2) the resulting change in the OCP of the solution upon the introduction of H_2_O with a high dielectric constant.^36^

Tables 2 and 3 present some intriguing results. For example, the differences between *E*_H_2_O/O_2_(org)_ (0 M H_2_O) and the *E*_so_ values of [NBu_4_]_2_[S_2_O_8_] in acetone are within 100 mV (0.54 V vs 0.44 and 0.63 V); however, *E*_H_2_O/O_2_(Acetone)_ in the presence of 1 M H_2_O exceeds the *E*_so_ values of [NBu_4_]_2_[S_2_O_8_]. This finding suggests that the utilization of [NBu_4_]_2_[S_2_O_8_] as an SO for the chemical WOR needs to consider the presence of H_2_O. Additionally, regardless of its stability issues, the *E*_so_ value(s) of [NBu_4_]_2_[Ce(NO_3_)_6_] were 0.21–0.52 V lower than *E*_H_2_O/O_2_(org)_ under standard state conditions of 1 M H_2_O for most of the organic solvents (Tables 2, 3). This indicates that [NBu_4_]_2_[Ce(NO_3_)_6_] is not an ideal SO for the chemical WOR in most organic solvents. In MeCN, the highest *E*_so_ values (1.8–2.0 V) were observed for [NBu_4_][IO_4_], [NBu_4_][HSO_5_], and [NBu_4_]_2_[S_2_O_8_], indicating that these three SOs are well-suited for studying the chemical WOR in the solution of MeCN.

To broaden the understanding of *E*_H_2_O/O_2_(org)_ and the applications of the WOR under acidic and alkaline conditions in organic media, protonated DMF ([DMF-H][OTf], where OTf is triflate) and NaOH were added to each organic solvent at different concentrations, following which *E*_H^+^/H_2__ was determined via OCP measurements and *E*_H_2_O/O_2_(org)_ was derived (Section S6). *E*_H_2_O/O_2_(org)_ was found to be affected by [[DMF-H][OTf]] and [NaOH], indicating that the [acid] and [base] levels should also be considered when selecting an SO for the chemical WOR.

### Insights into Molecular Water-Oxidation Catalysts in Organic Media: Rate–Overpotential Free-Energy Relationship

**Figure 4:**
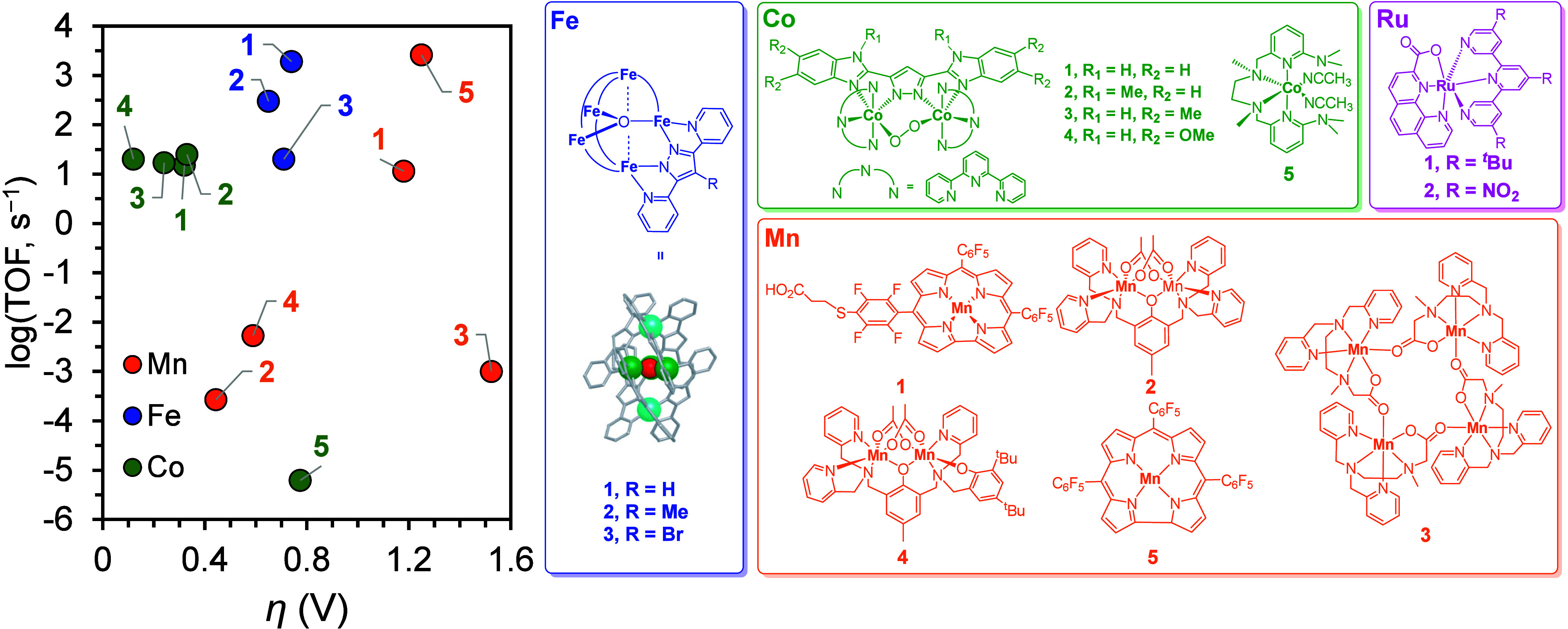
log(TOF)−η plot of previously reported MWOCs in organic solutions.

The normalized kinetic and thermodynamic descriptors, i.e., the turnover frequency (TOF) and η, are both used to evaluate the efficacy of molecular (electro)catalysts, albeit under different reaction conditions.^17,37^ The η value of molecular (electro)catalysts, which can be estimated using eq 6, is defined as the difference between the catalytic potential (*E*_cat_; catalysis-initiating potential or the half-wave potential associated with the reaction) and the thermodynamic potential of the specific reaction (*E*_rxn_).^17,38^ The relationship between log(TOF) and η provides insights into the catalytic performance of MWOCs. Conceptually, this log(TOF)−η relationship is similar to a cost (η)–gain (log(TOF)) relationship; that is, a good electrocatalyst should manifest a high TOF with minimal η.^22,39,40^ Several attempts have been made to examine the catalyst performance of precedent MWOCs^26,41−50^ in organic media via log(TOF)−η analysis. First, we derived the *E*_H_2_O/O_2_(org)_ values of MWOCs under previously reported experimental conditions from the corresponding OCP measurements of *E*_H^+^/H_2__ according to Scheme 2 (eqs 2–5 and Section S6d), and their η values were then calculated using eq 6, where *E*_rxn_ = *E*_H_2_O/O_2_(org)_. The *E*_cat_ and TOFs of these MWOCs were retrieved from the original studies as well (Table 4). These efforts enabled the previously reported MWOCs in organic media to be benchmarked against the log(TOF)−η relationship (Figure 4).
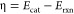
6

**Table 4:** Catalytic Performances of Previously Reported MWOCs in Organic Media^j^

	*E*_cat_						
Complex	redox couple	mV	*E*_H_2_O/O_2__ (mV)	η (mV)	TOF (s^–1^)	Solvent	ref
**Mn-1**	Mn^4+^/Mn^5+^	1380	200	1180	1.1 × 10^1^	MeCN^b^	(45)
**Mn-2**	Mn^2+^Mn^2+^/Mn^2+^Mn^3+^	1254	810	444	2.7 × 10^–4^	MeCN^c^	(41)
**Mn-3**	Mn^3+^/Mn^4+^	1944	420	1524	1.0 × 10^–3^	MeCN^d^	(43)
**Mn-4**	Mn^2+^Mn^2+^/Mn^2+^Mn^3+^	1399	810	589	5.3 × 10^–3^	MeCN^c^	(42)
**Mn-5**	Mn^4+^/Mn^5+^	1560	310	1250	2.6 × 10^3^	PC^i^	(49)
**Fe-1**	Fe_4_^3+^Fe_1_^2+^/Fe_5_^3+^	1824	-h	740^a^	1.9 × 10^3^	MeCN^e^	(44)
**Fe-2**	Fe_4_^3+^Fe_1_^2+^/Fe_5_^3+^	1734	-h	650^a^	3.0 × 10^2^	MeCN^e^	(50)
**Fe-3**	Fe_4_^3+^Fe_1_^2+^/Fe_5_^3+^	1794	-h	710^a^	2.0 × 10^1^	MeCN^e^	(50)
**Co-1**	[cat]^3+^/[cat]^4+^	1090	770	320	4.0 × 10^1^	MeCN^e^	(26)
**Co-2**	[cat]^3+^/[cat]^4+^	1100	770	330	2.5 × 10^1^	MeCN^e^	(26)
**Co-3**	[cat]^3+^/[cat]^4+^	1010	770	240	1.3 × 10^1^	MeCN^e^	(48)
**Co-4**	[cat]^3+^/[cat]^4+^	890	770	120	1.7 × 10^1^	MeCN^e^	(48)
**Co-5**	Co^2+^/Co^3+^	1584	810	774	6.2 × 10^–6^	MeCN^f^	(47)
**Ru-1**	Ru^3+^/Ru^4+^	1424	800	624	-	MeCN^g^	(46)
**Ru-2**	Ru^3+^/Ru^4+^	1564	800	764	-	MeCN^g^	(46)

a The η values of **Fe-1** to **Fe-3** were retrieved from the original papers.

b 25 mM NaOH in MeCN.

c 10% (v/v) H_2_O in MeCN.

d 6 mM lutidine +1 M H_2_O in MeCN.

e 5 M H_2_O in MeCN.

f 5% (v/v) H_2_O in MeCN.

g 3 M H_2_O in MeCN.

h Experimental conditions not provided in the original papers.

i PC: propylene carbonate.

j All studies were conducted via the electrochemical method.

As exhibited in Figure 4, pentanuclear Fe complexes (**Fe-1** to **Fe-3**) exhibited the highest efficacy among the investigated MWOCs (Table 4, Figure 4): their TOFs were several orders of magnitude higher than those of the Co- and Mn-based MWOCs at similar η values (100–500 mV; **Co**-**1** to **Co-5**, **Mn-2**, and **Mn-4**). This result suggested that the cooperativity between the active sites of **Fe-1** to **Fe-3** significantly enhanced the catalytic activity.^44^ The reaction mechanisms of **Co-1** to **Co-4** can be altered by the alkalinity of the reaction system, circumventing the trade-off between log(TOF) and η.^48^ For **Mn-1**, **Mn-2**, **Mn-4**, and **Mn-5**,^49^ the apparent linear relationship between log(TOF) and η suggests that these monomeric Mn-based MWOCs have similar turnover-limiting steps. **Mn-5** exhibited relatively good activity in the high-η regime (that is, η ≥ 1 V); thus, rational catalyst design may further enhance the effectiveness of Mn-based MWOCs.^17^ The monomeric Ru complexes, **Ru-1** and **Ru-2**, yielded η values of approximately 650–750 mV for the WOR; unfortunately, they could not be incorporated into the log(TOF)−η analysis because their TOFs were not reported.^46^ Most Ru-based MWOCs have been investigated and benchmarked under aqueous conditions.^51^

Our analysis indicates that [NBu_4_][IO_4_], [NBu_4_][HSO_5_], and [NBu_4_]_2_[S_2_O_8_] are potent SOs for the WOR in MeCN because their *E*_so_ values are significantly more positive than the *E*_cat_ values of most other MWOCs (Tables 2, 3). It is probably not a coincidence that MeCN is the solvent of choice in most WOR studies conducted in nonaqueous solvents, as shown in Table 4. The high polarity of MeCN allows most MWOCs and, most importantly, water, to dissolve in it, resulting in a homogeneous reaction solution. Additionally, MeCN is resistant to background oxidation up to 2.35–2.80 V vs Fc^+/0^, depending on the electrolyte.^31,52^ This potential window is suitable for diverse MWOCs and superior to most other organic solvents.

## Conclusions

In summary, the cations of CAN, sodium periodate, potassium peroxymonosulfate, and sodium peroxydisulfate were replaced with [NBu_4_]^+^ to evaluate their appropriateness as SOs for the WOR in nonaqueous solvents. The electrochemical properties and chemical stabilities of the SOs were examined in 12 organic solvents to facilitate practical applications such as oxidative organic transformation and redox catalysis. The *E*_H_2_O/O_2_(org)_ value in each organic solvent was derived using the *E*_H^+^/H_2__ redox potential via OCP measurements and the related thermochemical cycle. We expect that a deep understanding of *E*_H_2_O/O_2_(org)_ can promote the investigation of WOR in organic media. Unsurprisingly, *E*_H_2_O/O_2_(org)_ was found to be influenced by [H_2_O], [[DMF-H][OTf]], and [NaOH]. Therefore, the SO for the chemical WOR should be selected considering the *E*_so_, *E*_H_2_O/O_2_(org)_, and *E*_cat_ values under the desired reaction conditions. For [NBu_4_][IO_4_], [NBu_4_][HSO_5_], and [NBu_4_]_2_[S_2_O_8_] in MeCN, the highest *E*_so_ values were observed, and *E*_so_ was significantly more positive than *E*_H_2_O/O_2_(MeCN)_. This outcome suggests that these three SOs are ideal for driving the chemical WOR in MeCN. log(TOF)−η analysis performed using the *E*_H_2_O/O_2_(org)_ values helped to benchmark previously reported MWOCs operating in organic solutions based on kinetics and thermodynamics. Different metal-based WOCs exhibit distinct log(TOF)/η dependency, thus, providing valuable indications to guide catalyst design. Overall, the method and values reported herein are expected to propel investigations on MWOCs in diverse organic solvents and provide the necessary thermochemical data to advance redox catalysis and energy conversion under nonstandard state conditions.

## Experimental Section

### Synthesis of Sacrificial Oxidants

The SOs—[NBu_4_]_2_[Ce(NO_3_)_6_], [NBu_4_][IO_4_], [NBu_4_][HSO_5_], and [NBu_4_]_2_[S_2_O_8_]—were synthesized according to the literature with some modifications.^26−29^ The details and characterization data are provided in the Supporting Information.

### Electrochemistry

Electrochemical experiments were conducted using a three-electrode setup with a PalmSens4 potentiostat connected to a computer running PSTrace software. All voltammetric measurements were performed under a nitrogen atmosphere using a standard three-electrode setup, which featured a platinum disk working electrode, platinum wire auxiliary electrode, Ag wire pseudoreference electrode with 0.01 M Ag^+^ ions (that is, AgNO_3_ or AgPF_6_), and 0.1 M [NBu_4_]^+^ salts (that is, [NBu_4_][PF_6_] or [NBu_4_][BF_4_]) as the electrolyte. Each solution was supplemented with 0.1 M [NBu_4_][PF_6_] as a supporting electrolyte. The working electrode was polished with 0.05 μm alumina on a wetted Buehler felt pad between each CV, DPV, and OCP experiment. All voltammograms were internally referenced to the redox potential of Fc^+/0^. The laboratory temperature was maintained at 25 ± 2 °C.

### Computational Methods

DFT calculations were performed using Orca (version 5.0.4)^53^ to examine the transfer energy of water in various solvents. Geometry optimization was conducted using the TPSS^54^ functional with D4^55^ dispersion correction, along with the 6-31G*^56^ basis set for all atoms. Single-point calculations were performed using the wB97X^57^ functional combined with the reparameterized D4 dispersion correction, with the def2-TZVPP^58^ basis set applied to all atoms. Solvent effects were accounted for using the SMD^59^ method for each specific solvent.

## Data Availability

The data underlying this study are available in the published article and its Supporting Information.

## Supporting Information Available

Electrochemical analysis; OCP measurements for determining *E*_H^+^__/H_2__; thermodynamic potentials for *E*_H_2_O/O_2__; ^1^H NMR, ^13^C NMR, and electrospray ionization mass spectra of the synthesized sacrificial oxidants; UV–vis spectra of synthesized sacrificial oxidants (PDF)
