# Supplementary material for: Thermodynamic Assessment of Sacrificial Oxidant Potential, H2O/O2 Potential, and Rate–Overpotential Relationship to Examine Catalytic Water Oxidation in Nonaqueous Solvents
Source: Inorg Chem. 2024 Nov 11;63(47):22523–31. doi: 10.1021/acs.inorgchem.4c03897 (PMC11600503; doi:10.1021/acs.inorgchem.4c03897)
Supplement: Supplementary file 1 — ic4c03897_si_001.pdf [file ic4c03897_si_001.pdf]

Supporting Information

Thermodynamic Assessment of Sacrificial Oxidant Potential, H_2O/O_2 Potential, and Rate–Overpotential Relationship to Examine Catalytic Water Oxidation in Non-aqueous Solvents

Shun-Chien Hsiao, Ting-Yi Chuang, Sharad V. Kumbhar, Tzuhsiung Yang, and Yu-Heng Wang*

*Email: wangyh2@mx.nthu.edu.tw

*Department of Chemistry, National Tsing Hua University, Hsinchu 30013, Taiwan

Table of Contents

1. General Considerations 3

2. Synthesis of Chemical Oxidant 3

3. UV-Vis Spectral Measurements 5

3a. General Considerations 5
3b. UV-Vis absorption spectra of $[NBu_4]_2[Ce(NO_3)_6]$ 5
3c. UV-Vis absorption spectra of $[NBu_4][IO_4]$ 7
3d. UV-Vis absorption spectra of $[NBu_4][HSO_5]$ 9
3e. UV-Vis absorption spectra of $[NBu_4]_2[S_2O_8]$ 11

4. Electrochemical Experiments 13

4a. General Considerations 13
4b. Cyclic voltammograms and differential pulse voltammograms of oxidants in different solvents 14

5. Estimation of the Thermodynamic Reduction Potential of O_2/H_2O at Non-standard State (aqueous) 21

5a. General Considerations 21
5b. The OCP measurement of H^+/H_2 redox couples (E_{H^+/H_2}) in various organic solvents 22
5c. The OCP measurement of H^+/H_2 redox couples (E_{H^+/H_2}) under neutral conditions (H_2O: 0.01–10 M) 26
5d. Gibbs free energies of H_2O transfer from H_2O to different organic solvents 33
5e. Estimation of E_{H_2O/O_2} based on OCP measurements of E_{H^+/H_2} 37

6. Estimation of the Thermodynamic Reduction Potential of O_2/H_2O at Non-standard State (acidic and alkaline conditions) 39

6a. General Considerations 39
6b. Thermodynamic Redox Potentials of H_2O/O_2 in Acidic and Alkaline Nonaqueous Media 40
6c. The OCP measurement of H^+/H_2 redox couples (E_{H^+/H_2}) under acidic conditions ([DMF-H][OTf]: 1–1000 mM) 41
6d. The OCP measurement of H^+/H_2 redox couples (E_{H^+/H_2}) under alkaline conditions (NaOH: 1–100 mM) 49
6e. Estimation of E_{H_2O/O_2} based on OCP measurements of E_{H^+/H_2} 59

7. Compound Spectra 61

1. General Considerations

All commercially available reagents were used as received, except where otherwise noted.^{1}H, ^{13}C, NMR spectra were recorded on a Bruker Avance III 500 spectrometer (^{1}H 500.1 MHz, ^{13}C 125.7 MHz). Chemical shifts are reported in parts per million (ppm), referenced to CD_3CN at 1.94 ppm (^{1}H) and 1.32 ppm (^{13}C. Multiplicities are described using the following abbreviations: s = singlet, bs = broad singlet, d = doublet, dd = doublet of doublet, t = triplet, m = multiplet. NMR spectra were plotted with MestReNova v14.2.0-26256 (MestreLab Research S. L. 2020). UV-visible spectra were recorded on a Varian Cary 60 spectrometer. Elemental analysis was conducted by Elementar vario EL CUBE CHN-OS Rapid EA000100. High-resolution electrospray ionization mass spectra (HR-ESI-MS) were recorded on a VARIAN 901-MS (FT-ICR Mass) mass spectrometer.

2. Synthesis of Chemical Oxidant

A. Synthesis of $[NBu_4]_2[Ce(NO_3)_6]$

$[NBu_4]_2[Ce(NO_3)_6]$ was synthesized according to the literature protocol.[1] A solution of $(NH_4)_2Ce(NO_3)_6$ (0.5 g, 0.9 mmol) in H_2O (3 mL) was treated with a solution of NBu_4Br (0.59 g, 1.8 mmol) in H_2O (1 mL) to give a fine orange precipitate. The mixture was stirred for 10 minutes, filtered, and the yellow solid was washed with diethyl ether and dried in vacuo. Yield: 0.79 g (87%). HR-ESI-MS (*m*/*z*, positive): $[NBu_4]^+$ calcd for $C_{16}H_{36}N_1$: 242.28477; found: 242.28478. ^{1}H NMR (400 MHz, CD_3CN): δ 3.14 – 3.06 (m, 8H), 1.66 – 1.56 (m, 8H), 1.36 (q, *J* = 7.4 Hz, 8H), 0.97 (td, *J* = 7.2, 1.5 Hz, 12H). $^{13}C\{^{1}H\}$ NMR (125 MHz, CD_3CN): δ 59.3, 24.2, 20.2, 13.7.

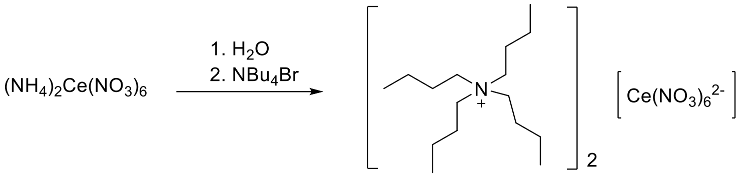

B. Synthesis of $[NBu_4][IO_4]$

$[NBu_4][IO_4]$ was synthesized according to the literature protocol.[2] Sodium periodate (l.1 g, 5 mmol) and NBu_4Br (1.6 g, 5 mmol) were dissolved in a minimal amount of water, respectively, and mixed at 0 °C. Whereby a white precipitate separated immediately. After vigorous stirring for 30 min, the white precipitate was filtered and dried under a vacuum. Yield: 1.77 g (83%). HR-ESI-MS (*m*/*z*, positive): $[NBu_4]^+$ calcd for $C_{16}H_{36}N_1$: 242.28477; found: 242.28485. HR-ESI-MS (*m*/*z*, negative) $[IO_4]^-$ calcd for IO_4: 190.8841; found: 190.8923. ^{1}H NMR (400 MHz, CD_3CN): δ 3.13 – 3.04 (m, 8H), 1.60 (tt, *J* = 8.2, 6.1 Hz, 8H), 1.35 (q, *J* = 7.4 Hz, 8H), 0.97 (t, *J* = 7.3 Hz, 12H). $^{13}C\{^{1}H\}$ NMR (125 MHz, CD_3CN): δ 59.3, 24.2, 20.2, 13.7.

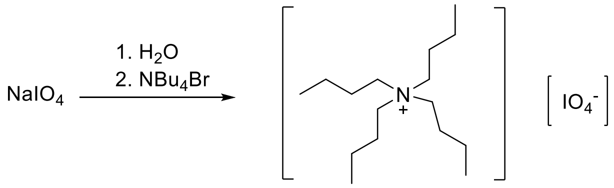

C. Synthesis of $[NBu_4][HSO_5]$

$[NBu_4][HSO_5]$ was synthesized according to the literature protocol.[3] A solution of Oxone (1.0 g, 3.25 mmol) in water (10 mL) was stirred with tetrabutylammonium hydrogen sulfate (1.0 g, 2.95 mmol) for 20 min. The solution was extracted with CH_2Cl_2 (20 mL), and the organic phase was dried over magnesium sulfate and filtered. After evaporation of the solvent, the remaining white solid was washed with hexane (5 mL) and dried under a vacuum. Yield: 0.75 g (75%). HR-ESI-MS

(*m/z*, positive): $[NBu_4]^+$ calcd for $C_{16}H_{36}N_1$: 242.2847; found: 242.2848. HR-ESI-MS (*m/z*, negative): $[HSO_5]^-$ calcd for HSO_5: 112.9544; found: 112.9508. ^{1}H NMR (400 MHz, CD_3CN): δ 3.12 – 3.04 (m, 8H), 1.60 (tt, *J* = 8.2, 6.0 Hz, 8H), 1.35 (h, *J* = 7.4 Hz, 8H), 0.97 (t, *J* = 7.3 Hz, 12H). ^{13}C{^{1}H} NMR (125 MHz, CD_3CN): δ 59.2, 24.2, 20.2, 13.7.

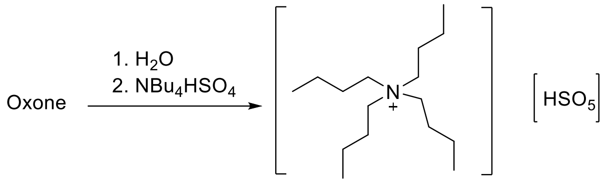

D. Synthesis of $[NBu_4]_2[S_2O_8]$

$[NBu_4]_2[S_2O_8]$ was synthesized according to the literature protocol.[4] $(NBu_4)HSO_4$ (0.8 g, 2.95 mmol) and $K_2S_2O_8$ (2 g, 5.9 mmol) were dissolved in distilled water (10 mL), and the mixture was stirred for 30 min at rt. The solution was extracted with DCM, and the combined organic layers were washed with water, dried over $MgSO_4$, filtered, and concentrated under reduced pressure to afford a white solid. Yield: 1.58 g (79 %). HR-ESI-MS (*m/z*, positive): $[NBu_4]^+$ calcd for $C_{16}H_{36}N_1$: 242.2847; found: 242.2846. ^{1}H NMR (400 MHz, CD_3CN): δ 3.15 – 3.07 (m, 8H), 1.61 (p, *J* = 7.9 Hz, 8H), 1.37 (p, *J* = 7.3 Hz, 8H), 0.97 (t, *J* = 7.3 Hz, 12H). ^{13}C{^{1}H} NMR (125 MHz, CD_3CN): δ 59.2, 24.2, 20.2, 13.7. Elem. Anal. Calc. (%) for $C_{32}H_{72}N_2O_8S_2$: C, 56.77, H, 10.72, N, 4.14, O, 18.90, S, 9.47. Found: C, 56.48, H, 10.78, N, 4.05, O, 19.00, S, 9.53.

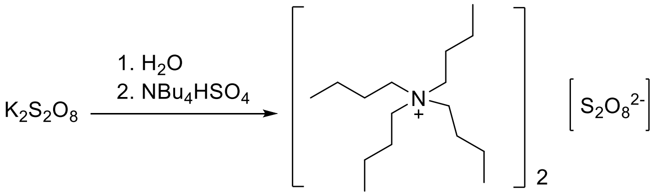

E. Synthesis of [DMF-H][OTf]

[DMF-H][OTf] was prepared according to literature procedures.[5] *N*, *N*-Dimethylformamide (3.1 mL, 40 mmol) was added in DCM (ca. 10 mL), and the mixture was cooled to 0 ˚C. Triflic acid (2.95 mL, 33.3 mmol) was added to the above mixture dropwise and stirred at room temperature for 30 min. DCM was removed by vacuum, and a white slurry was obtained. Diethyl ether (10 mL × 3) was added and evaporated by vacuum to remove the residual DMF. The white solid product was collected by filtration, washed with diethyl ether, and dried under a high vacuum. The ^{1}H NMR spectrum of [DMF-H][OTf] in DMSO-d_6 compared to DMF under the same conditions showed a shift of the $–NMe_2$ singlets from 2.89 and 2.73 to 2.88 and 2.72 ppm. The H at carbonyl group of DMF in DMSO-d_6 at 7.95 shifts to 7.94 in [DMF-H][OTf]. ^{19}F NMR spectrum of [DMF-H][OTf] in DMSO-d_6 shows a singlet at -77.76 ppm, different from triflic acid at -78.36 ppm under the same conditions. Yield: 2.30 g (31 %). ^{1}H NMR (500 MHz, DMSO-d_6): δ 10.45 (s, 1H), 7.94 (s, 1H), 2.88 (s, 3H), 2.72 (s, 3H). ^{13}C{^{1}H} NMR (125 MHz, DMSO-d_6): δ 162.4, 124.6, 122.1, 119.5, 116.9, 40.0, 39.9, 39.7, 39.5, 39.4, 39.2, 39.0, 35.9, 30.9. ^{19}F{^{1}H} NMR (470 MHz, DMSO-d_6): δ -77.77.

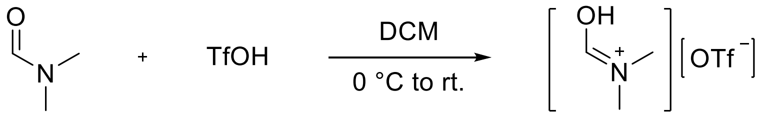

3. UV-Vis Spectral Measurements

3a. General Considerations

UV-Vis spectroscopy measurements were performed with a Cary 60 spectrometer at 25 °C using a 1 cm path-length quartz cell. Measure blank correction first, introduce the respective oxidants, and measure the wavelength range from the cutoff wavelength of each solvent to 600 nm, scanning every two hours until 12 hours.

Table S1. Full name of each solvent abbreviation.

Abbreviation	Full Name
DCM	Dichloromethane
IPA	Isopropanol
THF	Tetrahydrofuran
EA	Ethyl acetate
Dioxane	Dioxane
Acetone	Acetone
MeOH	Methanol
EtOH	Ethanol
MeCN	Acetonitrile
DMF	*N*, *N*-Dimethylformamide
DMA	Dimethylacetamide
DMSO	Dimethyl sulfoxide

3b. UV-Vis absorption spectra of $[NBu_4]_2[Ce(NO_3)_6]$

The UV-Vis absorption spectra of $[NBu_4]_2[Ce(NO_3)_6]$ in anhydrous solvents are shown in Figure S1.

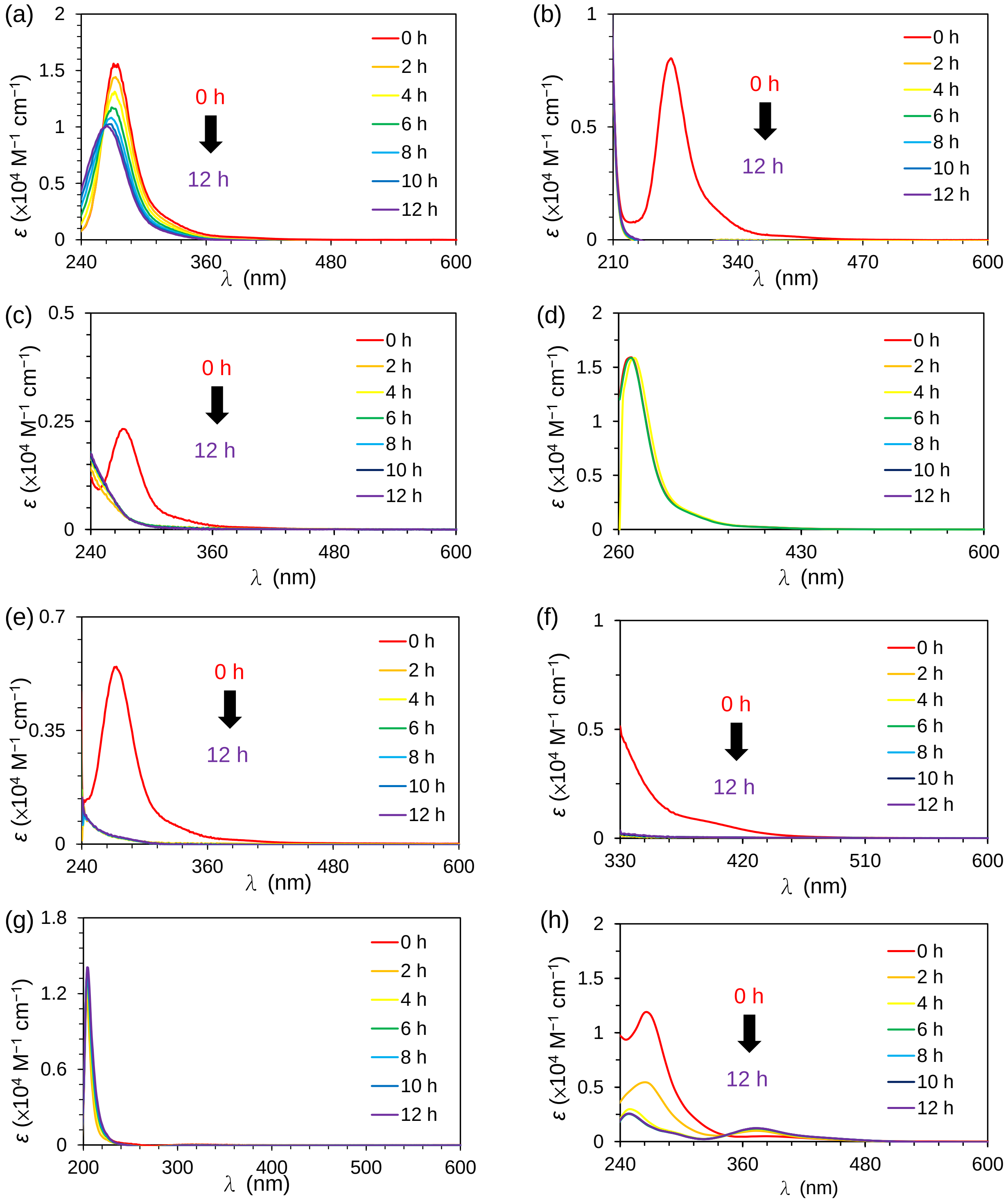
(a)
(b)
(c)
(d)
(e)
(f)
(g)
(h)
0 h
12 h
0 h
2 h
4 h
6 h
8 h
10 h
12 h
ε (×10⁴ M⁻¹ cm⁻¹)
λ (nm)

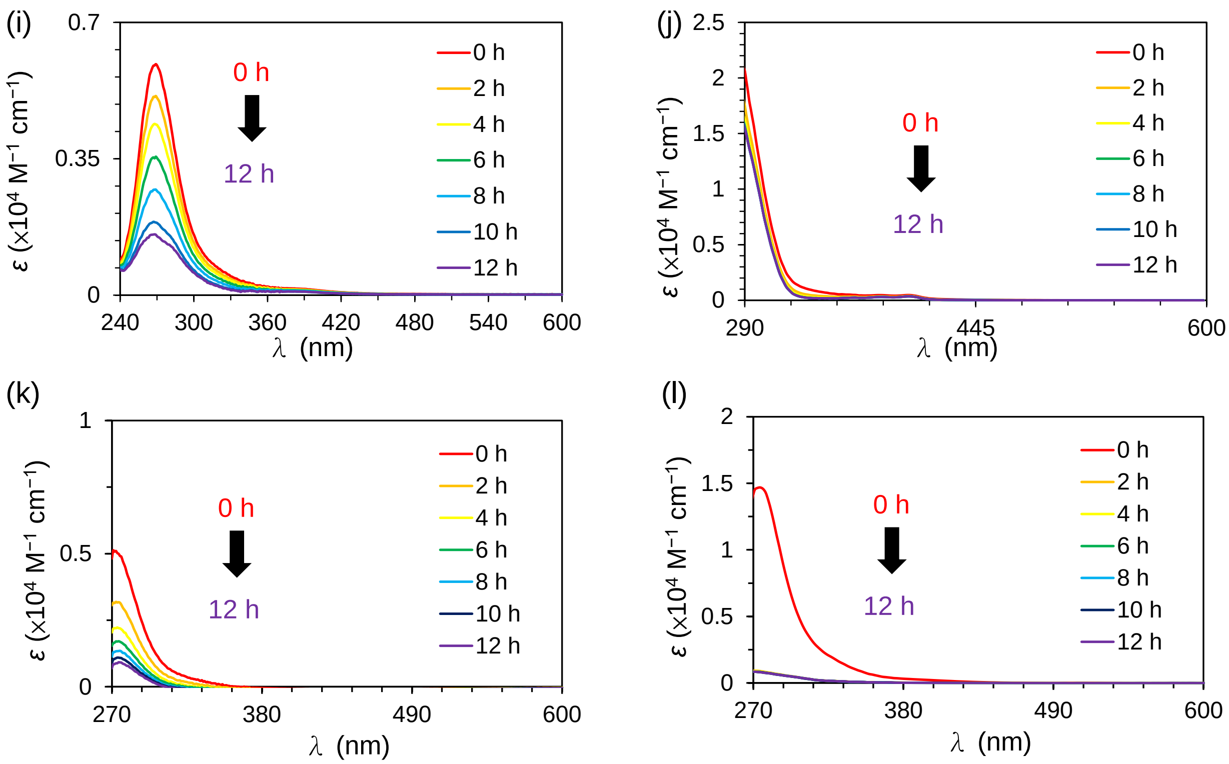

Figure S1. UV-Vis absorption spectra of $[NBu_4]_2[Ce(NO_3)_6]$ in (a) DCM, (b) IPA, (c) THF, (d) EA, (e) Dioxane, (f) Acetone, (g) MeOH, (h) EtOH, (i) MeCN, (j) DMF, (k) DMA, and (l) DMSO.

3c. UV-Vis absorption spectra of $[NBu_4][IO_4]$

The UV-Vis absorption spectra of $[NBu_4][IO_4]$ in anhydrous solvents are shown in Figure S2.

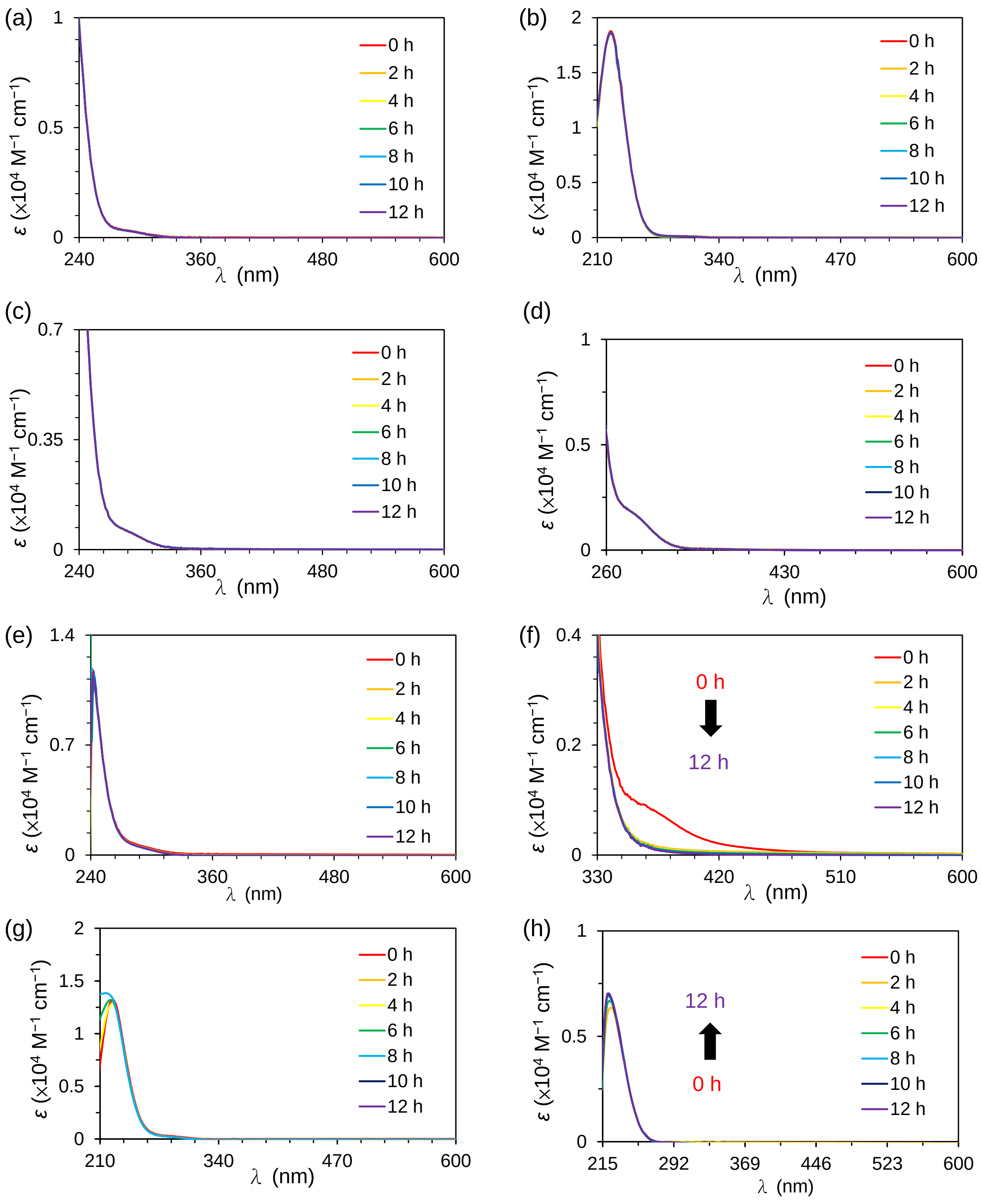
(a)
(b)
(c)
(d)
(e)
(f)
(g)
(h)
ε (×10⁴ M⁻¹ cm⁻¹)
λ (nm)
0 h
2 h
4 h
6 h
8 h
10 h
12 h
0 h
12 h
12 h
0 h

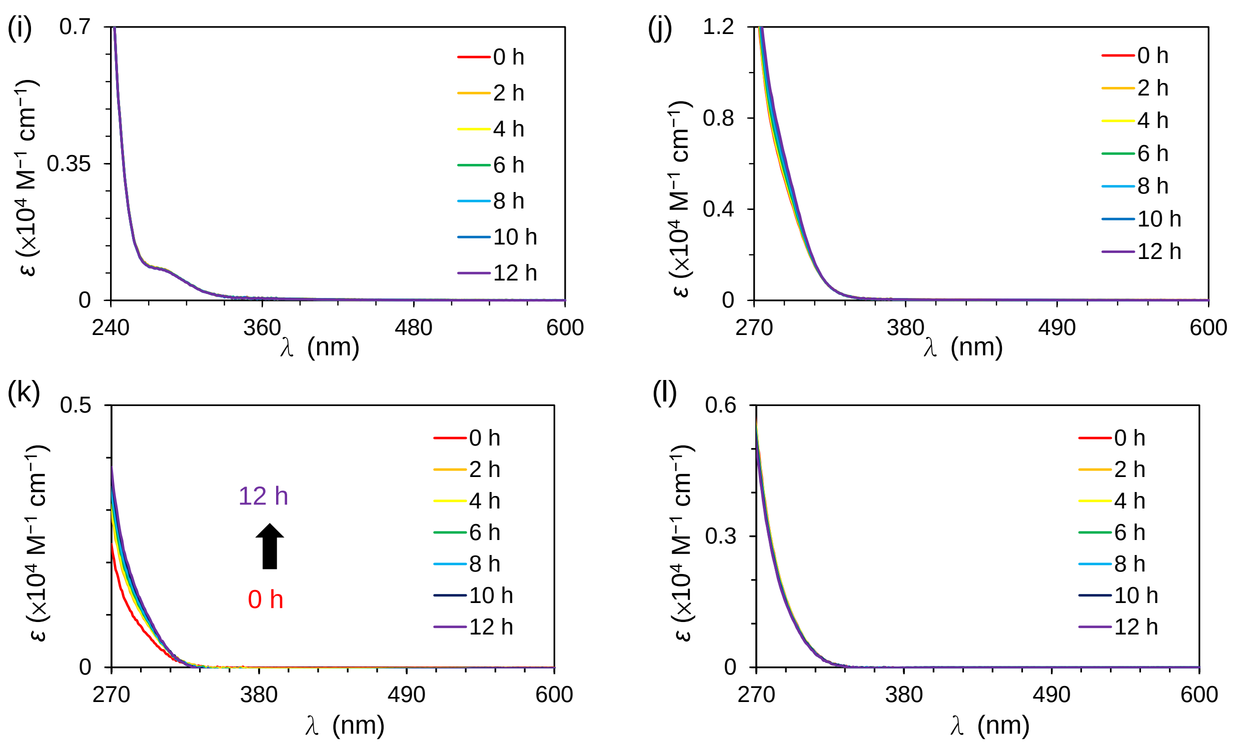

Figure S2. UV-Vis absorption spectra of $[NBu_4][IO_4]$ in (a) DCM, (b) IPA, (c) THF, (d) EA, (e) Dioxane, (f) Acetone, (g) MeOH, (h) EtOH, (i) MeCN, (j) DMF, (k) DMA, and (l) DMSO.

3d. UV-Vis absorption spectra of $[NBu_4][HSO_5]$

The UV-Vis absorption spectra of $[NBu_4][HSO_5]$ in anhydrous solvents are shown in Figure S3.

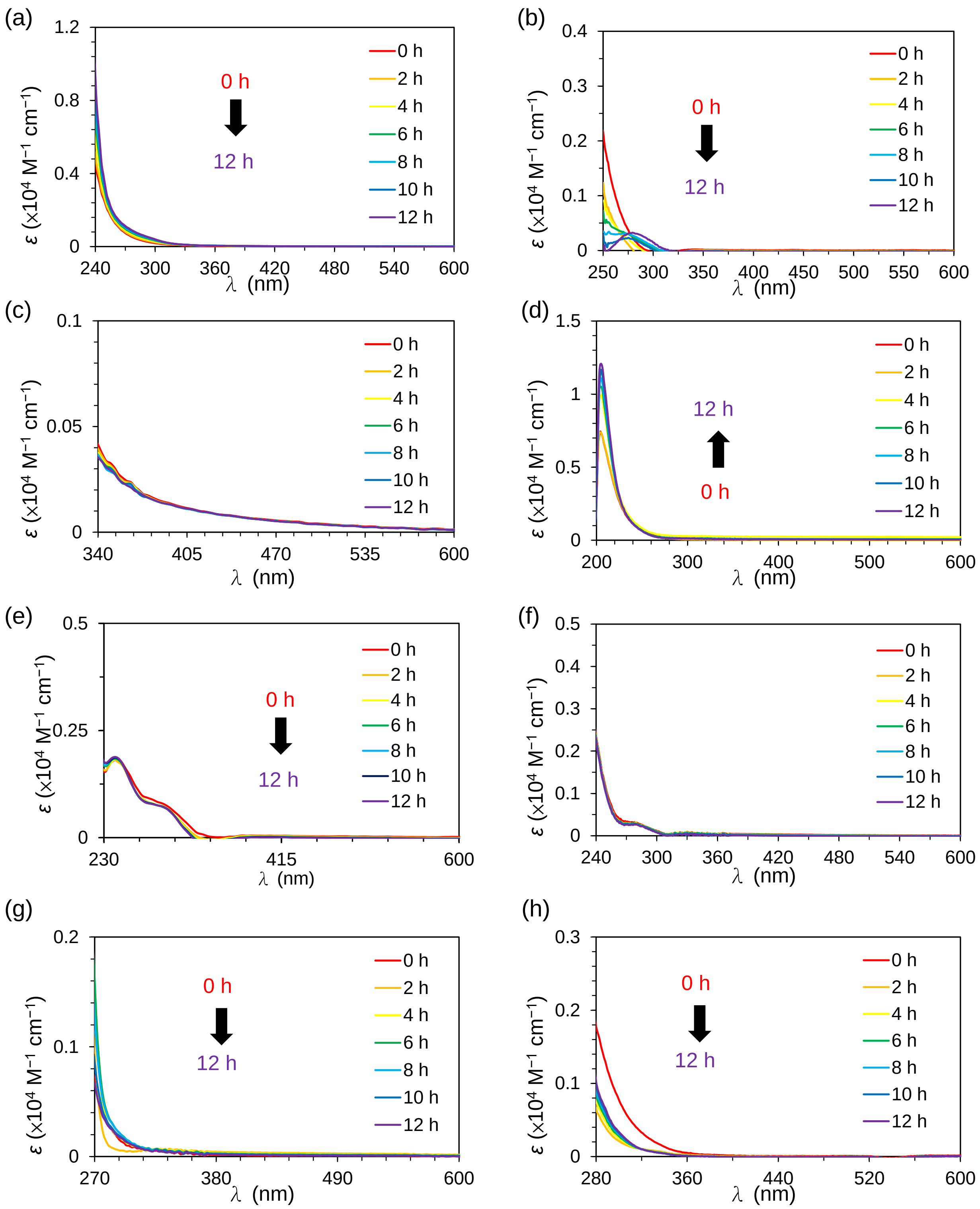
(a)
1.2
0.8
0.4
0
ε (×10⁴ M⁻¹ cm⁻¹)
240
300
360
420
480
540
600
λ (nm)
0 h
12 h
0 h
2 h
4 h
6 h
8 h
10 h
12 h
(b)
0.4
0.3
0.2
0.1
0
ε (×10⁴ M⁻¹ cm⁻¹)
250
300
350
400
450
500
550
600
λ (nm)
0 h
12 h
0 h
2 h
4 h
6 h
8 h
10 h
12 h
(c)
0.1
0.05
0
ε (×10⁴ M⁻¹ cm⁻¹)
340
405
470
535
600
λ (nm)
0 h
2 h
4 h
6 h
8 h
10 h
12 h
(d)
1.5
1
0.5
0
ε (×10⁴ M⁻¹ cm⁻¹)
200
300
400
500
600
λ (nm)
12 h
0 h
0 h
2 h
4 h
6 h
8 h
10 h
12 h
(e)
0.5
0.25
0
ε (×10⁴ M⁻¹ cm⁻¹)
230
415
600
λ (nm)
0 h
12 h
0 h
2 h
4 h
6 h
8 h
10 h
12 h
(f)
0.5
0.4
0.3
0.2
0.1
0
ε (×10⁴ M⁻¹ cm⁻¹)
240
300
360
420
480
540
600
λ (nm)
0 h
2 h
4 h
6 h
8 h
10 h
12 h
(g)
0.2
0.1
0
ε (×10⁴ M⁻¹ cm⁻¹)
270
380
490
600
λ (nm)
0 h
12 h
0 h
2 h
4 h
6 h
8 h
10 h
12 h
(h)
0.3
0.2
0.1
0
ε (×10⁴ M⁻¹ cm⁻¹)
280
360
440
520
600
λ (nm)
0 h
12 h
0 h
2 h
4 h
6 h
8 h
10 h
12 h

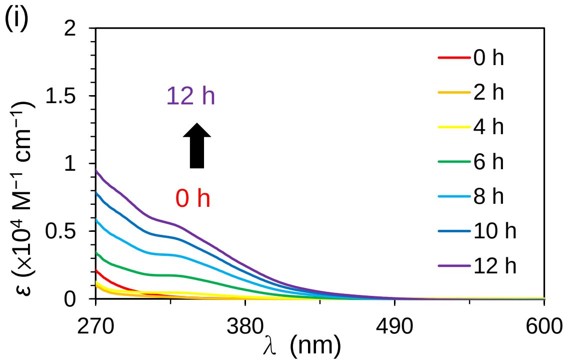

Figure S3. UV-Vis absorption spectra of $[NBu_4][HSO_5]$ in (a) DCM, (b) THF, (c) Acetone, (d) MeOH, (e) EtOH, (f) MeCN, (g) DMF, (h) DMA and (i) DMSO.

3e. UV-Vis absorption spectra of $[NBu_4]_2[S_2O_8]$

The UV-Vis absorption spectra of $[NBu_4]_2[S_2O_8]$ in anhydrous solvents are shown in Figure S4.

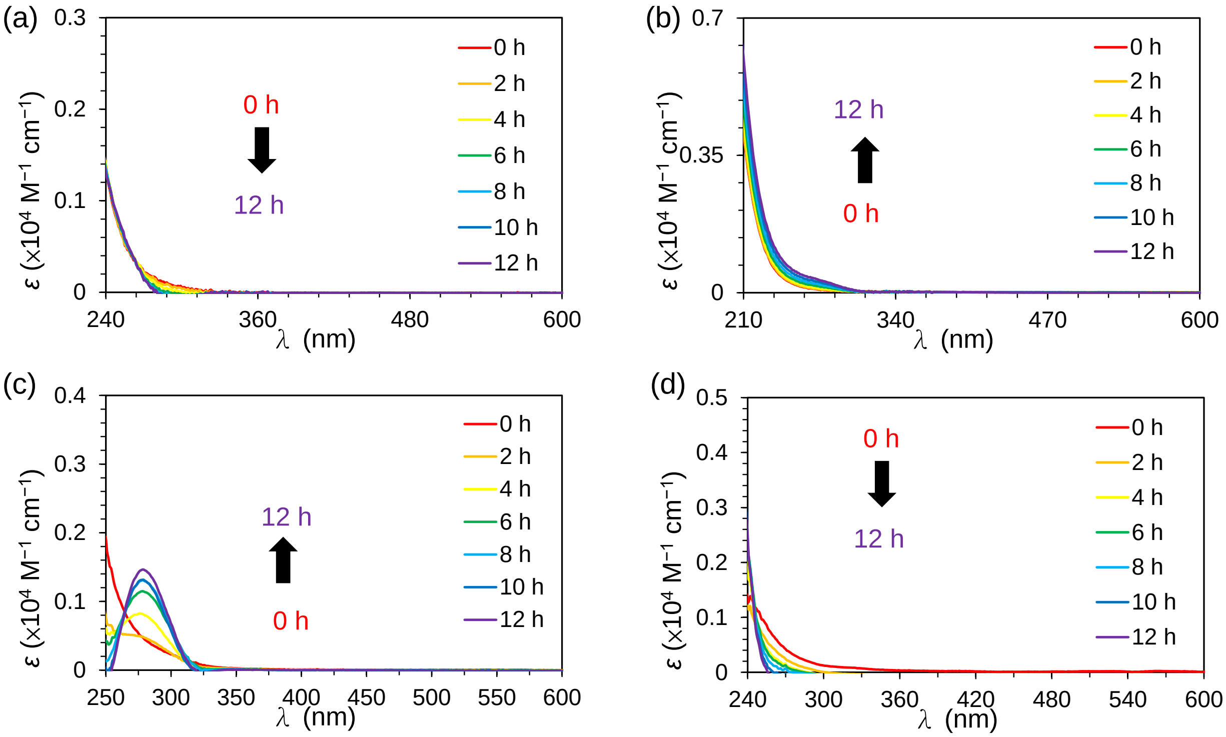

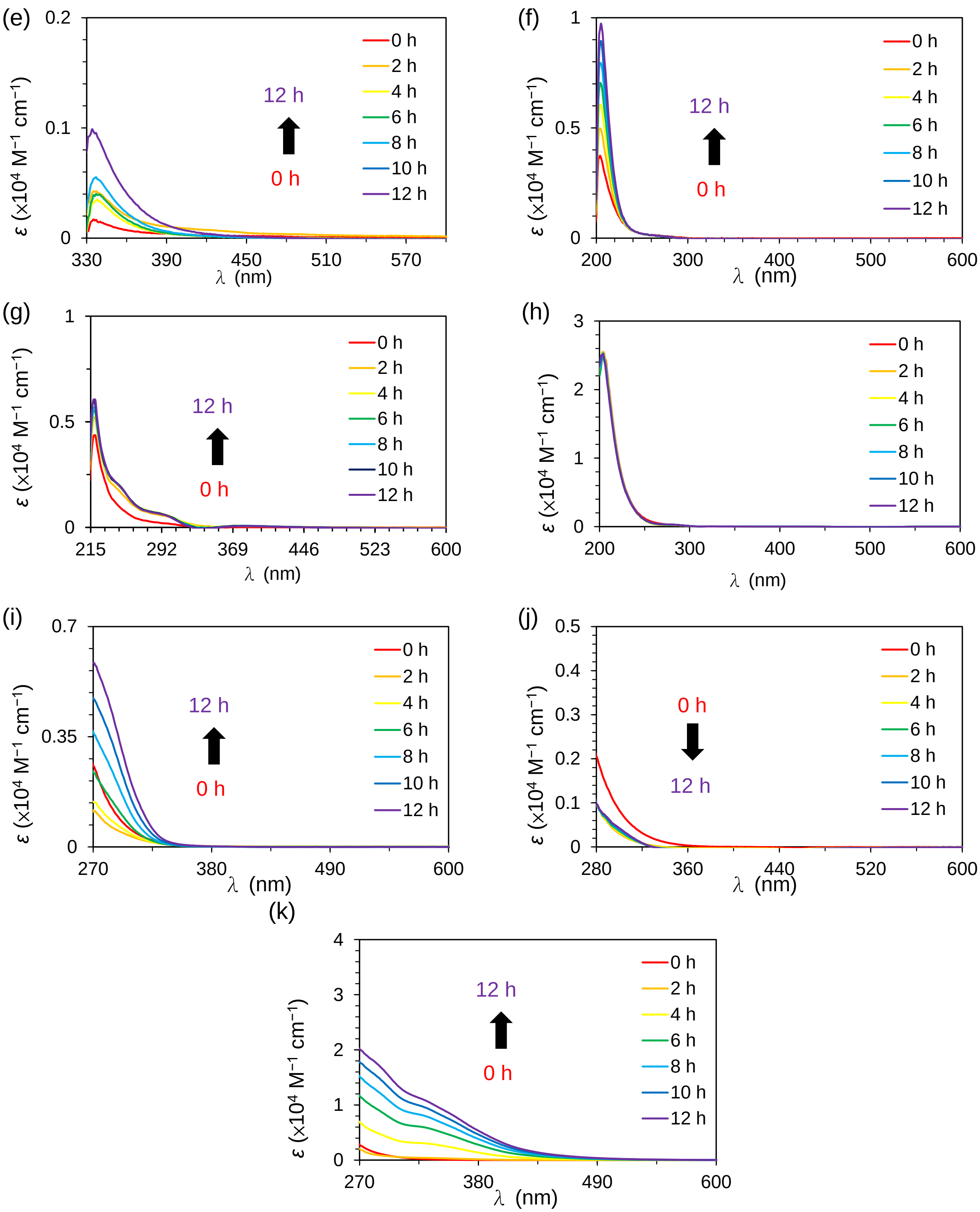

Figure S4. UV-Vis absorption spectra of $[NBu_4]_2[S_2O_8]$ in (a) DCM, (b) IPA, (c) THF, (d) Dioxane, (e) Acetone, (f) MeOH, (g) EtOH, (h) MeCN, (i) DMF, (j) DMA and (k) DMSO.

4. Electrochemical Experiments

4a. General Considerations

All electrochemical experiments are performed using the PalmSens4 potentiostat interfaced to a computer with PSTrace software, employing a three-electrode setup. The three-electrode setup for all cyclic voltammogram (CV) and differential pulse voltammogram (DPV) included a platinum (Pt) working electrode (3.0 mm diameter, WE), a platinum (Pt) wire counter electrode (CE), and a non-aqueous reference electrode (RE) with 0.01 M $AgNO_3$/0.1 M $[NBu_4][PF_6]$. The supporting electrolyte for electrochemical experiments was 0.1 M tetrabutylammonium hexafluorophosphate ($[NBu_4][PF_6]$). The redox potentials of oxidants were initially referenced to the ferrocenium/ferrocene redox couple ($Fc^{+/0}$). For CV studies, all scan rates presented in this work are 300 mV s^{-1} unless otherwise noted. All DPVs were conducted with a pulse amplitude of 50 mV, a pulse period of 0.3 s, an increment of 10 mV, and a scan rate of 10 mV s^{-1}. CVs and DPVs were plotted using the IUPAC convention.

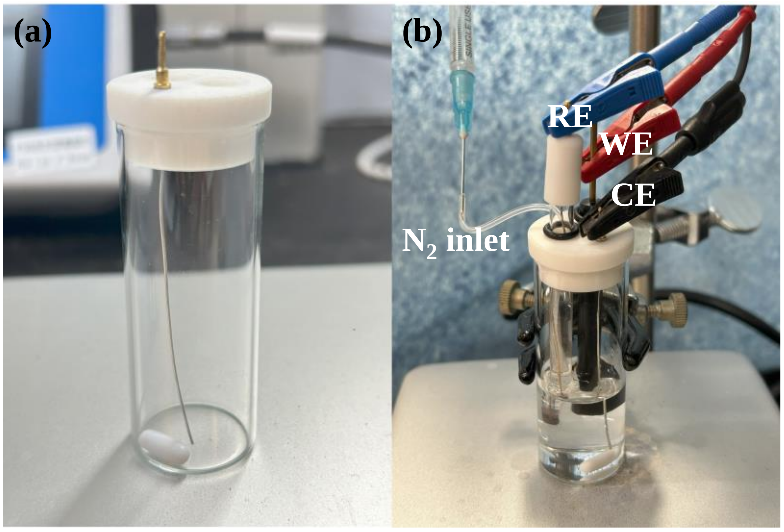

Figure S5. (a) Glass cell vial for voltammetry. (b) A picture of the setup used in electrochemical experiments.

Table S2. Formal potentials (V) for the $Fc^{+/0}$ couple versus SCE in the selected electrolyte.[6]

Solvents	$[NBu_4][PF_6]$	$[NEt_4][PF_6]$	$[NBu_4][ClO_4]$
MeCN	0.40	0.38	0.38
DMSO	–	0.43	0.45
DMF	0.45	0.46	0.47
DCM	0.46	–	0.48
Acetone	0.48	0.46	0.50
THF	0.56	–	0.53
H_2O (0.1 M NaF)	0.16	–	–
MeCN (0.2 M $Li[ClO_4]$)	0.31	–	–

^{a} Supporting electrolyte concentration, 0.1 M.

Table S3. Conversion constants between different reference electrodes (V) in MeCN at 25°C.[7]

	$Fc^{+/0}$	NHE	SCE	SSCE	SHE
$Fc^{+/0}$	0	+0.630	+0.380	+0.384	+0.624

NHE	–0.630	0	–0.250	–0.246	–0.600
SCE	–0.380	+0.250	0	+0.400	+0.244
SSCE	–0.384	+0.246	–0.400	0	+0.235
SHE	–0.624	+0.600	–0.244	–0.235	0

Table S4. Oxidation potentials[e] of different oxidants in various organic solvents. WE: Pt disk electrode.

oxidant<br>solvent	**$[NBu_4]_2[Ce(NO_3)_6]$**	**$[NBu_4][IO_4]$**	**$[NBu_4][HSO_5]$**	**$[NBu_4]_2[S_2O_8]$**
DCM	0.49[a]	–[f]	–	–
IPA	–	–	×[d]	–
THF	0.29[a], 0.63[a]	–	–	–
EA	–	–	×	×
Dioxane	–	–	×	–
Acetone	0.41[b], 0.53[a]	–	0.44[b], 0.63[b]	–
MeOH	0.46[a]	–	–	–
EtOH	–	–	–	–
MeCN	0.27[a], 0.58[a]	1.99[c]	1.83[c]	1.99[c]
DMF	0.51[b]	–	–	–
DMA	–	–	–	–
DMSO	0.51[b]	–	–	–

[a]Quasi-reversible redox wave. [b]Irreversible redox wave. [c]Only oxidative peak wave. [d]× indicates that the SO is insoluble in the solvent. [e]All potentials are referenced to $Fc^{+/0}$ in V. [f]The unmeasured oxidation potential may be obscured by the solvent's oxidation current.

4b. Cyclic voltammograms and differential pulse voltammograms of oxidants in different solvents

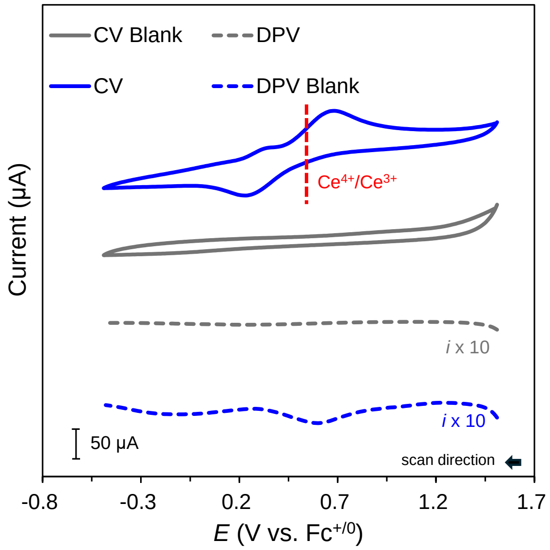

Figure S6. CVs of 1 mM **[NBu$_4$]$_2$[Ce(NO$_3$)$_6$]** in anhydrous DCM. All CVs were recorded under 1 atm N_2 at the scan rate of 300 mV s^{-1}. Gray dotted trace: DPV blank of anhydrous DCM; black dotted trace: DPV of **[NBu$_4$]$_2$[Ce(NO$_3$)$_6$]** in anhydrous DCM; Gray trace: CV blank of anhydrous DCM; black trace: CV of SO. For the signal intensity of DPV, we multiply several times to have a comparable intensity with CV.

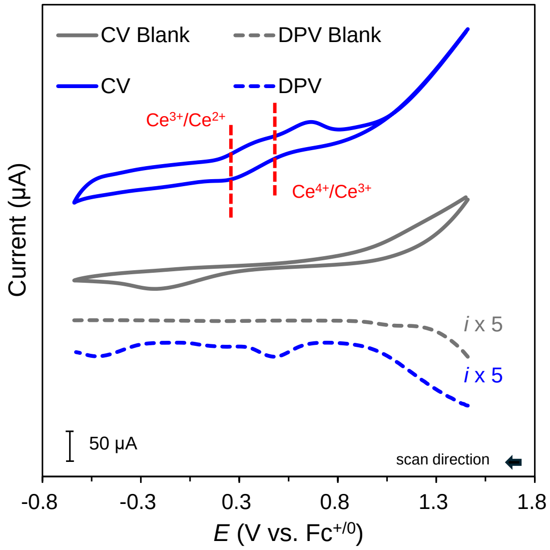

Figure S7. CVs of 1 mM **[NBu$_4$]$_2$[Ce(NO$_3$)$_6$]** in anhydrous THF. All CVs were recorded under 1 atm N_2 at the scan rate of 300 mV s^{-1}. Gray dotted trace: DPV blank of anhydrous THF; black dotted trace: DPV of **[NBu$_4$]$_2$[Ce(NO$_3$)$_6$]** in anhydrous THF; Gray trace: CV blank of anhydrous THF; black trace: CV of SO. For the signal intensity of DPV, we multiply several times to have a comparable intensity with CV.

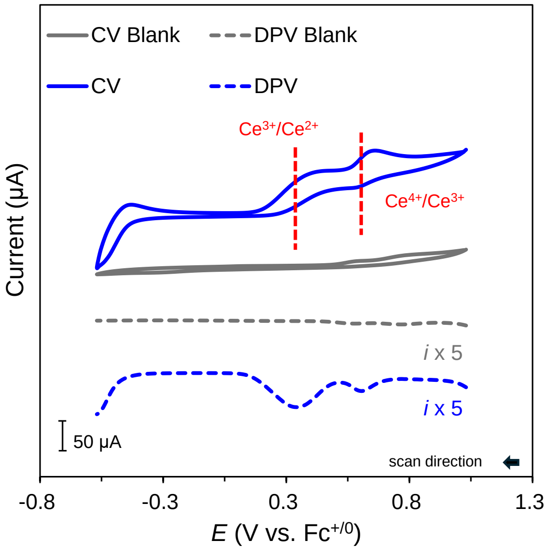

Figure S8. CVs of 1 mM **$[NBu_4]_2[Ce(NO_3)_6]$** in anhydrous acetone. All CVs were recorded under 1 atm N_2 at the scan rate of 300 mV s^{-1}. Gray dotted trace: DPV blank of anhydrous acetone; black dotted trace: DPV of **$[NBu_4]_2[Ce(NO_3)_6]$** in anhydrous acetone; Gray trace: CV blank of anhydrous acetone; black trace: CV of SO. For the signal intensity of DPV, we multiply several times to have a comparable intensity with CV.

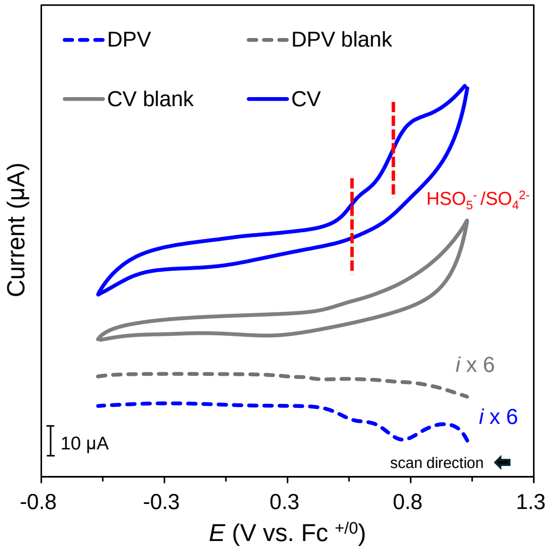

Figure S9. CVs of 1 mM **$[NBu_4][HSO_5]$** in anhydrous acetone. All CVs were recorded under 1 atm N_2 at the scan rate of 300 mV s^{-1}. Gray dotted trace: DPV blank of anhydrous acetone; black dotted trace: DPV of **$[NBu_4][HSO_5]$** in anhydrous acetone; Gray trace: CV blank of anhydrous acetone; black trace: CV of SO. For the signal intensity of DPV, we multiply several times to have a comparable intensity with CV.

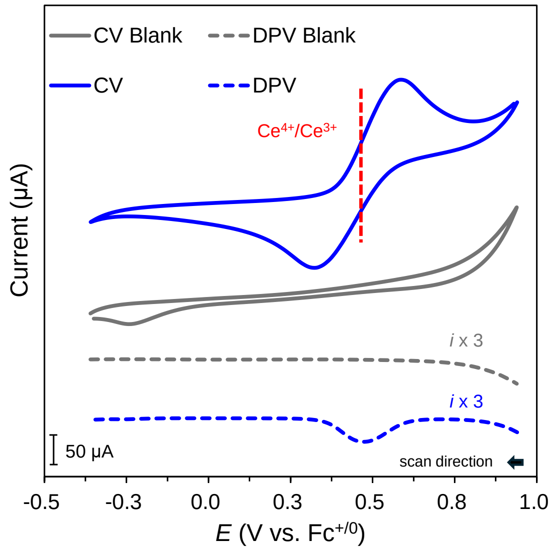

Figure S10. CVs of 1 mM **$[NBu_4]_2[Ce(NO_3)_6]$** in anhydrous MeOH. All CVs were recorded under 1 atm N_2 at the scan rate of 300 mV s^{-1}. Gray dotted trace: DPV blank of anhydrous MeOH; black dotted trace: DPV of **$[NBu_4]_2[Ce(NO_3)_6]$** in anhydrous MeOH; Gray trace: CV blank of anhydrous MeOH; black trace: CV of SO. For the signal intensity of DPV, we multiply several times to have a comparable intensity with CV.

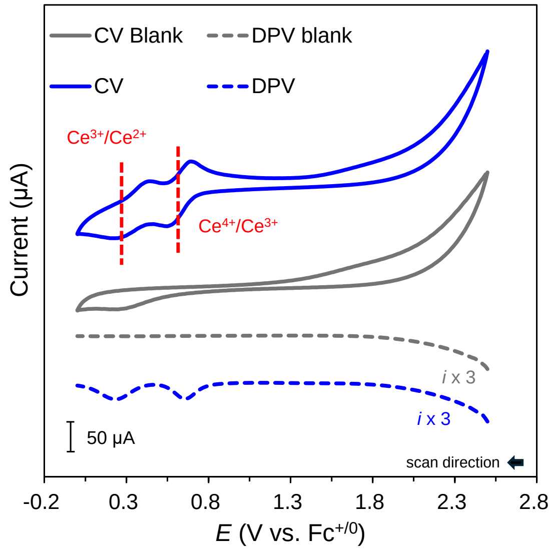

Figure S11. CVs of 1 mM **$[NBu_4]_2[Ce(NO_3)_6]$** in anhydrous MeCN. All CVs were recorded under 1 atm N_2 at the scan rate of 300 mV s^{-1}. Gray dotted trace: DPV blank of anhydrous MeCN; black dotted trace: DPV of **$[NBu_4]_2[Ce(NO_3)_6]$** in anhydrous MeCN; Gray trace: CV blank of anhydrous MeCN; black trace: CV of SO. For the signal intensity of DPV, we multiply several times to have a comparable intensity with CV.

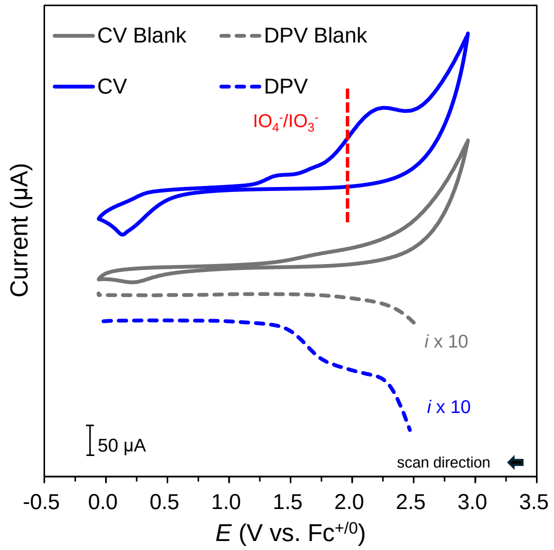

Figure S12. CVs of 1 mM **[NBu$_4$][IO$_4$]** in anhydrous MeCN. All CVs were recorded under 1 atm N_2 at the scan rate of 300 mV s^{-1}. Gray dotted trace: DPV blank of anhydrous MeCN; black dotted trace: DPV of **[NBu$_4$][IO$_4$]** in anhydrous MeCN; Gray trace: CV blank of anhydrous MeCN; black trace: CV of SO. For the signal intensity of DPV, we multiply several times to have a comparable intensity with CV.

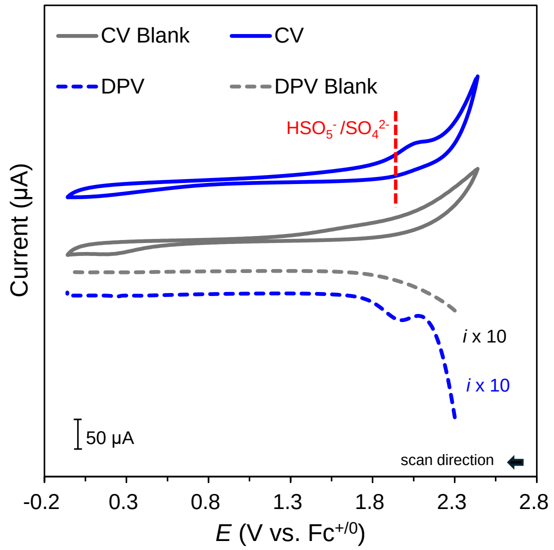

Figure S13. CVs of 1 mM **[NBu$_4$][HSO$_5$]** in anhydrous MeCN. All CVs were recorded under 1 atm N_2 at the scan rate of 300 mV s^{-1}. Gray dotted trace: DPV blank of anhydrous MeCN; black dotted trace: DPV of **[NBu$_4$][HSO$_5$]** in anhydrous MeCN; Gray trace: CV blank of anhydrous MeCN; black trace: CV of SO. For the signal intensity of DPV, we multiply several times to have a comparable intensity with CV.

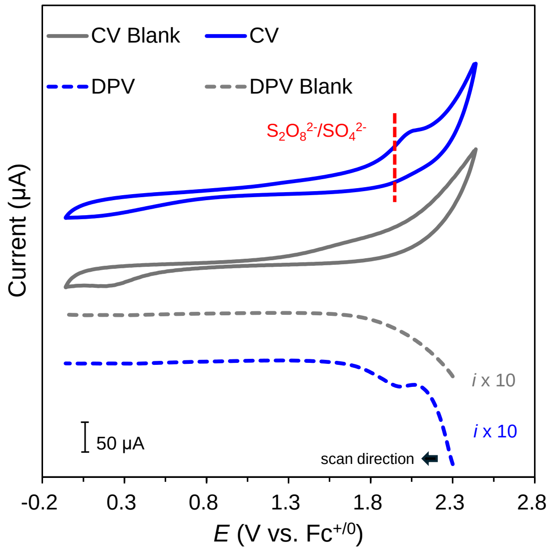

Figure S14. CVs of 1 mM **[NBu$_4$]$_2$[S$_2$O$_8$]** in anhydrous MeCN. All CVs were recorded under 1 atm N_2 at the scan rate of 300 mV s^{-1}. Gray dotted trace: DPV blank of anhydrous MeCN; black dotted trace: DPV of **[NBu$_4$]$_2$[S$_2$O$_8$]** in anhydrous MeCN; Gray trace: CV blank of anhydrous MeCN; black trace: CV of SO. For the signal intensity of DPV, we multiply several times to have a comparable intensity with CV.

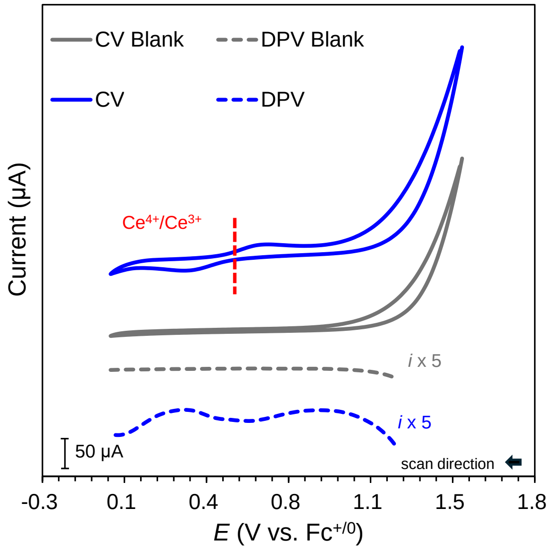

Figure S15. CVs of 1 mM **[NBu$_4$]$_2$[Ce(NO$_3$)$_6$]** in anhydrous DMF. All CVs were recorded under 1 atm N_2 at the scan rate of 300 mV s^{-1}. Gray dotted trace: DPV blank of anhydrous DMF; black dotted trace: DPV of **[NBu$_4$]$_2$[Ce(NO$_3$)$_6$]** in anhydrous DMF; Gray trace: CV blank of anhydrous DMF; black trace: CV of SO. For the signal intensity of DPV, we multiply several times to have a comparable intensity with CV.

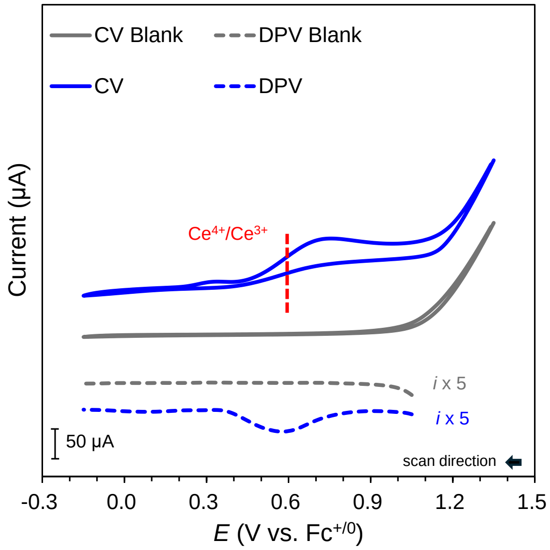

Figure S16. CVs of 1 mM **[NBu_4]$_2$[Ce(NO_3)$_6$]** in anhydrous DMSO. All CVs were recorded under 1 atm N_2 at the scan rate of 300 mV s^{-1}. Gray dotted trace: DPV blank of anhydrous DMSO; black dotted trace: DPV of **[NBu_4]$_2$[Ce(NO_3)$_6$]** in anhydrous DMSO; Gray trace: CV blank of anhydrous DMSO; black trace: CV of SO. For the signal intensity of DPV, we multiply several times to have a comparable intensity with CV.

Table S5. Formal Potentials (V) for the $Fc^{+/0}$ couple versus Ag/$AgNO_3$ or Ag/$AgPF_6$ in [NBu_4][PF_6].

electrolyte / solvent	Ag/$AgNO_3$[a]	Ag/$AgPF_6$[b]
THF	–	–0.32
Acetone	–0.12	–0.20
MeOH	–0.16	–0.16
MeCN	0.06	0.07
DMF	0.01	–0.02
DMA	0.06	0.06
DMSO	0.15	0.13

[a]All potentials are referenced to Ag/$AgNO_3$ in V. [b]All potentials are referenced to Ag/$AgPF_6$ in V.

5. Estimation of the Thermodynamic Reduction Potential of O_2/H_2O at Non-standard State (aqueous)

5a. General Considerations

The H^+/H_2 potential in organic media can be determined using a recently reported protocol through open-circuit potential (OCP) measurements at a Pt electrode.[8] (Figure S17.) The H^+/H_2 potentials were measured for various solvents containing H_2O (0.01, 0.1, 1, and 10 M) under 1 atm H_2 (local atmospheric pressure of 756 mm Hg, correction to 1 atm < 1 mV). All solutions contained 0.1 M $[NBu_4][PF_6]$ or 0.1 M $[NBu_4][BF_4]$ supporting electrolyte. Dioxane is employed with an electrolyte concentration of 0.3 M tetrabutylammonium tetrafluoroborate ($[NBu_4][BF_4]$). A stable OCP was observed, and this potential was corrected to be versus $Ag/AgNO_3$ or Ag/AgCl (KCl, 3 M).

The pre-treatment method for Pt wire was referenced from the literature: *Inorg. Chem.* **2013**, *52*, 3823−3835. The platinum wire electrodes used for OCP (BASI; 99.95%, 0.5 mm diam, ~60 mm length) were prepared by rinsing with acetone, drying in air, immersing in freshly prepared aqua regia for 30 min, then rinsing with flowing deionized water for 5 min. Each wire was then clamped into a stainless steel hemostat and heated to a uniform yellow-orange glow in a flame. Once the entire wire was at the maximum temperature attainable (judged by color), the flame was extinguished, and the wire was allowed to cool in the hydrogen-nitrogen (5% hydrogen/nitrogen) stream until it no longer glowed. At this point, the wire was placed in a screw-cap storage tube maintained under a positive flow of stream.

After each wire was treated, the tube was closed and taken into the glovebox. Each wire was useable for at least several measurements, sometimes affording consistent data for up to a day. Unused electrodes remained active for several days if stored in the glovebox. Excessive and increasing signal noise, leading ultimately to a substantial instability of the OCP, was generally taken to indicate deterioration of the electrode response, and the electrode was replaced. Substitution of one electrode with another afforded the same OCP value within 3 mV. The same set of four platinum wire electrodes was reconditioned as described above and reused numerous times with no effect on the OCP measurements.

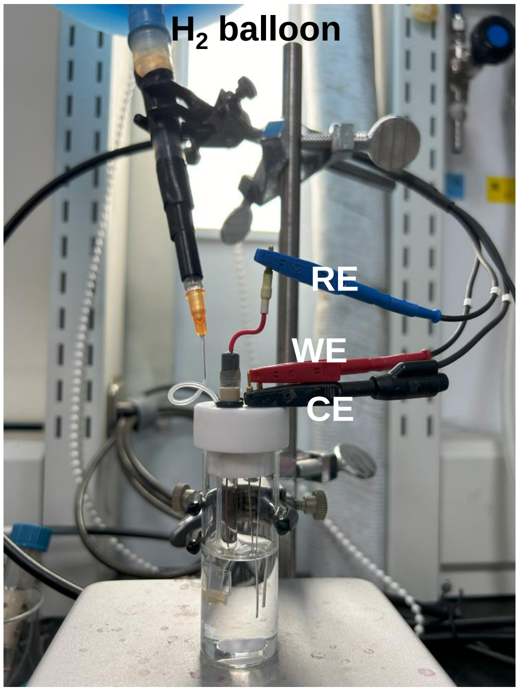

Figure S17. Schematic of the four-electrode cell configuration used for OCP measurements. The reduction potential of H^+/H_2 was measured for a solution containing $[NBu_4][PF_6]$ (0.1 M), H_2O (0.01, 0.1, 1, and 10 M), under 1 atm H_2.

5b. The OCP measurement of H^+/H_2 redox couples (E_{H^+/H_2}) in various organic solvents

Table S6. OCP of H^+/H_2 redox couples[a] (E_{H^+/H_2}) vs. $Fc^{+/0}$ with various organic solvents.

Solvent/H_2O conc.	**0 M H_2O**
DCM	–0.960
IPA	–0.696
THF	–0.796
EA[b]	–0.471
Dioxane	–0.731
Acetone	–0.627
MeOH	–0.890
EtOH	–0.775
MeCN	–0.295
DMF	–0.569
DMA	–1.041
DMSO[a]	–1.008

[a]All potentials averaged from triplicate measurements. [b]All potentials are referenced to $Fc^{*+/0}$ in V.

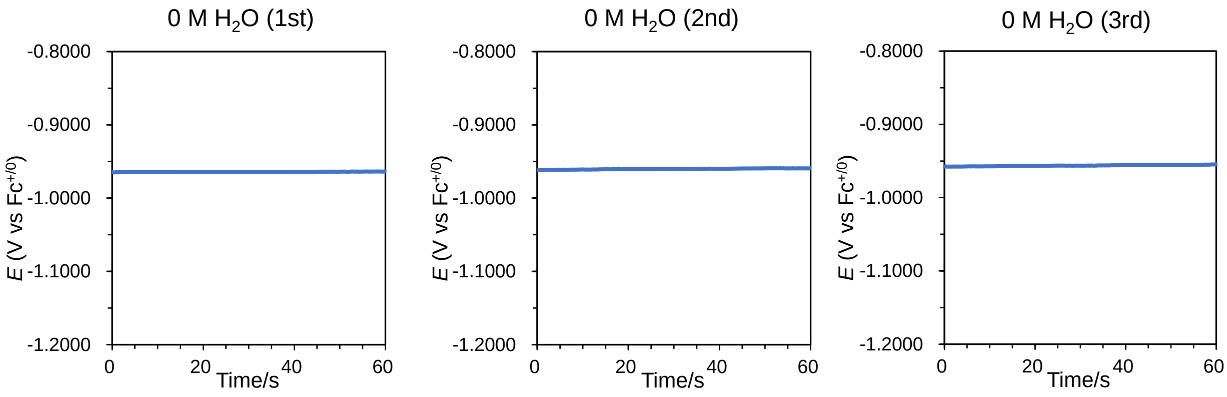

Figure S18. The blue traces show the average OCP of E_{H^+/H_2} in anhydrous DCM under 1 atm H_2. Three OCP experiments in anhydrous DCM under 1 atm $H_{2,}$ E_{H^+/H_2}= –0.960 V.

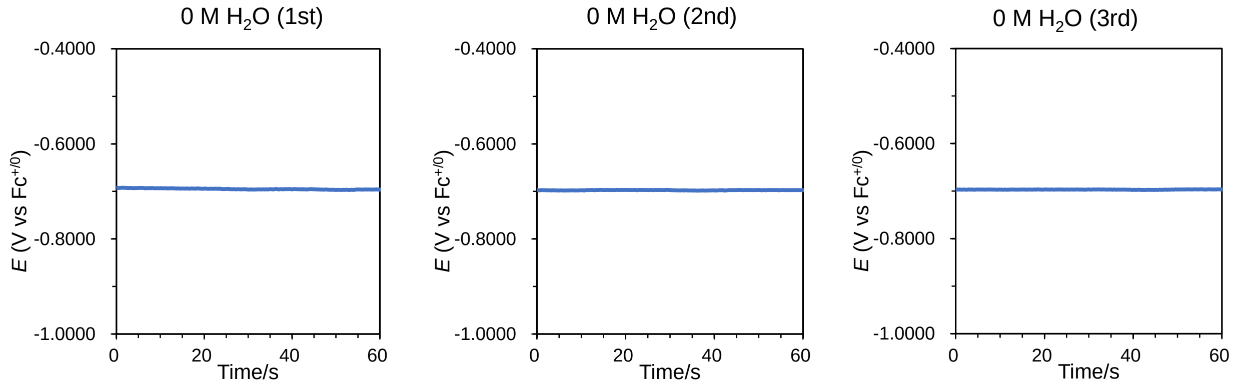

Figure S19. The blue traces show the average OCP of E_{H^+/H_2} in anhydrous IPA under 1 atm H_2. Three OCP experiments in anhydrous IPA under 1 atm H_2, E_{H^+/H_2}= –0.696 V.

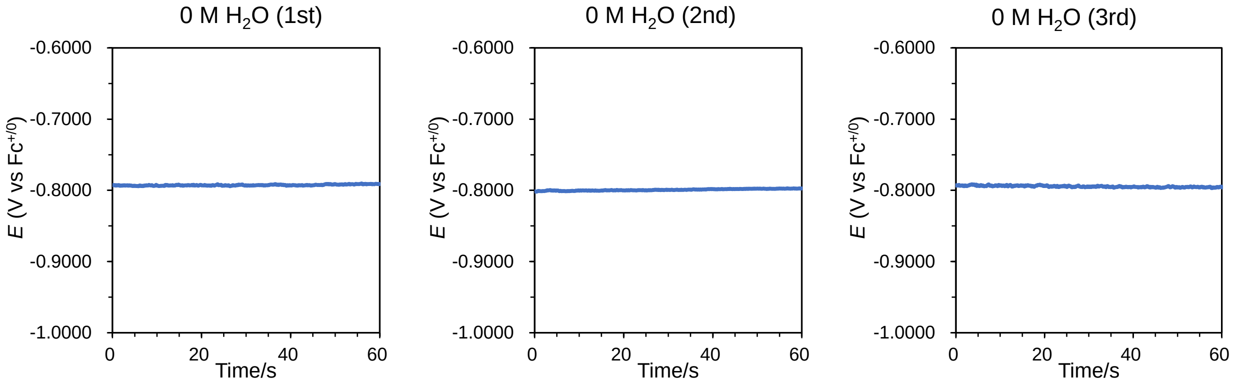

Figure S20. The blue traces show the average OCP of E_{H^+/H_2} in anhydrous THF under 1 atm H_2. Three OCP experiments in anhydrous THF under 1 atm H_2, E_{H^+/H_2}= –0.796 V.

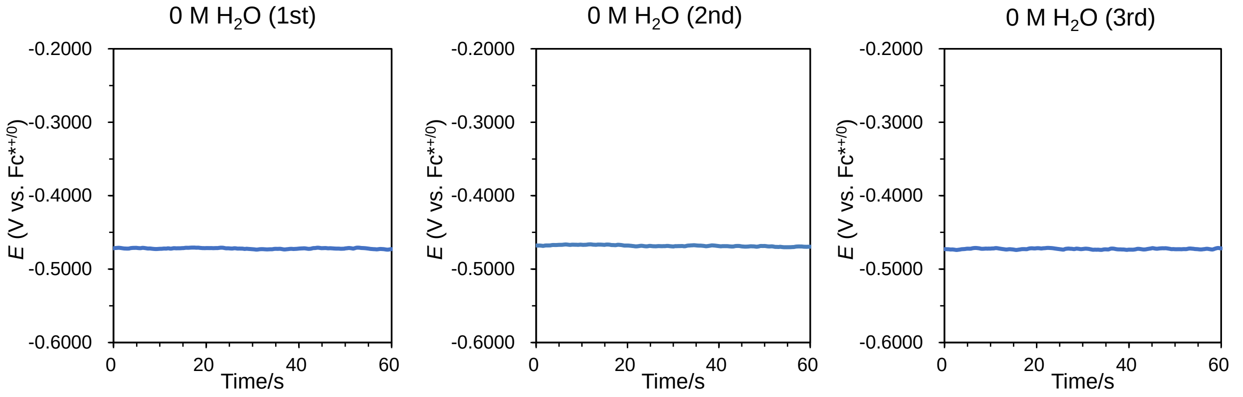

Figure S21. The blue traces show the average OCP of E_{H^+/H_2} in anhydrous EA under 1 atm H_2. Three OCP experiments in anhydrous EA under 1 atm H_2, E_{H^+/H_2}= –0.471 V.

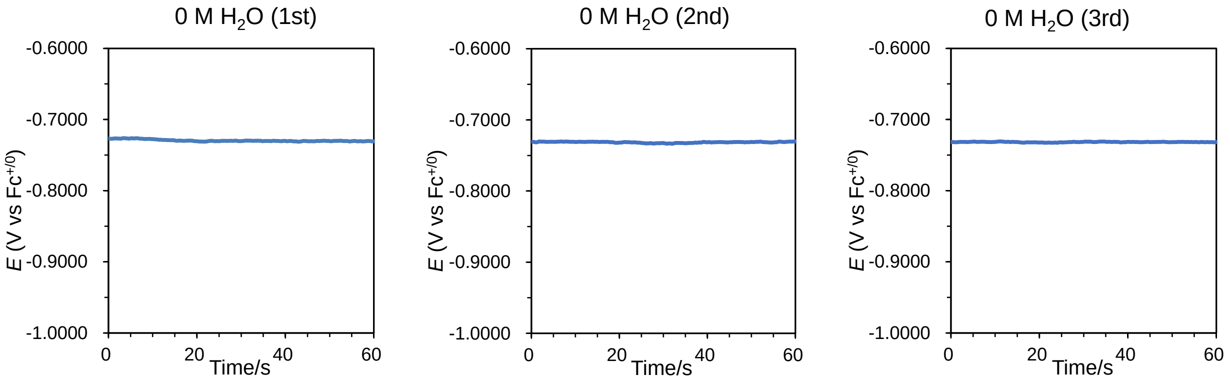

Figure S22. The blue traces show the average OCP of E_{H^+/H_2} in anhydrous dioxane under 1 atm H_2. Three OCP experiments in anhydrous dioxane under 1 atm H, E_{H^+/H_2}= –0.731 V.

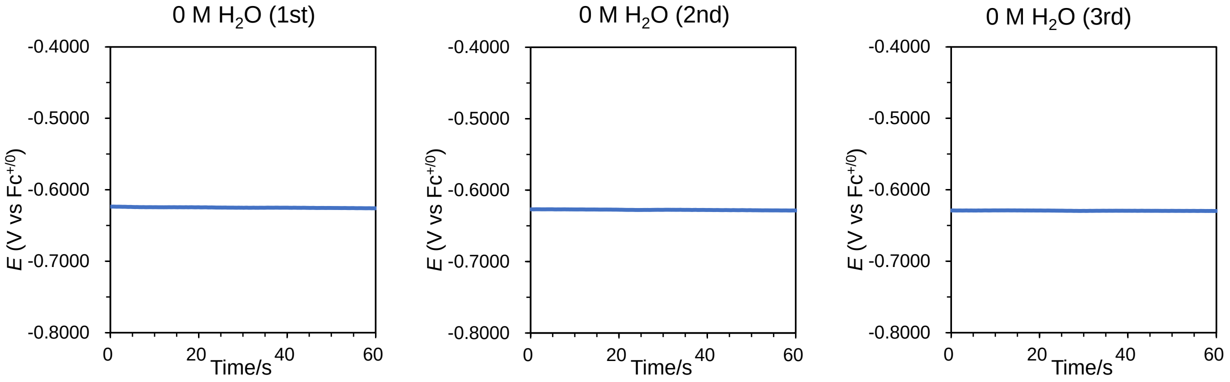

Figure S23. The blue traces show the average OCP of E_{H^+/H_2} in anhydrous acetone under 1 atm H_2. Three OCP experiments in anhydrous acetone under 1 atm H_2, E_{H^+/H_2}= –0.627 V.

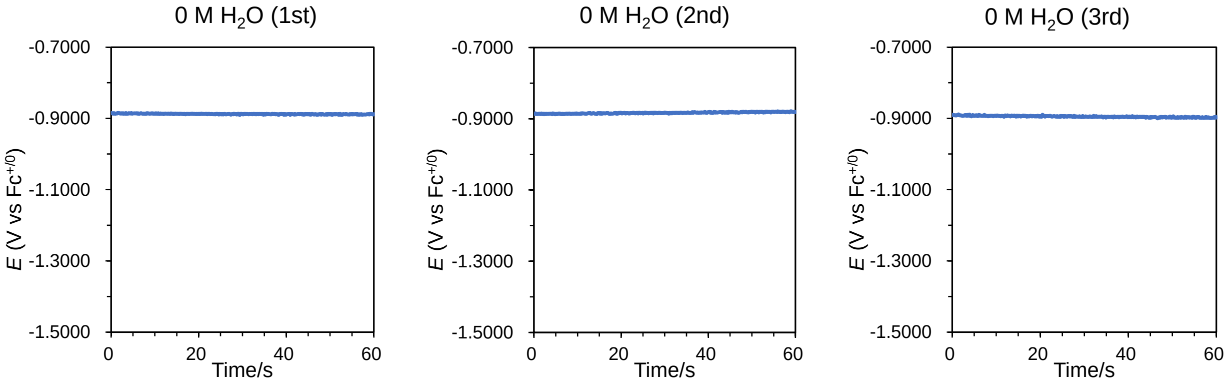

Figure S24. The blue traces show the average OCP of E_{H^+/H_2} in anhydrous MeOH under 1 atm H_2. Three OCP experiments in anhydrous MeOH under 1 atm H_2, E_{H^+/H_2}= –0.890 V.

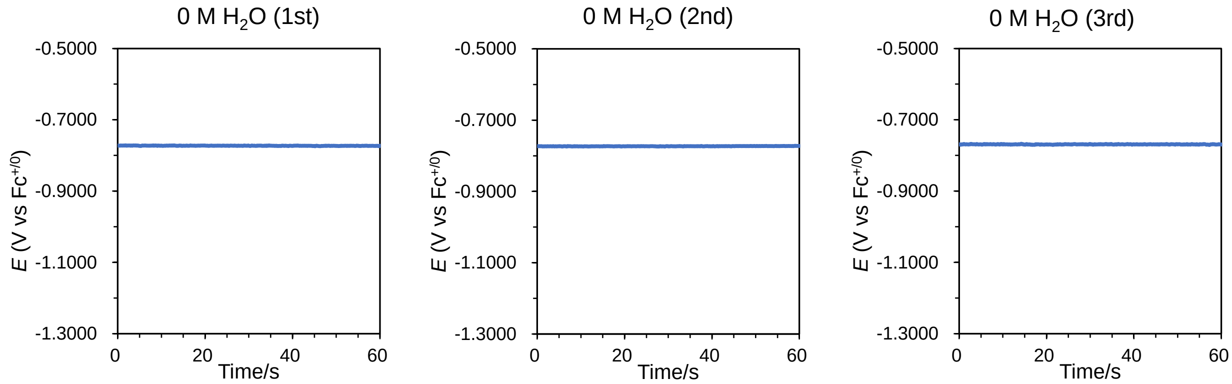

Figure S25. The blue traces show the average OCP of E_{H^+/H_2} in anhydrous EtOH under 1 atm H_2. Three OCP experiments in anhydrous EtOH under 1 atm H_2, E_{H^+/H_2}= –0.775 V.

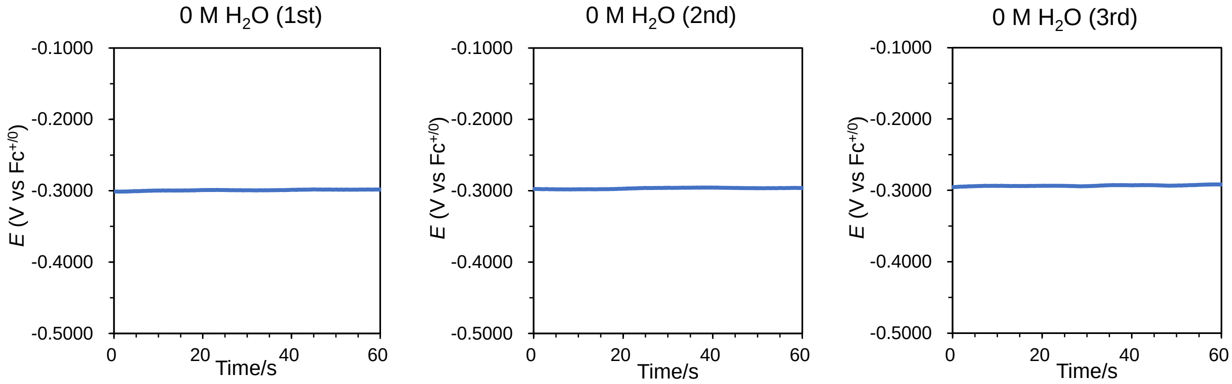

Figure S26. The blue traces show the average OCP of E_{H^+/H_2} in anhydrous MeCN under 1 atm H_2. Three OCP experiments in anhydrous MeCN under 1 atm H_2, E_{H^+/H_2}= –0.295 V.

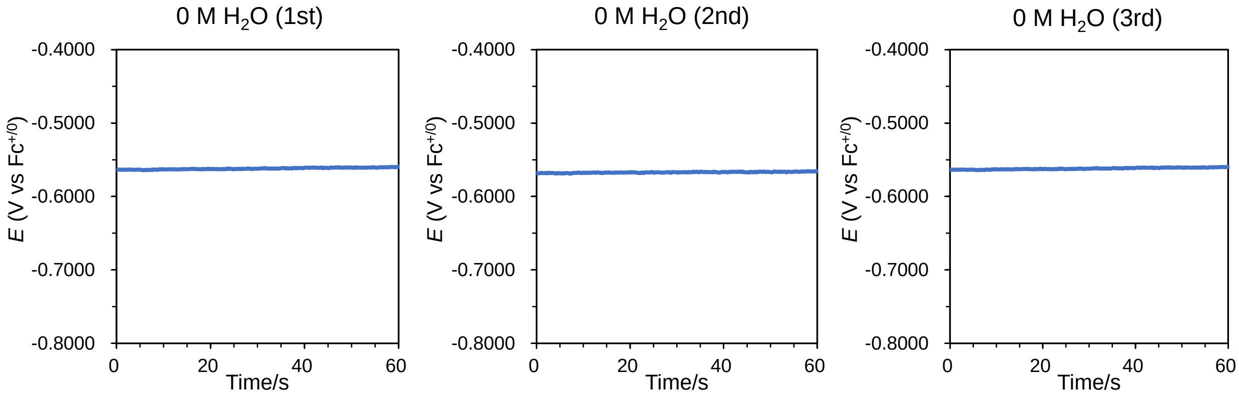

Figure S27. The blue traces show the average OCP of E_{H^+/H_2} in anhydrous DMF under 1 atm H_2. Three OCP experiments in anhydrous DMF under 1 atm H_2, E_{H^+/H_2}= –0.569 V.

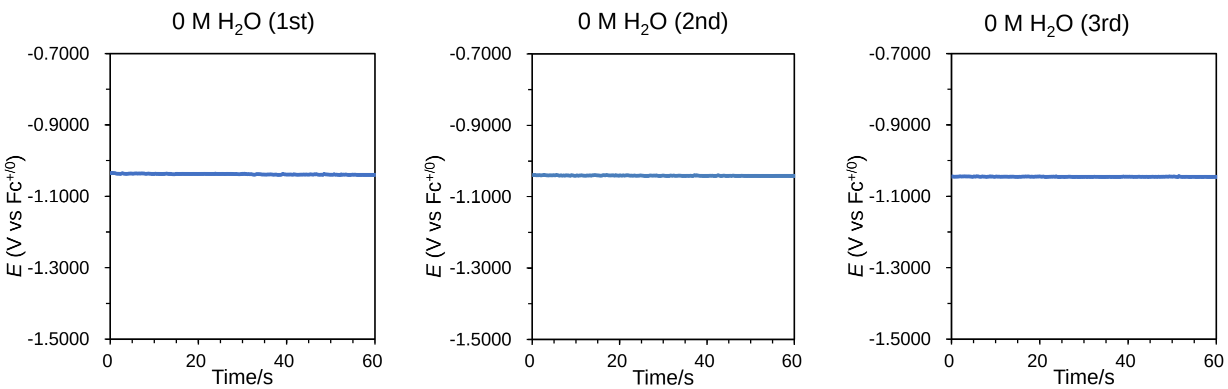

Figure S28. The blue traces show the average OCP of E_{H^+/H_2} in anhydrous DMA under 1 atm H_2. Three OCP experiments in anhydrous DMA under 1 atm H_2, E_{H^+/H_2}= −1.041 V.

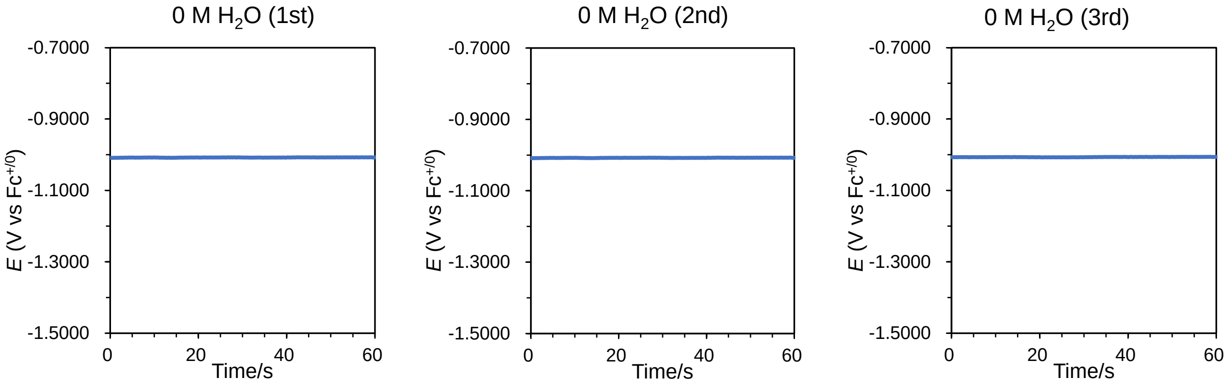

Figure S29. The blue traces show the average OCP of E_{H^+/H_2} in anhydrous DMSO under 1 atm H_2. Three OCP experiments in anhydrous DMSO under 1 atm H_2, E_{H^+/H_2}= −1.008 V.

5c. The OCP measurement of H^+/H_2 redox couples (E_{H^+/H_2}) under neutral conditions (H_2O: 0.01–10 M)

Table S7. OCP of H^+/H_2 redox couples[a] (E_{H^+/H_2}) vs. Ag/AgCl with various concentrations of [H_2O] (0.01–10 M).

Solvent/H_2O conc.	0.01 M	0.1 M	**1 M**	10 M
DCM		Water insoluble		
IPA	−0.293	−0.295	**−0.297**	−0.333
THF	−0.239	−0.259	**−0.309**	−0.392
EA		Water insoluble		
Dioxane	−0.314	−0.368	**−0.406**	−0.434
Acetone	−0.380	−0.401	**−0.427**	−0.517
MeOH	−0.442	−0.463	**−0.457**	−0.483
EtOH	−0.384	−0.378	**−0.374**	−0.433
MeCN	−0.041	−0.066	**−0.073**	−0.160

DMF	–0.438	–0.438	**–0.444**	–0.496
DMA	–0.500	–0.496	**–0.501**	–0.540
DMSO[a]	–1.012	–1.016	**–1.014**	–1.013

[a]All potentials are referenced to Ag/AgCl (KCl, 3 M) in V, except for DMSO, which is referenced to $Fc^{+/0}$ in V. [b]All potentials averaged from triplicate measurements.

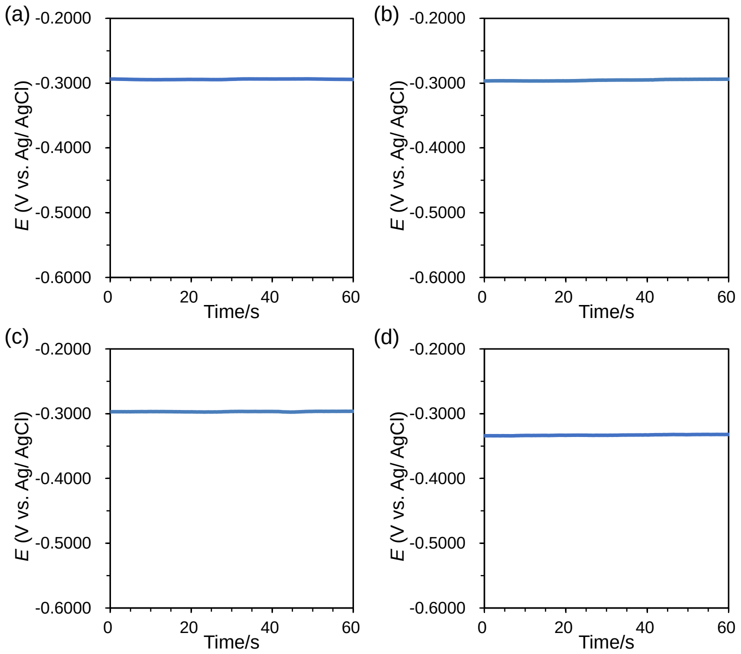

Figure S30. The blue traces show OCP of E_{H^+/H_2} in IPA solutions under 1 atm H_2. (a) 0.01 M H_2O (0.018%, v/v); (b) 0.1 M H_2O (0.18%, v/v); (c) 1 M H_2O (1.8%, v/v) and; (d) 10 M H_2O (18%, v/v) Supporting electrolyte: 0.1 M $[NBu_4][BF_4]$.

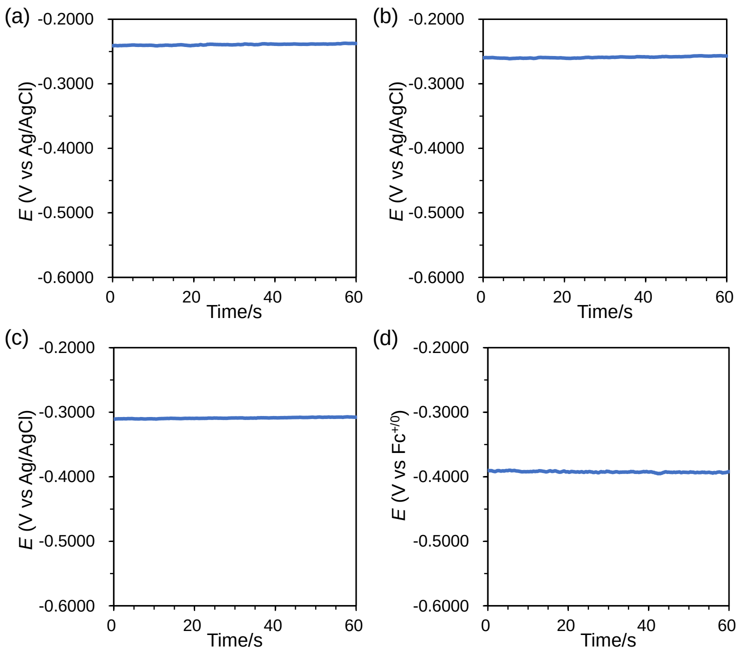

Figure S31. The blue traces show OCP of E_{H^+/H_2} in THF solutions under 1 atm H_2. (a) 0.01 M H_2O (0.018%, v/v); (b) 0.1 M H_2O (0.18%, v/v); (c) 1 M H_2O (1.8%, v/v) and; (d) 10 M H_2O (18%, v/v) Supporting electrolyte: 0.1 M $[NBu_4][PF_6]$.

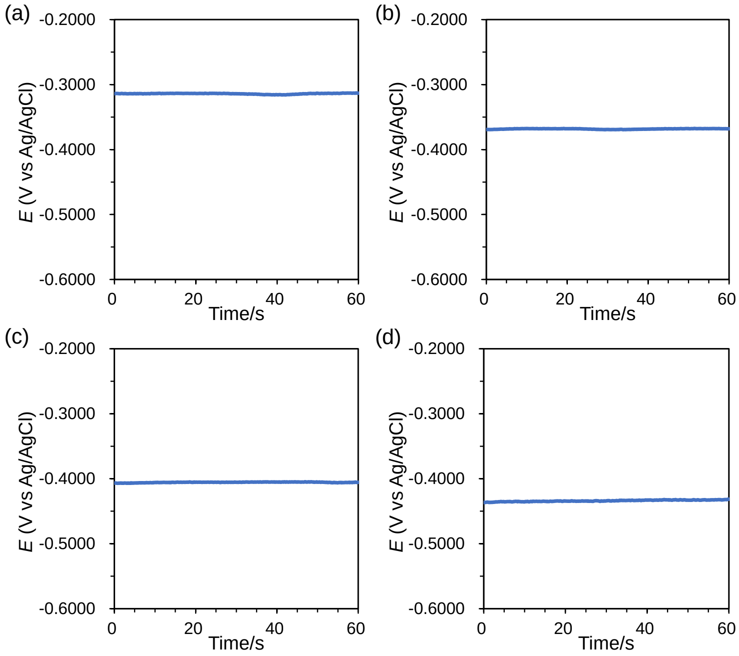

Figure S32. The blue traces show the OCP of E_{H^+/H_2} in dioxane solutions under 1 atm H_2. (a) 0.01 M H_2O (0.018%, v/v); (b) 0.1 M H_2O (0.18%, v/v); (c) 1 M H_2O (1.8%, v/v) and; (d) 10 M H_2O (18%, v/v) Supporting electrolyte: 0.1 M $[NBu_4][BF_4]$.

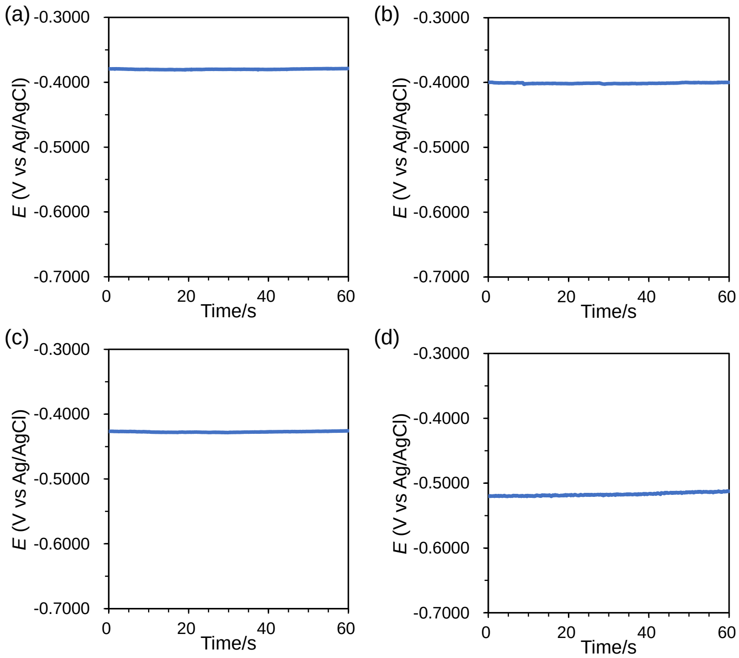

Figure S33. The blue traces show OCP of E_{H^+/H_2} in acetone solutions under 1 atm H_2. (a) 0.01 M H_2O (0.018%, v/v); (b) 0.1 M H_2O (0.18%, v/v); (c) 1 M H_2O (1.8%, v/v) and; (d) 10 M H_2O (18%, v/v) Supporting electrolyte: 0.1 M [NBu_4][PF_6].

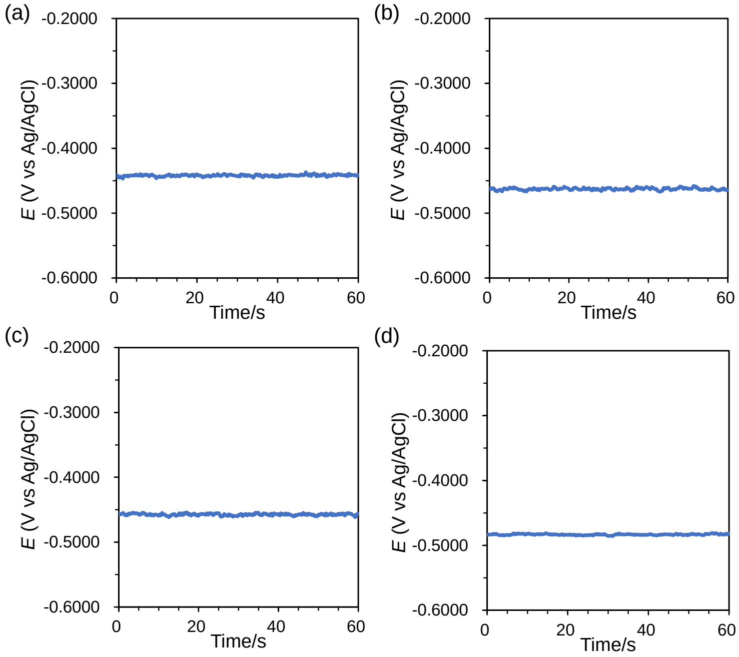

Figure S34. The blue traces show OCP of E_{H^+/H_2} in MeOH solutions under 1 atm H_2. (a) 0.01 M H_2O (0.018%, v/v); (b) 0.1 M H_2O (0.18%, v/v); (c) 1 M H_2O (1.8%, v/v) and; (d) 10 M H_2O (18%, v/v) Supporting electrolyte: 0.1 M [NBu_4][PF_6].

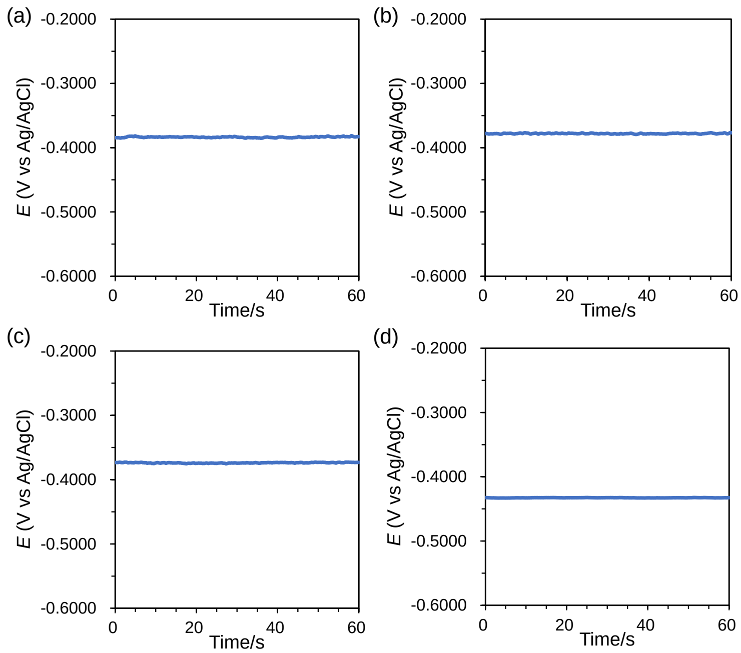

Figure S35. The blue traces show OCP of E_{H^+/H_2} in EtOH solutions under 1 atm H_2. (a) 0.01 M H_2O (0.018%, v/v); (b) 0.1 M H_2O (0.18%, v/v); (c) 1 M H_2O (1.8%, v/v) and; (d) 10 M H_2O (18%, v/v) Supporting electrolyte: 0.1 M $[NBu_4][BF_4]$.

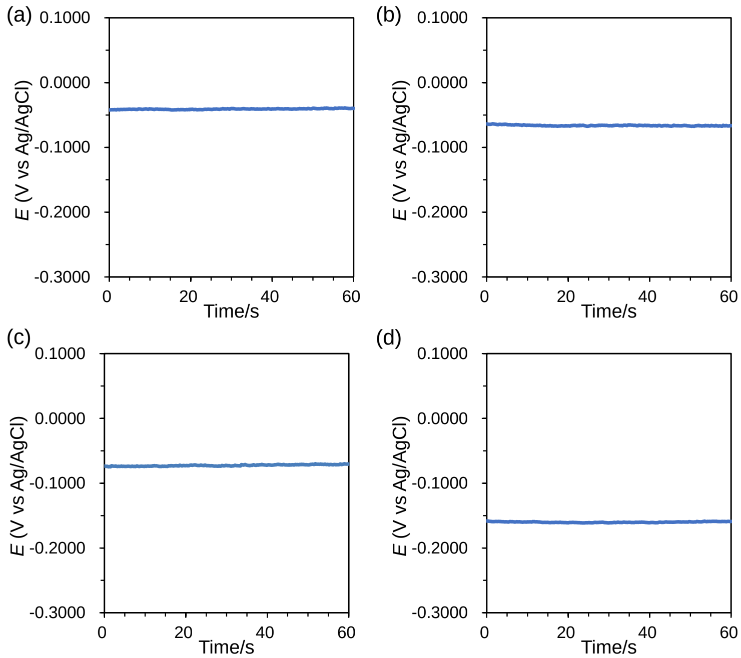

Figure S36. The blue traces show the OCP of E_{H^+/H_2} in MeCN solutions under 1 atm H_2. (a) 0.01 M H_2O (0.018%, v/v); (b) 0.1 M H_2O (0.18%, v/v); (c) 1 M H_2O (1.8%, v/v) and; (d) 10 M H_2O (18%, v/v) Supporting electrolyte: 0.1 M $[NBu_4][PF_6]$.

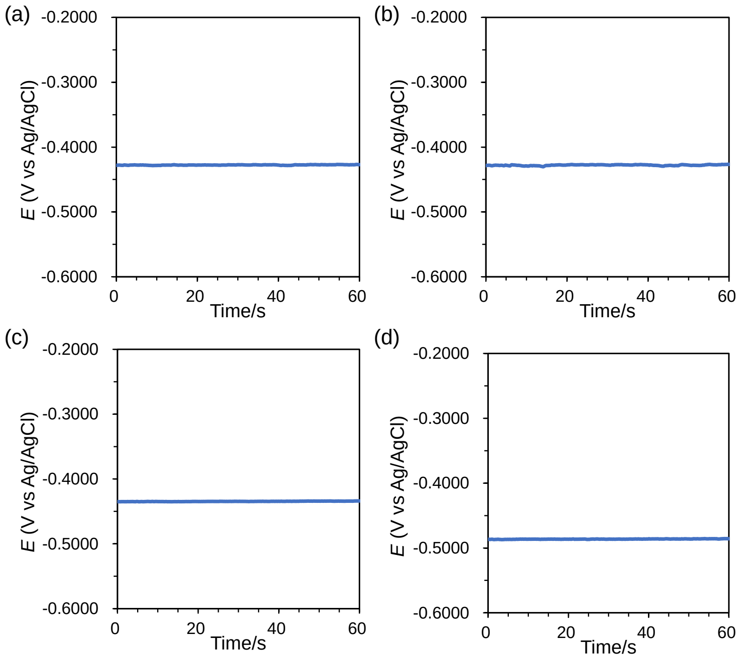

Figure S37. The blue traces show OCP of E_{H^+/H_2} in DMF solutions under 1 atm H_2. (a) 0.01 M H_2O (0.018%, v/v); (b) 0.1 M H_2O (0.18%, v/v); (c) 1 M H_2O (1.8%, v/v) and; (d) 10 M H_2O (18%, v/v) Supporting electrolyte: 0.1 M [NBu_4][PF_6].

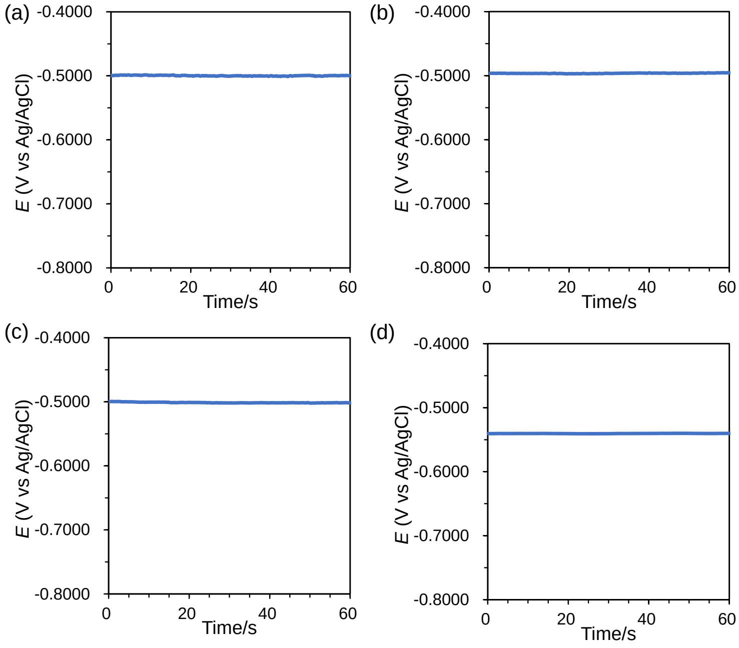

Figure S38. The blue traces show OCP of E_{H^+/H_2} in DMA solutions under 1 atm H_2. (a) 0.01 M H_2O (0.018%, v/v); (b) 0.1 M H_2O (0.18%, v/v); (c) 1 M H_2O (1.8%, v/v) and; (d) 10 M H_2O (18%, v/v) Supporting electrolyte: 0.1 M [NBu_4][PF_6].

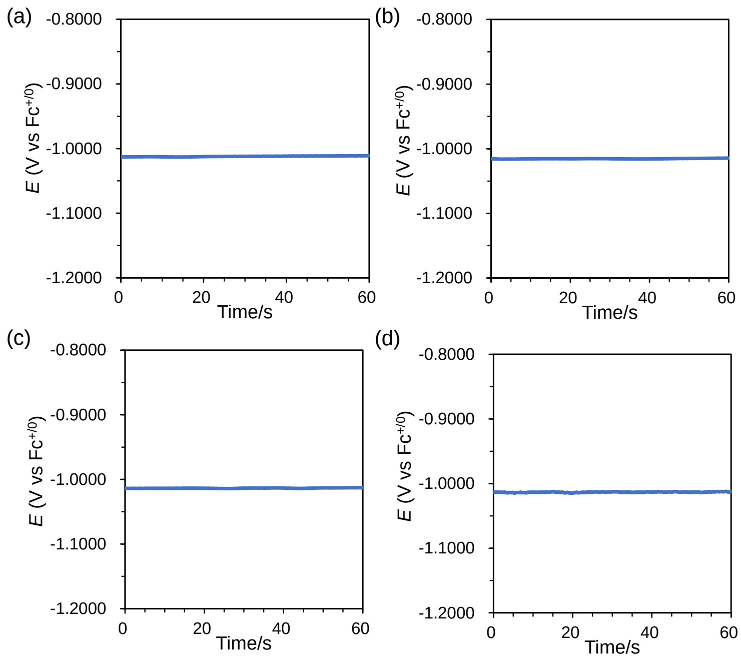

Figure S39. The blue traces show the OCP of E_{H^+/H_2} in DMSO solutions under 1 atm H_2. (a) 0.01 M H_2O (0.018%, v/v); (b) 0.1 M H_2O (0.18%, v/v); (c) 1 M H_2O (1.8%, v/v) and; (d) 10 M H_2O (18%, v/v) Supporting electrolyte: 0.1 M [NBu_4][PF_6].

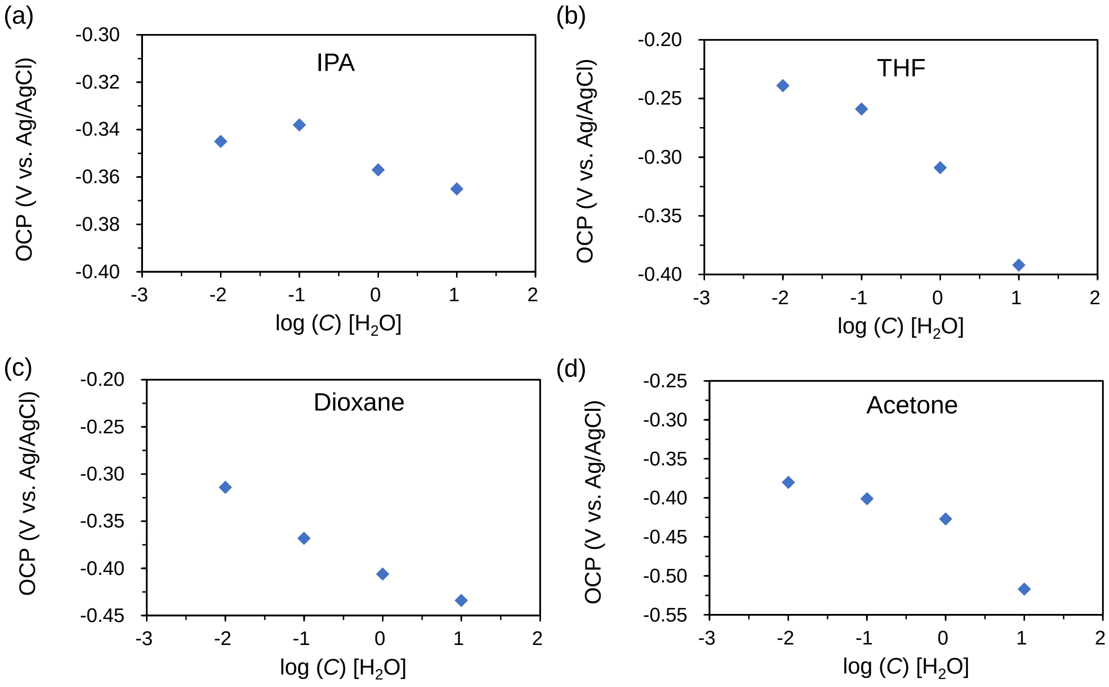

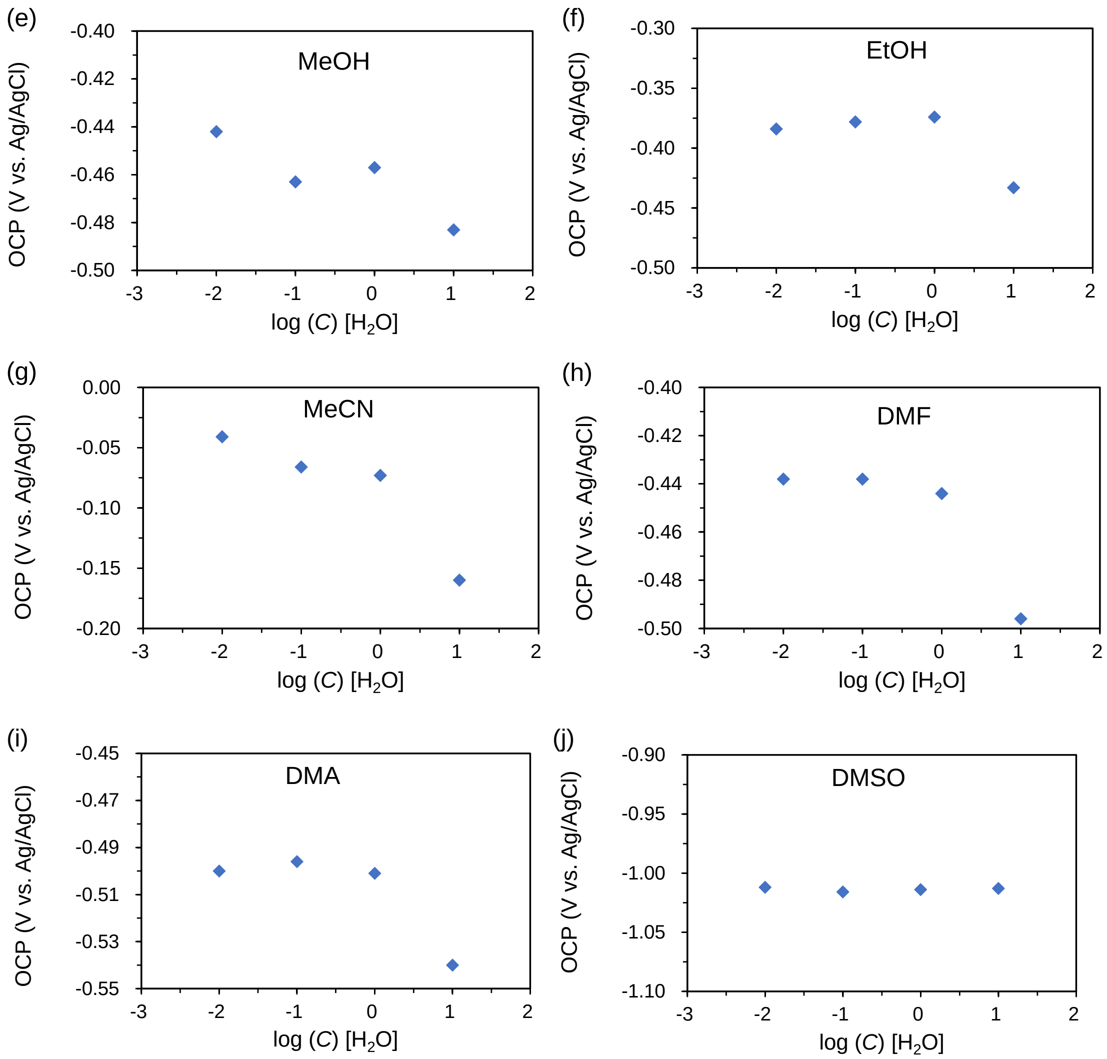

Figure S40. OCP measurements vs. log(C) [H_2O] of organic solvents.

5d. Gibbs free energies of H_2O transfer from H_2O to different organic solvents

I. Computational methods

The free energy of solvation was the free energy of transfer from the gas phase to the condensed phase. It was contributed by the free energy of cavity-dispersion-solvent-structure (CDS) and free energy of electronic and polarization (EP) (seen Figure S41 and Figure S42).[9] We defined the transfer energy of water in different solvents and relative (to water) transfer energies as follows:

$$E_{transfer} = SPE_{solvent} - SPE_{gas} \text{ where SPE represents single-point energy}$$

$$\Delta E = E_{transfer\ (solvent)} - E_{transfer\ (water)}$$

II. Computational results

Table S8. Absolute single point energies(in Eh), EP, CDS (in kcal/mol) of water in the gas phase and in various implicit solvents.

Solvents/gas	SPE	EP	CDS
gas	–76.4769882		
H_2O	–76.4893725	–11.06945956	1.4574
MeCN	–76.4849286	–5.080319139	–0.37331
DCM	–76.4846346	–5.061406018	–0.1912
IPA	–76.4888828	–9.164743845	0.41442
THF	–76.4837999	–3.533007447	–0.99671
EA	–76.4836703	–3.381074968	–1.04712
Dioxane	–76.4818642	–2.112578074	–1.04359
Acetone	–76.4849195	–4.48386556	–0.87774
MeOH	–76.4896311	–10.78957172	1.09514
EtOH	–76.4891731	–9.87802705	0.75067
DMF	–76.4847005	–4.053538713	–1.11737
DMA	–76.4848469	–4.051574609	–1.20759
DMSO	–76.4845914	–4.078620247	–1.02594

SPE: Single point energy, **EP:** CPCM Dielectric**, CDS**: SMD CDS free energy correction energy

Table S9. Absolute (**E**) and relative (to water. Δ**E**) transfer energies (in kcal/mol) of water in different solvents.

Solvents	E	ΔE
H_2O	–7.77128	0
MeCN	–4.98271	2.78857
DCM	–4.79817	2.973109
IPA	–7.46399	0.307294
THF	–4.27442	3.496855
EA	–4.19306	3.578216
Dioxane	–3.05974	4.711538
Acetone	–4.97699	2.794288
MeOH	–7.93353	–0.16225
EtOH	–7.64615	0.125132
DMF	–4.83954	2.931742
DMA	–4.93142	2.839858
DMSO	–4.77107	3.000209

E: transfer energy of gas water, Δ**E**: the difference of transfer energy between solvent and liquid water

Table S10. Relative permittivity (F/m) and dipole moment (D) of water in different solvents. Data from the website of the dielectric constant of common solvents.[10,11]

Solvents	ε	μ
H_2O	80.1	1.82
MeCN	37.5	3.45
DCM	8.93	1.55
IPA	17.9	1.66
THF	7.58	1.75
EA	6.02	1.88
Dioxane	2.25	0.45

Acetone	20.7	2.85
MeOH	32.7	1.7
EtOH	24.5	1.69
DMF	36.7	3.86
DMA	37.8	3.72
DMSO	46.7	3.9

ε: relative permittivity, **μ**: dipole moment

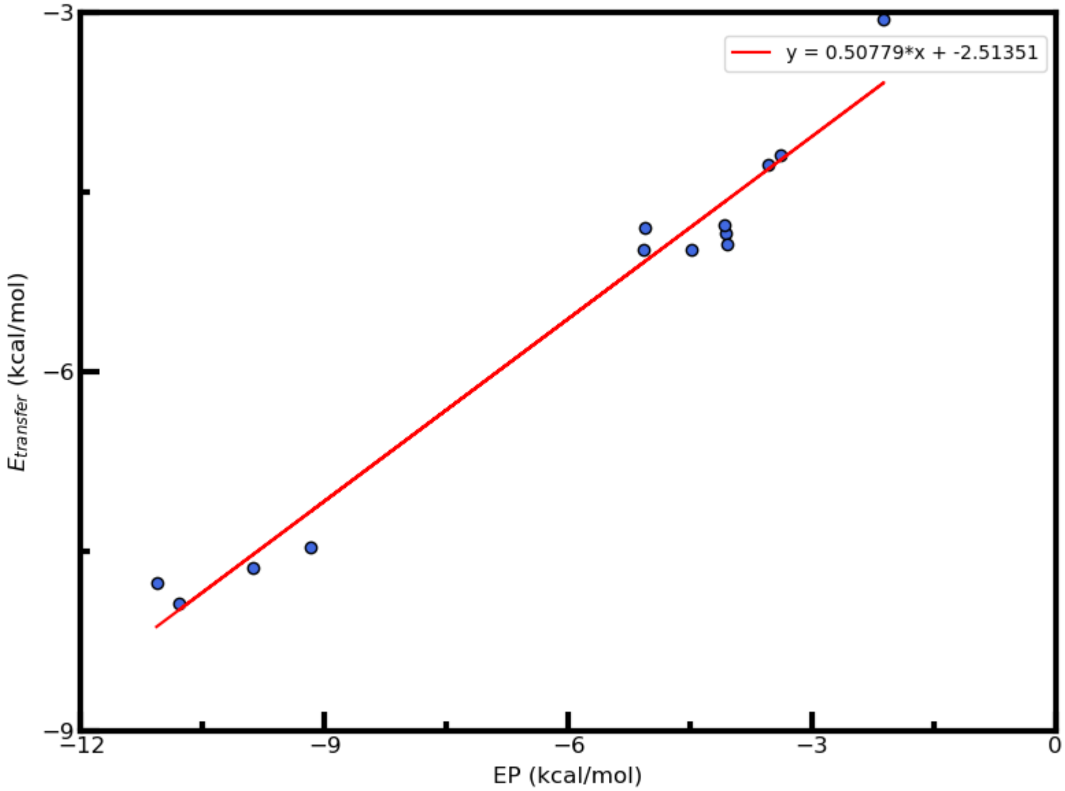

Figure S41. Relation of EP and transfer energies of water in different solvents

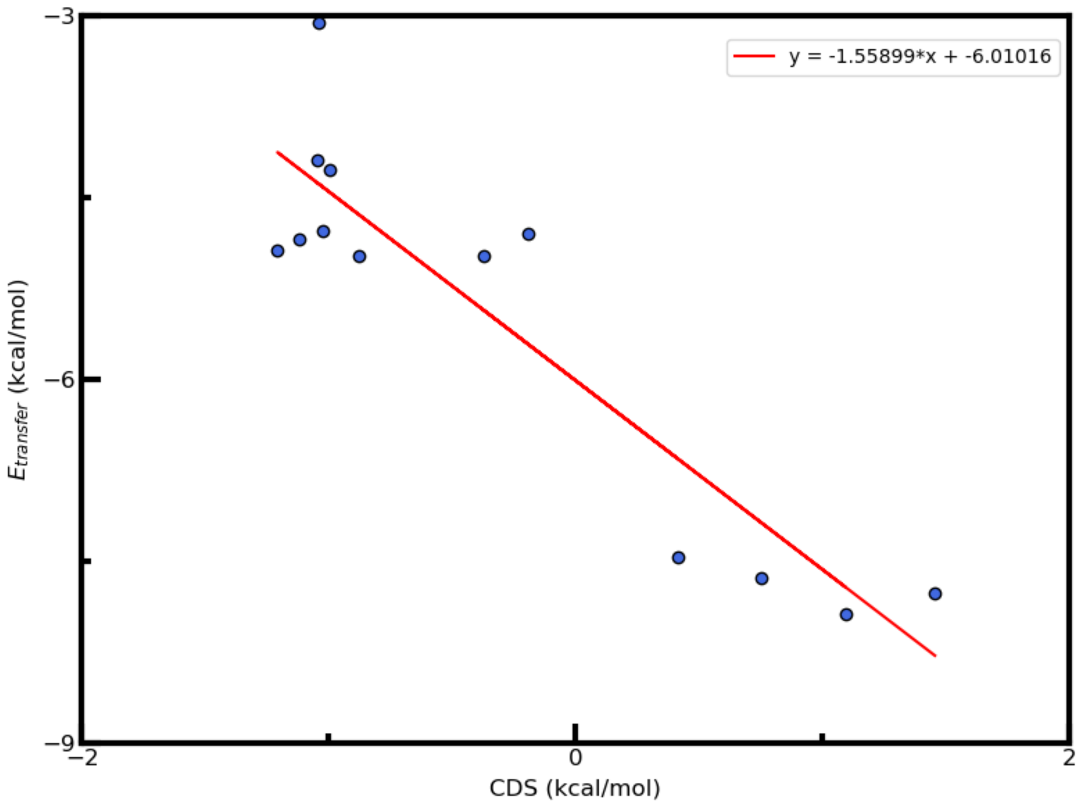

Figure S42. Relation of CDS and transfer energies of water in different solvents

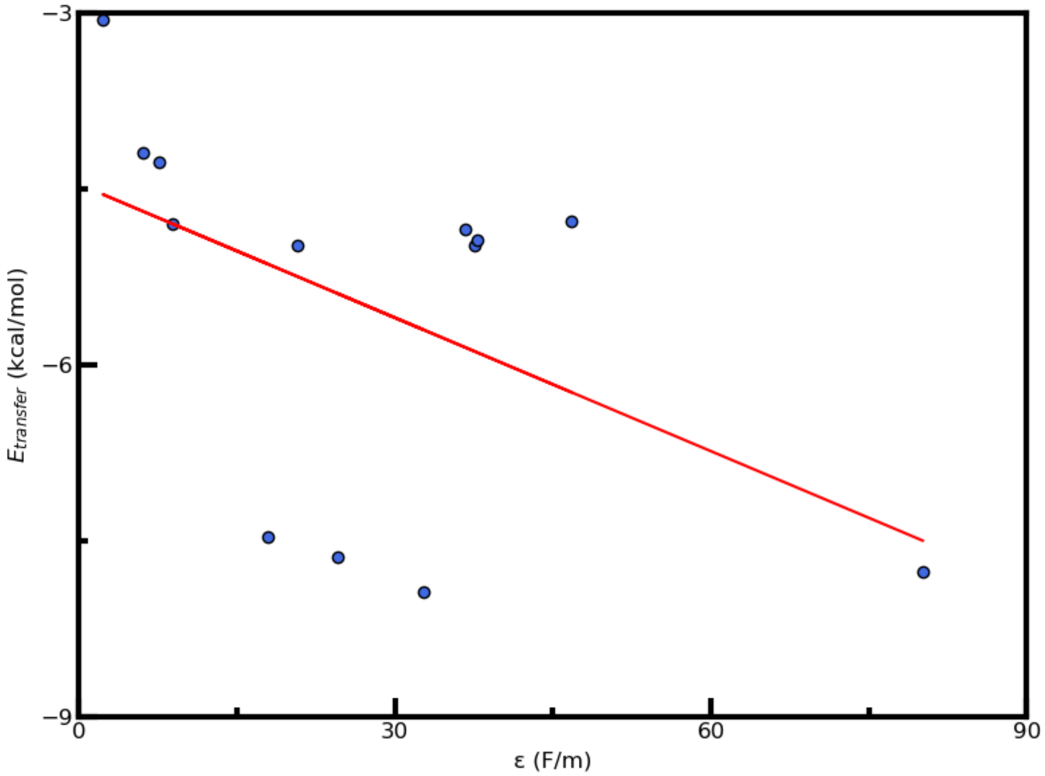

Figure S43. Relation of relative permittivity and transfer energies of water in different solvent

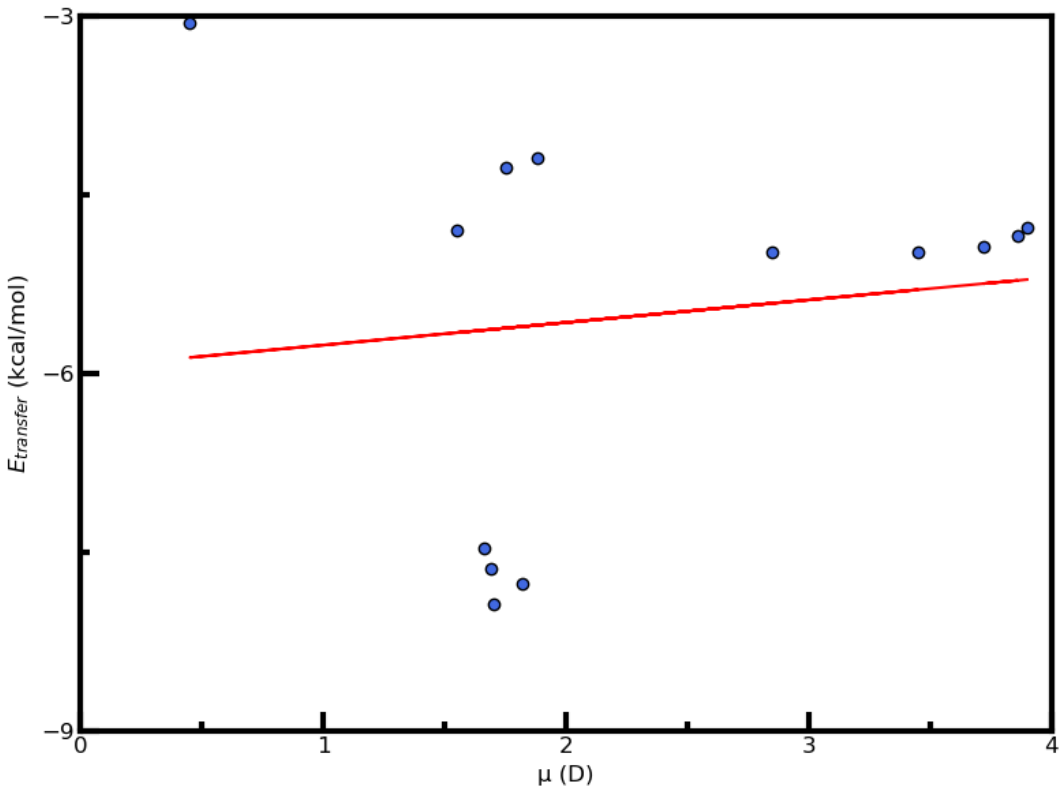

Figure S44. Relation of dipole moment and transfer energies of water in different solvent

Table S11. Gibbs free energies of H_2O transfer from H_2O to different organic solvents.[9,12]

solvent	$\Delta G_{H2O(aq \rightarrow organic\ solvent)}$[a]
DCM	2.97
IPA	0.31
THF	3.50
EA	3.58
Dioxane	4.71
Acetone	2.79
MeOH	–0.16
EtOH	0.13
MeCN	2.79
DMF	2.93
DMA	2.84
DMSO	3.00

[a]All values are in kcal mol^{-1} from the calculation.

5e. Estimation of $E_{H2O/O2}$ based on OCP measurements of $E_{H^+/H2}$

An example for estimating the $E_{H2O/O2}$ in MeCN:

The reduction potential for O_2/H_2O in MeCN may be estimated using i) the standard aqueous cell potential for $O_2 + 2\ H_2 \rightarrow 2\ H_2O$, ii) the measurement of the OCP for H^+/H_2 (E_{H^+/H_2}) with different

conditions (Table S8), and iii) the Gibbs free energy to transfer H_2O from H_2O to MeCN (Table S12). For example, $E_{H2O/O2}$ for the MeCN solution in the presence of 0 M H_2O is calculated by the following equations:

$2\ H_2O_{(aq)} \rightleftharpoons O_{2(g)} + 2\ H_{2(g)}$ (i)
$[E^{\circ}_{H2O/O2(aq)} = 1.23$ V vs $E_{H^+/H_2(aq)}$, $\Delta G^{\circ}_{H2O/O2(aq)} = -113.46$ kcal mol^{-1}]

$H_{2(g)} \rightleftharpoons 2\ H^{+}_{(MeCN)} + 2\ e^{-}_{(V\ vs\ Fc^{+/0})}$ (ii)
$[E_{H^+/H2(MeCN)} = -0.295$ V vs $Fc^{+/0}$, $2 \times \Delta G_{H^+/H2(MeCN)} = 27.21$ kcal mol^{-1}]

$2\ H_2O_{(MeCN)} \rightleftharpoons 2\ H_2O_{(aq)}$ (iii)
$[2 \times \Delta G_{H_2O(aq \rightarrow MeCN)} = 5.58$ kcal mol^{-1}]

$2\ H_2O_{(MeCN)} \rightleftharpoons O_{2(g)} + 4\ e^{-}_{(V\ vs\ Fc^{+/0})} + 4\ H^{+}_{(MeCN)}$ (iv)
$\Delta G_{H2O/O2(MeCN)} = -80.67$ kcal mol^{-1}, $\mathbf{E_{H2O/O2(MeCN)} = 0.87}$ **V vs $Fc^{+/0}$**

The potentials from equations i) and ii) are converted into free energy using the Nernst equation. After summing and balancing the free energy equations, they are converted back into potentials. All the organic solvents listed below are calculated using this method.

Table S12. The oxidation potential for H_2O/O_2 ($E_{H2O/O2}$) in organic solvents (calculated from E_{H^+/H_2} with 0 M, 0.01 M, 0.1 M, 1 M and 10 M H_2O).

solvent	**0 M H_2O[a]**	0.01 M H_2O[b]	0.1 M H_2O[b]	**1 M H_2O[b]**	10 M H_2O[b]
DCM	**0.21**	–	–	**–**	–
IPA	**0.53**	0.88	0.89	**0.87**	0.86
THF	**0.36**	0.92	0.90	**0.85**	0.76
EA	**0.68**	–	–	**–**	–
Dioxane	**0.40**	0.81	0.76	**0.72**	0.69
Acetone	**0.54**	0.79	0.77	**0.74**	0.65
MeOH	**0.34**	0.79	0.77	**0.78**	0.75
EtOH	**0.45**	0.84	0.85	**0.85**	0.79
MeCN	**0.87**	1.13	1.10	**1.10**	1.01
DMF	**0.60**	0.73	0.73	**0.72**	0.67
DMA	**0.13**	0.67	0.67	**0.67**	0.63
DMSO	**0.16**	0.15	0.15	**0.15**	0.15

[a]All potentials are referenced to $Fc^{+/0}$ in V. [b]All potentials are referenced to Ag/AgCl in V, except for DMSO, which is referenced to $Fc^{+/0}$ in V.

6. Estimation of the Thermodynamic Reduction Potential of O_2/H_2O at Non-standard State (acidic and alkaline conditions)

6a. General Considerations

The H^+/H_2 potential in organic media can be determined using a recently reported protocol through open-circuit potential (OCP) measurements at a Pt electrode.[8] (Figure S45.) The H^+/H_2 potentials were measured for various solvents containing [DMF-H][OTf] and [NaOH] under 1 atm H_2 (local atmospheric pressure of 756 mm Hg, correction to 1 atm < 1 mV). All solutions contained 0.1 M $[NBu_4][PF_6]$ or 0.1 M $[NBu_4][BF_4]$ supporting electrolyte. Dioxane is employed with an electrolyte concentration of 0.3 M tetrabutylammonium tetrafluoroborate ($[NBu_4][BF_4]$). A stable OCP was observed, and this potential was corrected to be versus $Ag/AgNO_3$ or Ag/AgCl (KCl, 3 M).

The pre-treatment method for Pt wire was referenced from the literature: *Inorg. Chem.* **2013**, *52*, 3823−3835. The platinum wire electrodes used for OCP (BASI; 99.95%, 0.5 mm diam, ~60 mm length) were prepared by rinsing with acetone, drying in air, immersing in freshly prepared aqua regia for 30 min, then rinsing with flowing deionized water for 5 min. Each wire was then clamped into a stainless steel hemostat and heated to a uniform yellow-orange glow in a flame. Once the entire wire was at the maximum temperature attainable (judged by color), the flame was extinguished, and the wire was allowed to cool in the hydrogen-nitrogen (5% hydrogen/nitrogen) stream until it no longer glowed. At this point, the wire was placed in a screw-cap storage tube maintained under a positive flow of stream. After each wire was treated, the tube was closed and taken into the glovebox. Each wire was useable for at least several measurements, sometimes affording consistent data for up to a day. Unused electrodes remained active for several days if stored in the glovebox. Excessive and increasing signal noise, leading ultimately to a substantial instability of the OCP, was generally taken to indicate deterioration of the electrode response, and the electrode was replaced. Substitution of one electrode with another afforded the same OCP value within 3 mV. The same set of four platinum wire electrodes was reconditioned as described above and reused numerous times with no effect on the OCP measurements.

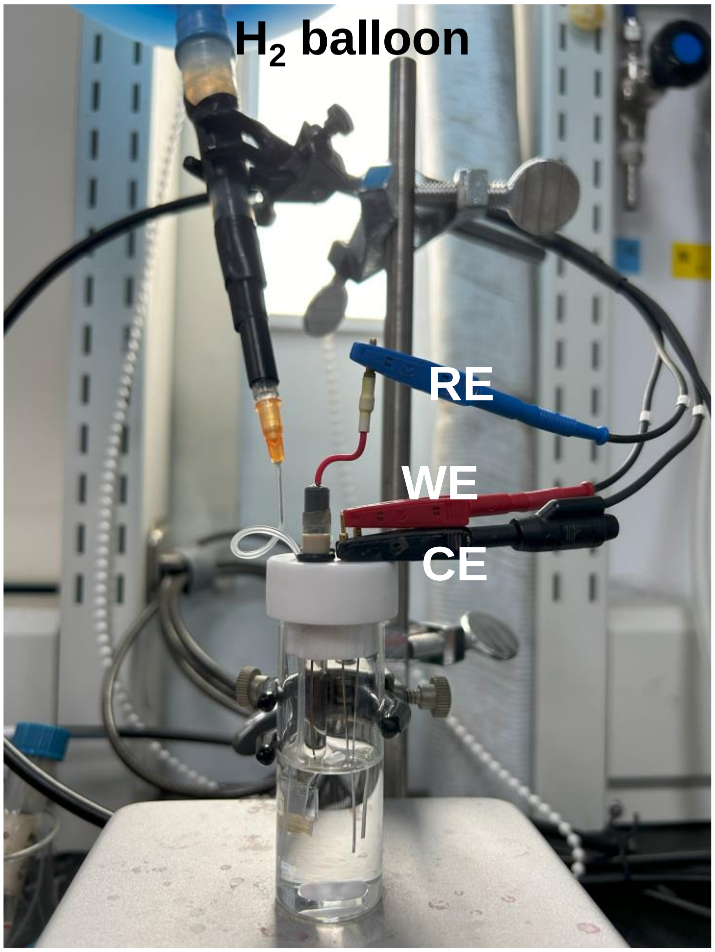

Figure S45. Schematic of the four-electrode cell configuration used for OCP measurements. The reduction potential of H^+/H_2 was measured for a solution containing $[NBu_4][PF_6]$ (0.1 M) under 1 atm H_2.

6b. Thermodynamic Redox Potentials of H_2O/O_2 in Acidic and Alkaline Nonaqueous Media

To broaden the understanding of $E_{H2O/O2(org)}$ and the applications of the WOR under acidic and alkaline conditions in organic media, protonated DMF ([DMF-H][OTf], OTf: triflate) or NaOH was added to the 12 organic solvents at different concentrations, following which $E_{H+/H2}$ was recorded via OCP measurements and $E_{H2O/O2(org)}$ was derived. The selection of [DMF-H][OTf] and NaOH was intended to reduce homoconjugation as they were a strong acid and a base, respectively.[13-15]

In the presence of [DMF-H][OTf], most of the $E_{H2O/O2(org)}$ values showed an expected Nernstian behavior of 60 mV/log([DMF-H][OTf]) over the concentration range of 0.001–0.1. In the tests conducted under basic conditions, the maximum concentration of NaOH was limited to 100 mM owing to the solubility of the SOs in the 12 organic solvents.

Essentially, these results demonstrated that $E_{H2O/O2(org)}$ was affected by the concentration of acid and base, similar to the behavior of $E_{H2O/O2}$ under aqueous conditions. While a Pourbaix diagram depicts the correlation between $E_{H2O/O2}$ and pH for aqueous solutions, the behavior of $E_{H2O/O2(org)}$ in organic solutions may be best described by the decade of [acid/base] for the same acid/base.

6c. The OCP measurement of H^+/H_2 redox couples (E_{H^+/H_2}) under acidic conditions ([DMF-H][OTf]: 1−1000 mM)

Table S13. OCP of H^+/H_2 redox couples[a] (E_{H^+/H_2}) vs. $Fc^{+/0}$ with various concentrations of [DMF-H][OTf] (1−1000 mM).

Solvent/ conc. (mM)	1	5	10	50	100	500	1000
IPA	–0.613	–0.546	–0.524	–0.494	–0.483	–0.472	–0.459
MeOH	–0.541	–0.507	–0.488	–0.430	–0.413	–0.398	–0.393
EtOH	–0.559	–0.543	–0.533	–0.492	–0.496	–0.461	–0.446
MeCN	0.041	0.098	0.107	0.141	0.149	0.175	0.183
DMF	–0.844	–0.811	–0.794	–0.755	–0.738	–0.694	–0.676
DMA	–0.723	–0.710	–0.702	–0.677	–0.664	–0.623	–0.599
DMSO	–0.777	–0.756	–0.712	–0.690	–0.680	–0.644	–0.616

[a]All potentials averaged from triplicate measurements and are referenced to $Fc^{+/0}$ in V.

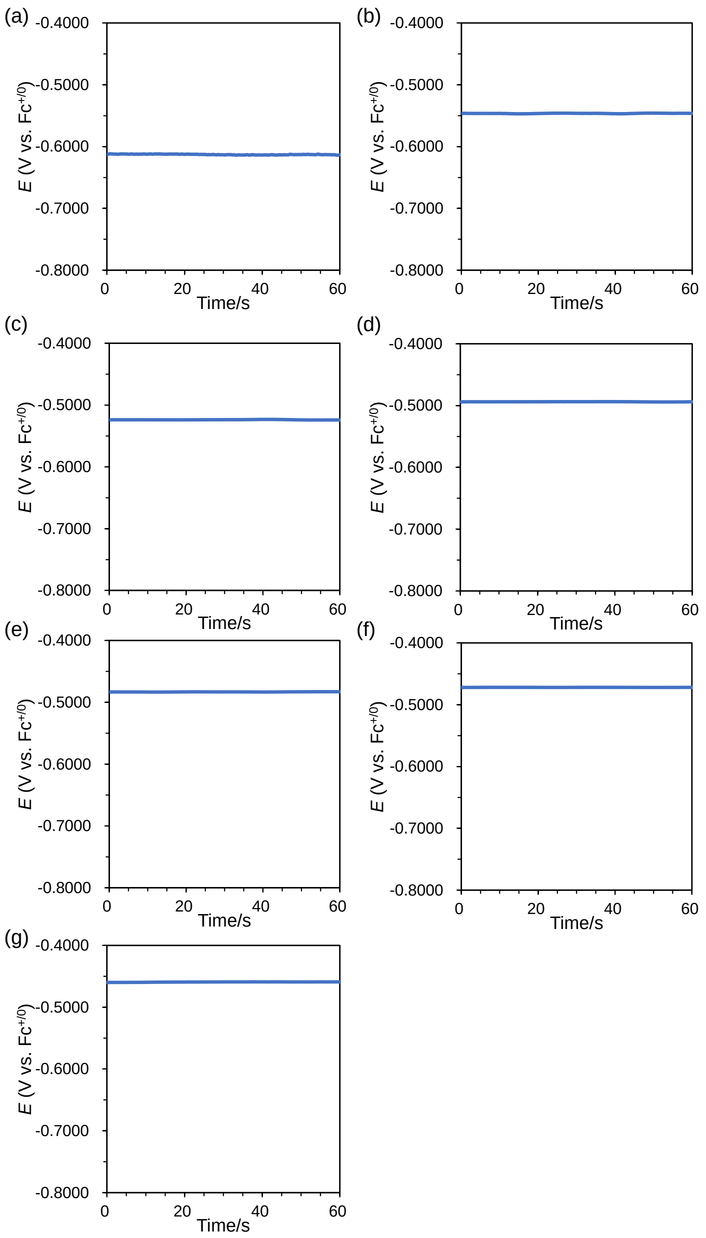

Figure S46. The blue traces show the OCP of E_{H^+/H_2} in IPA solutions under 1 atm H_2. (a) 1 mM, (b) 5 mM, (c) 10 mM, (d) 50 mM, (e) 100 mM, (f) 500 mM, and (g) 1000 mM [DMF-H][OTf].

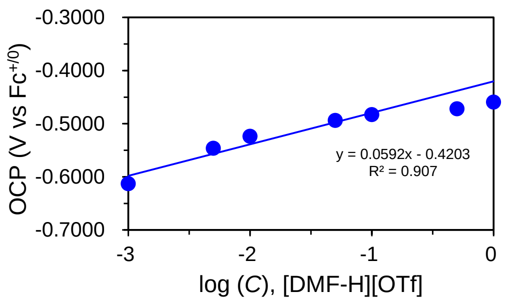

Figure S47. OCP experiment values versus log (*C*), [DMF-H][OTf] in IPA.

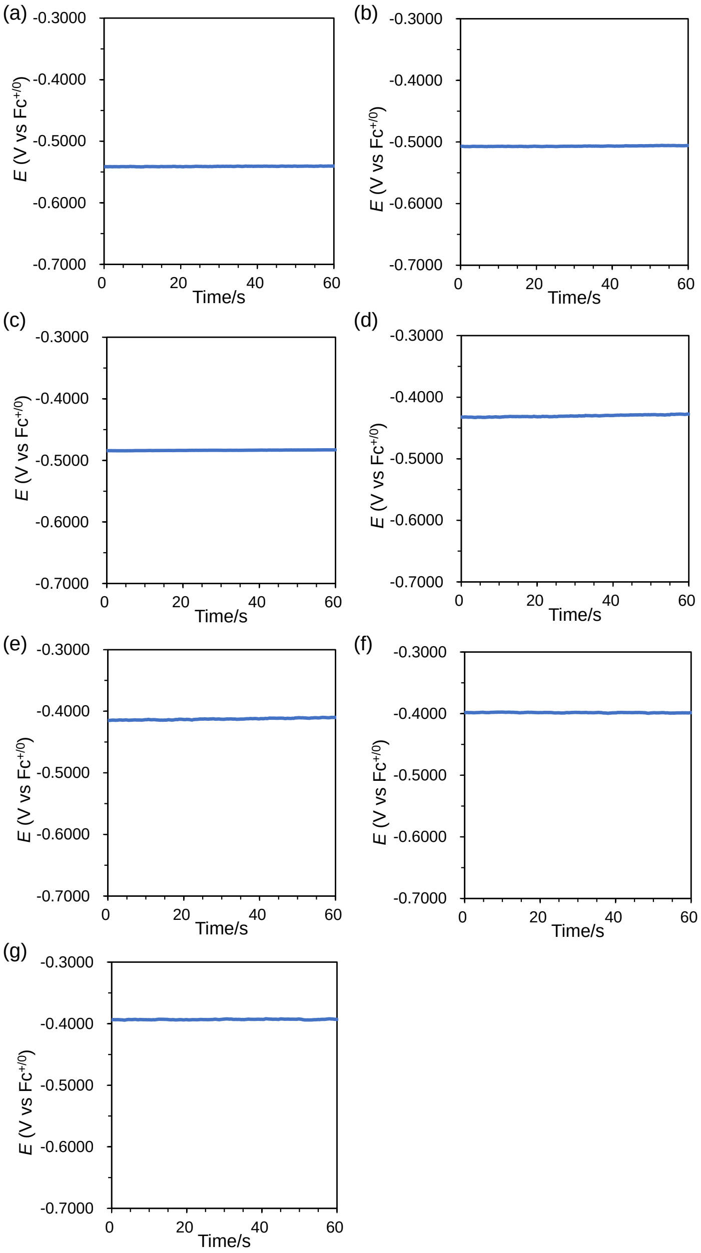

Figure S48. The blue traces show the OCP of E_{H^+/H_2} in MeOH solutions under 1 atm H_2. (a) 1 mM, (b) 5 mM, (c) 10 mM, (d) 50 mM, (e) 100 mM, (f) 500 mM, and (g) 1000 mM [DMF-H][OTf].

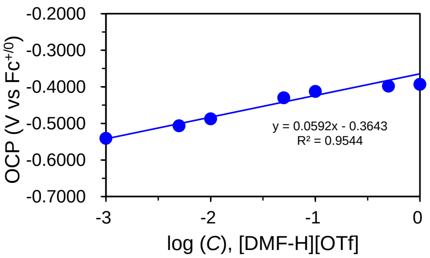

Figure S49. OCP experiment values versus log (*C*), [DMF-H][OTf] in MeOH.

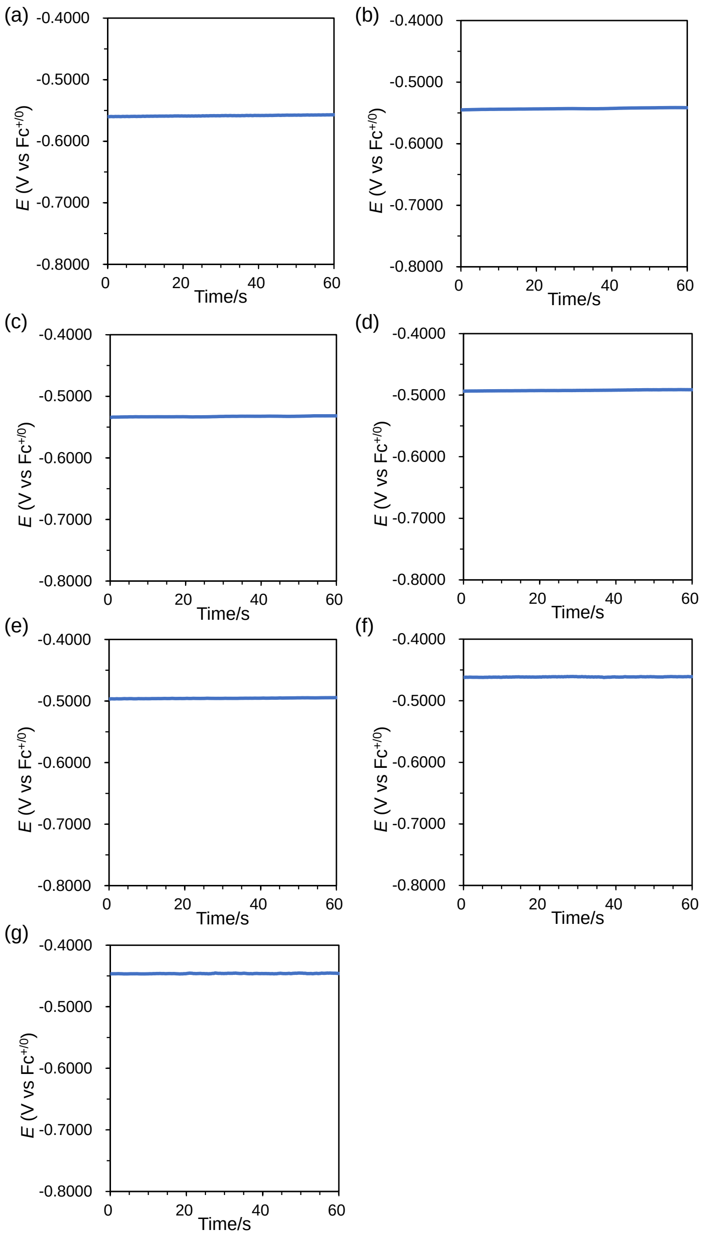

Figure S50. The blue traces show the OCP of E_{H^+/H_2} in EtOH solutions under 1 atm H_2. (a) 1 mM, (b) 5 mM, (c) 10 mM, (d) 50 mM, (e) 100 mM, (f) 500 mM, and (g) 1000 mM [DMF-H][OTf].

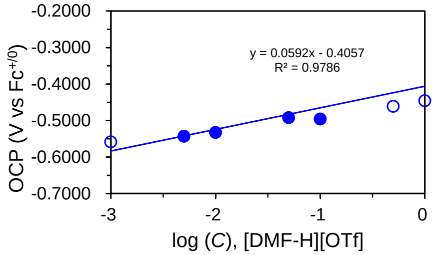

Figure S51. OCP experiment values versus log (C), [DMF-H][OTf] in EtOH.

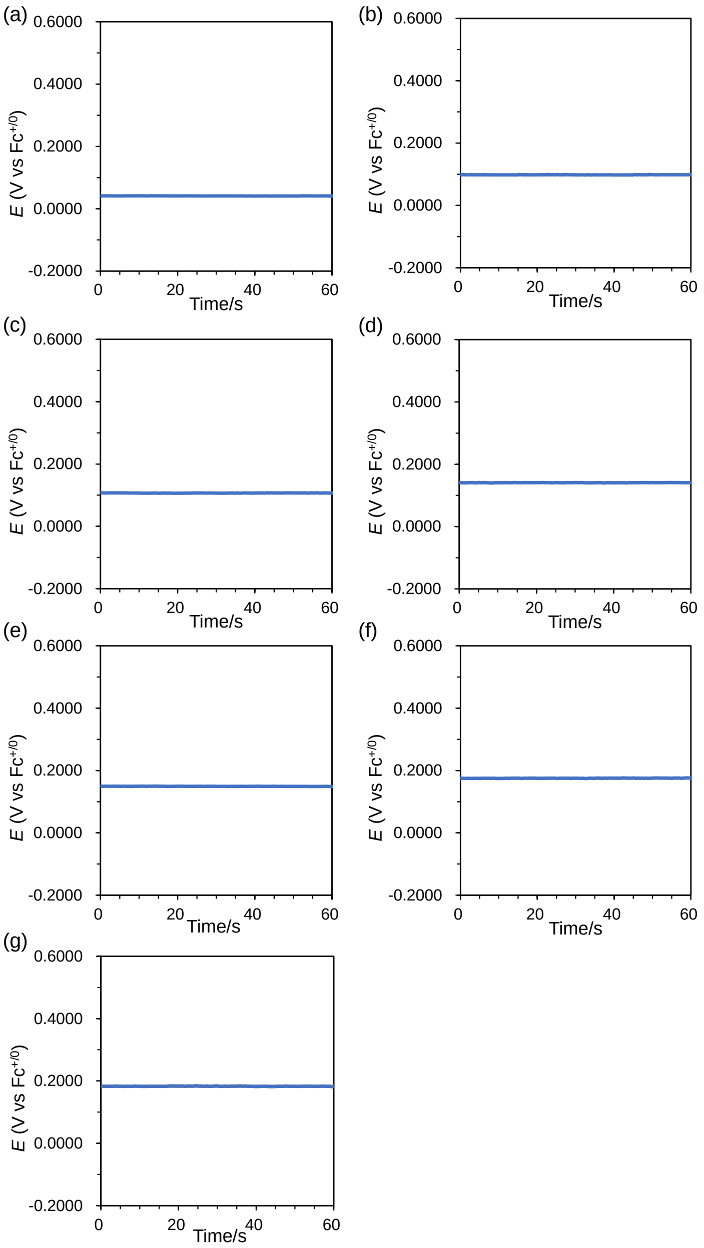

Figure S52. The blue traces show the OCP of E_{H^+/H_2} in MeCN solutions under 1 atm H_2. (a) 1 mM, (b) 5 mM, (c) 10 mM, (d) 50 mM, (e) 100 mM, (f) 500 mM, and (g) 1000 mM [DMF-H][OTf].

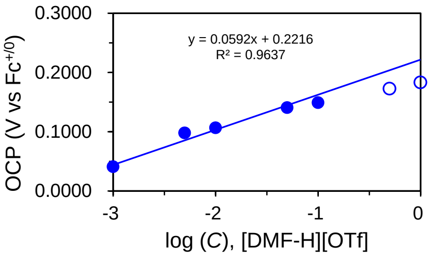

Figure S53. OCP experiment values versus log (*C*), [DMF-H][OTf] in MeCN.

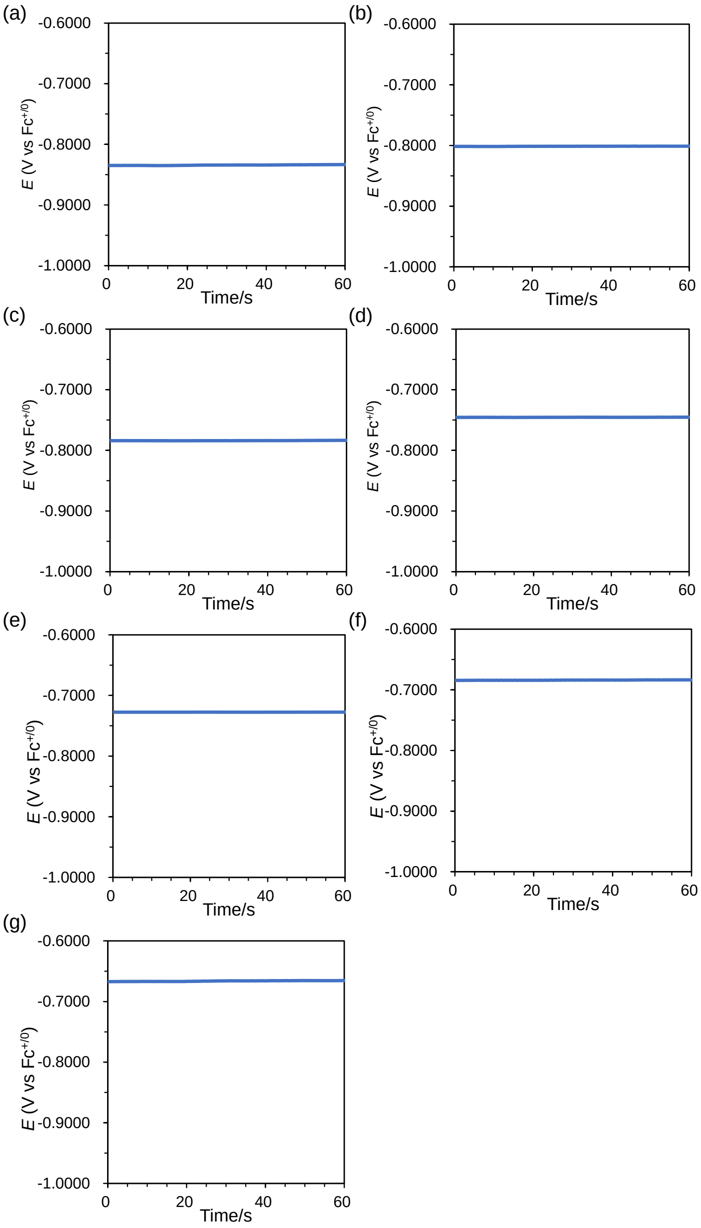

Figure S54. The blue traces show the OCP of E_{H^+/H_2} in DMF solutions under 1 atm H_2. (a) 1 mM, (b) 5 mM, (c) 10 mM, (d) 50 mM, (e) 100 mM, (f) 500 mM, and (g) 1000 mM [DMF-H][OTf].

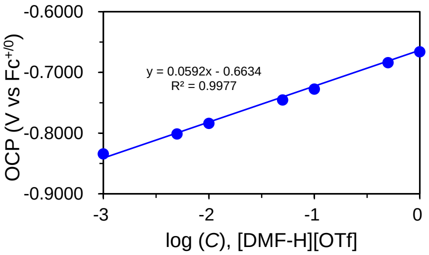

Figure S55. OCP experiment values versus log (*C*), [DMF-H][OTf] in DMF.

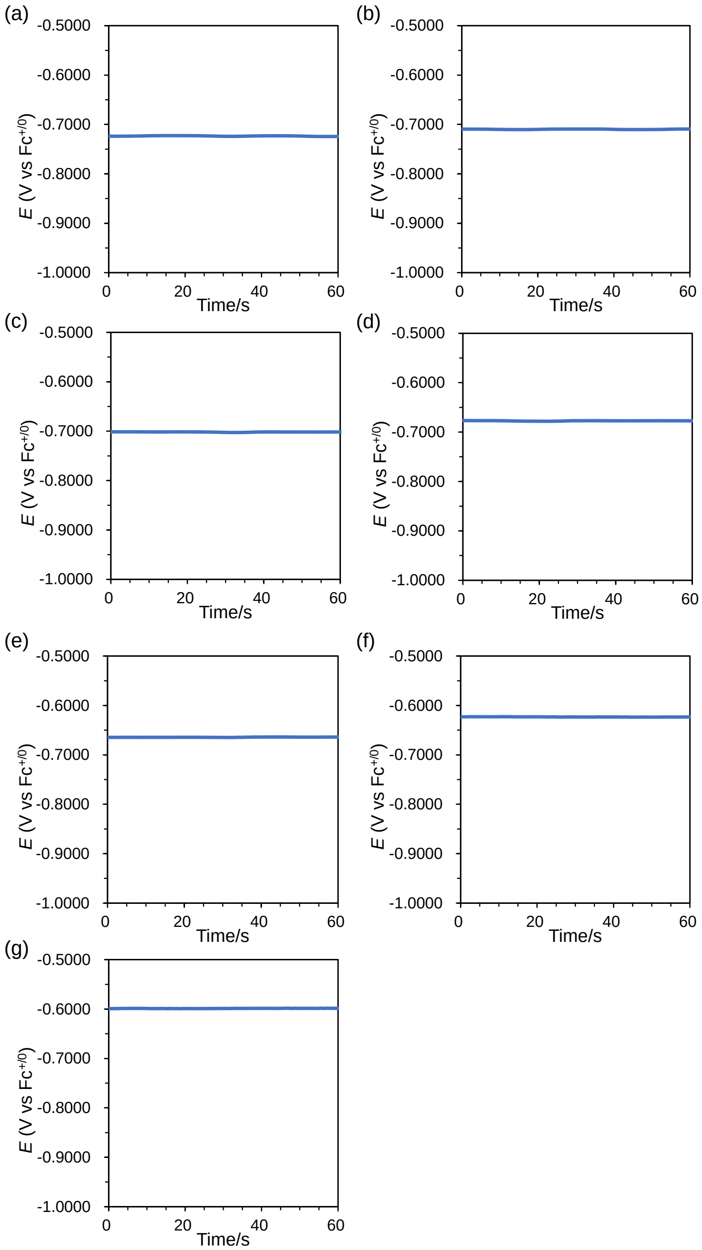

Figure S56. The blue traces show the OCP of E_{H^+/H_2} in DMA solutions under 1 atm H_2. (a) 1 mM, (b) 5 mM, (c) 10 mM, (d) 50 mM, (e) 100 mM, (f) 500 mM, and (g) 1000 mM [DMF-H][OTf].

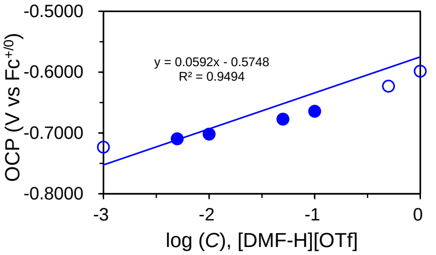

Figure S57. OCP experiment values versus log (C), [DMF-H][OTf] in DMA.

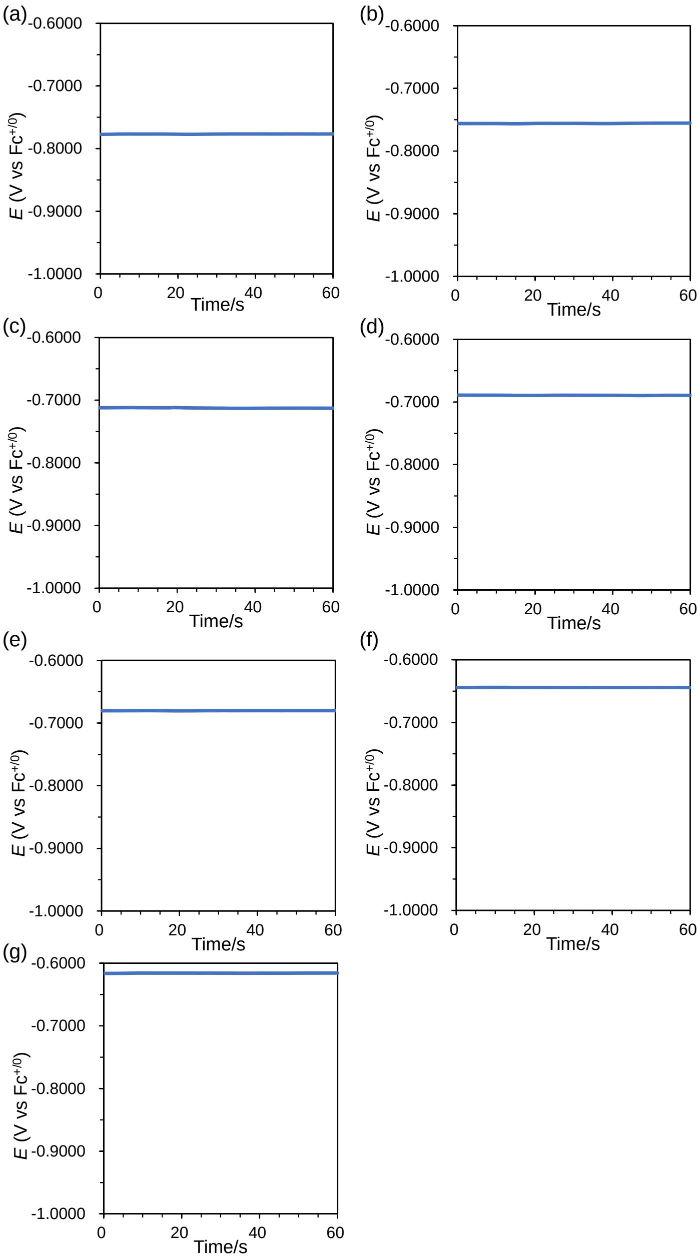

Figure S58. The blue traces show the OCP of E_{H^+/H_2} in DMSO solutions under 1 atm H_2. (a) 1 mM, (b) 5 mM, (c) 10 mM, (d) 50 mM, (e) 100 mM, (f) 500 mM, and (g) 1000 mM [DMF-H][OTf].

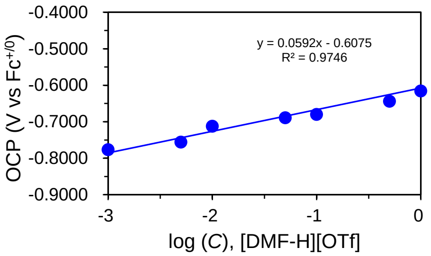

Figure S59. OCP experiment values versus log (C), [DMF-H][OTf] in DMSO.

6d. The OCP measurement of H^+/H_2 redox couples (E_{H^+/H_2}) under alkaline conditions (NaOH: 1−100 mM)

Table S14. OCP of H^+/H_2 redox couples[a] (E_{H^+/H_2}) vs. $Fc^{+/0}$ with various concentrations of [NaOH] (1–100 mM).

Solvent/ conc. (mM)	1	5	10	25	50	100
THF	–0.103	–0.225	–0.235	–0.265	–0.278	–0.324
Dioxane	–0.924	–0.947	–0.985	–1.045	–1.084	–1.097
Acetone	–0.498	–0.506	–0.538	–0.579	–0.604	–0.619
MeOH	–1.233	–1.266	–1.293	–1.310	–1.316	–1.340
EtOH	–1.039	–1.046	–1.068	–1.079	–1.095	insoluble
MeCN	–0.962	–1.007	–1.035	–1.051	–1.039	–1.037
DMF	–1.328	–1.420	–1.433	–1.441	–1.498	–1.480
DMA	–1.336	–1.374	–1.405	–1.419	–1.440	–1.451
DMSO	–1.134	–1.139	–1.162	–1.170	insoluble	insoluble

[a]All potentials averaged from triplicate measurements and are referenced to $Fc^{+/0}$ in V.

Table S15. OCP of H^+/H_2 redox couples[a] (E_{H^+/H_2}) vs. $Fc^{+/0}$ with various concentrations of [NaOH] (1–10 mM).

Solvent/ conc. (mM)	1	3	5	7	10
IPA	–1.139	–1.173	–1.189	–1.233	–1.235

[a]All potentials averaged from triplicate measurements and are referenced to $Fc^{+/0}$ in V.

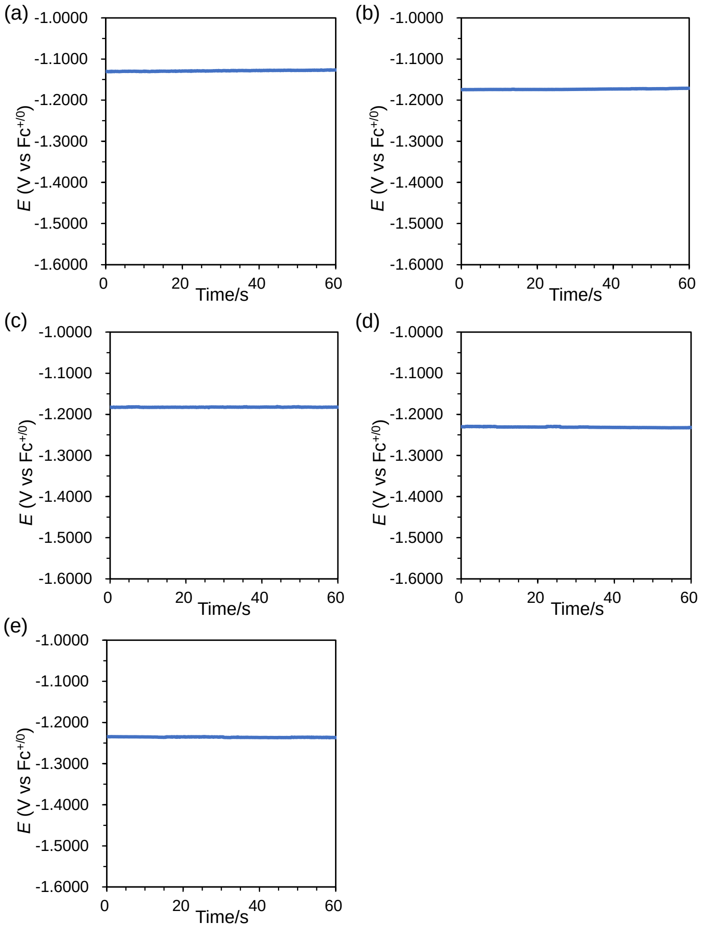

Figure S60. The blue traces show OCP of E_{H^+/H_2} in IPA solutions under 1 atm H_2. (a) 1 mM NaOH and H_2O (0.02%, v/v); (b) 3 mM NaOH and H_2O (0.06%, v/v); (c) 5 mM NaOH and H_2O (0.1%, v/v); (d) 7 mM NaOH and H_2O (0.14%, v/v); and 10 mM NaOH and H_2O (0.2%, v/v).

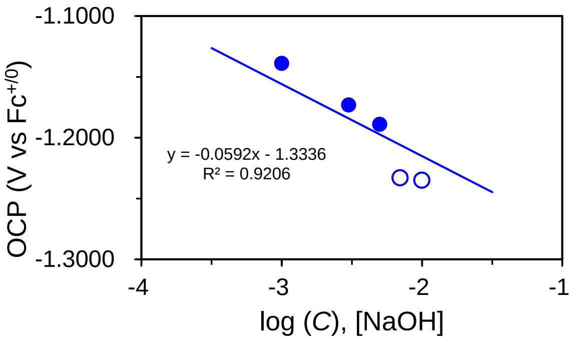

Figure S61. OCP experiment values versus log (*C*), [NaOH] in IPA.

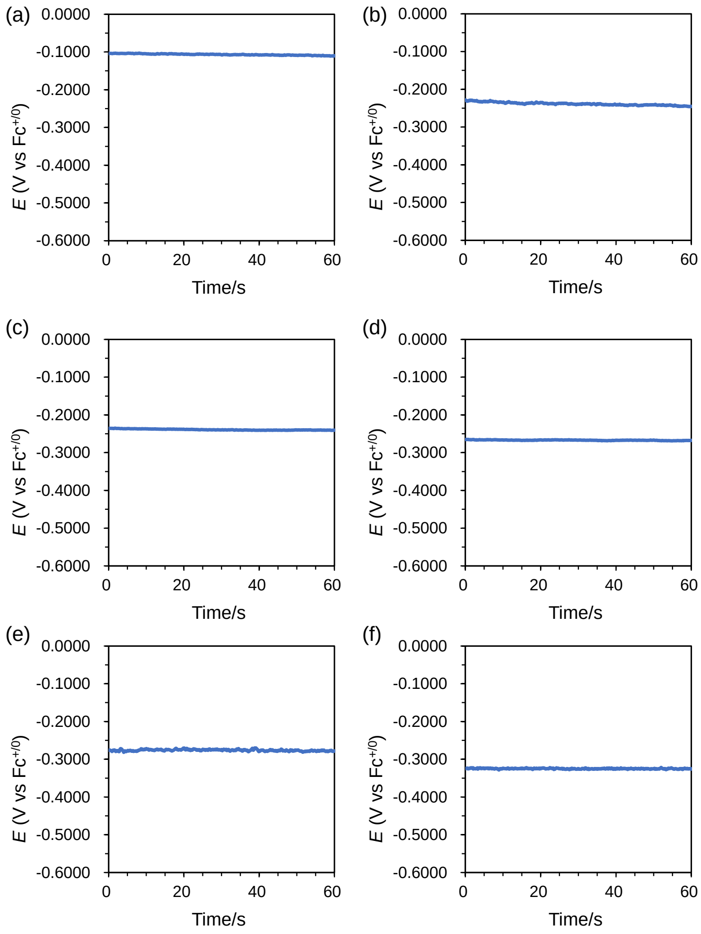

Figure S62. The blue traces show OCP of E_{H^+/H_2} in THF solutions under 1 atm H_2. (a) 1 mM NaOH and H_2O (0.02%, v/v); (b) 5 mM NaOH and H_2O (0.1%, v/v); (c) 10 mM NaOH and H_2O (0.2%, v/v); (d) 25 mM NaOH and H_2O (0.5%, v/v); (e) 50 mM NaOH and H_2O (1%, v/v) and (f) 100 mM NaOH and H_2O (2%, v/v).

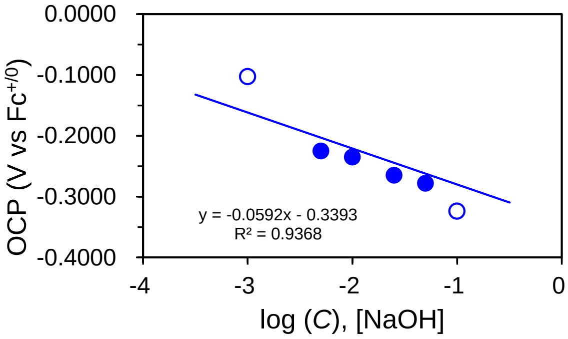

Figure S63. OCP experiment values versus log (C), [NaOH] in THF.

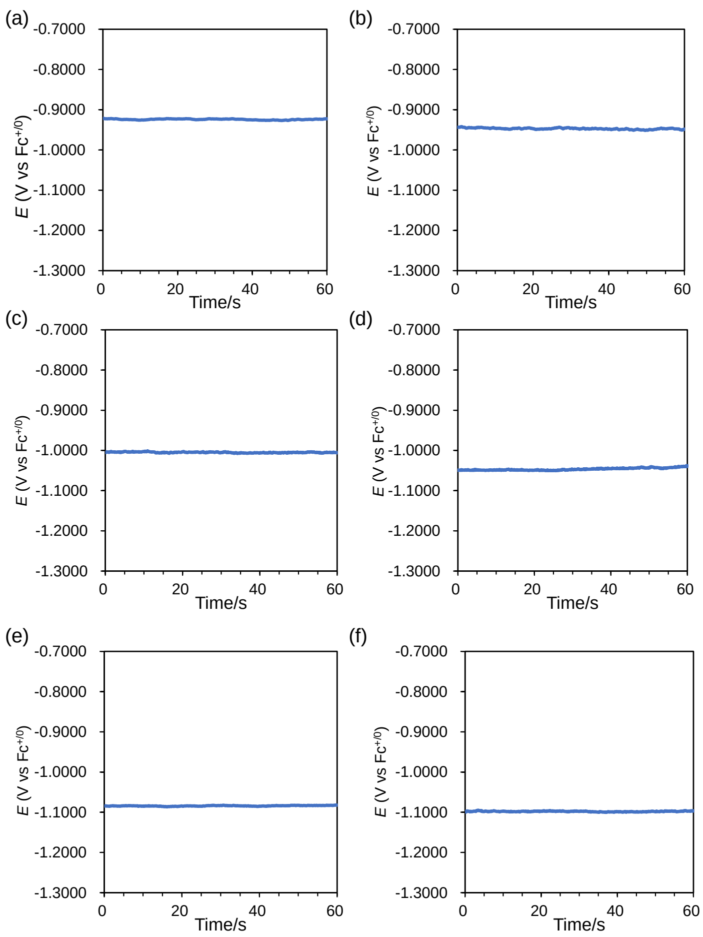

Figure S64. The blue traces show OCP of E_{H^+/H_2} in Dioxane solutions under 1 atm H_2. (a) 1 mM NaOH and H_2O (0.02%, v/v); (b) 5 mM NaOH and H_2O (0.1%, v/v); (c) 10 mM NaOH and H_2O (0.2%, v/v); (d) 25 mM NaOH and H_2O (0.5%, v/v); (e) 50 mM NaOH and H_2O (1%, v/v) and (f) 100 mM NaOH and H_2O (2%, v/v).

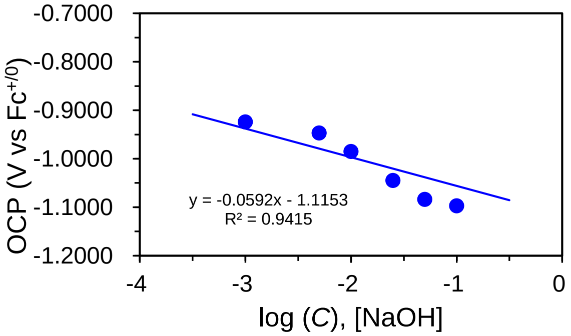

Figure S65. OCP experiment values versus log (C), [NaOH] in Dioxane.

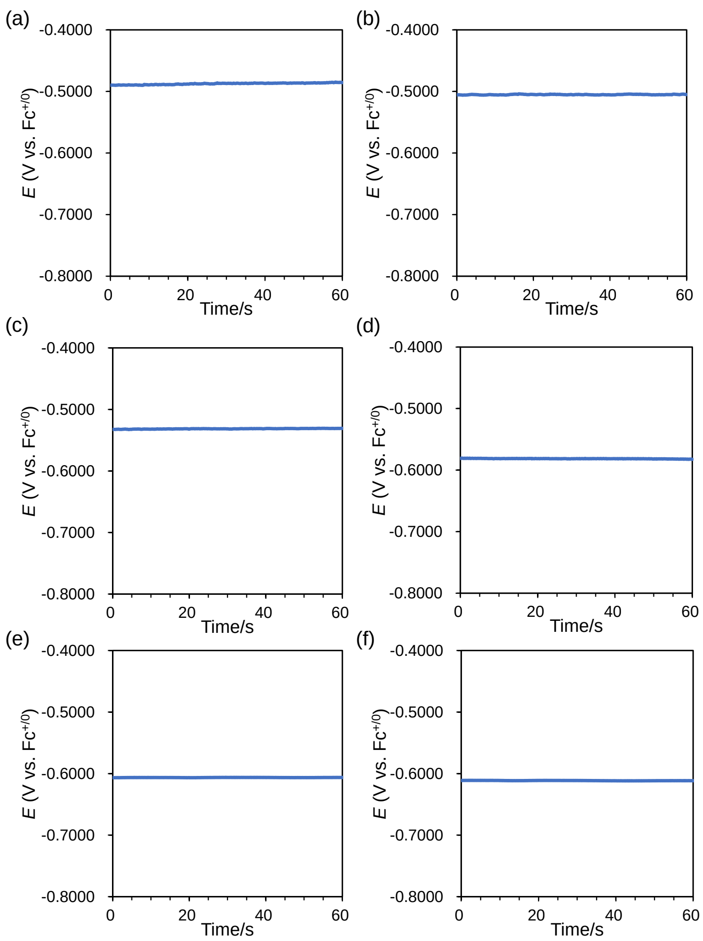

Figure S66. The blue traces show OCP of E_{H^+/H_2} in Acetone solutions under 1 atm H_2. (a) 1 mM NaOH and H_2O (0.02%, v/v); (b) 5 mM NaOH and H_2O (0.1%, v/v); (c) 10 mM NaOH and H_2O (0.2%, v/v); (d) 25 mM NaOH and H_2O (0.5%, v/v); (e) 50 mM NaOH and H_2O (1%, v/v) and (f) 100 mM NaOH and H_2O (2%, v/v).

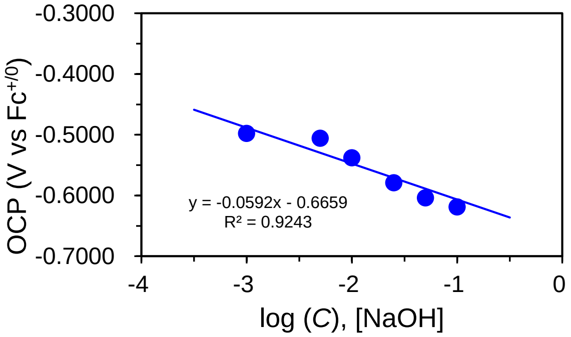

Figure S67. OCP experiment values versus log (*C*), [NaOH] in Acetone.

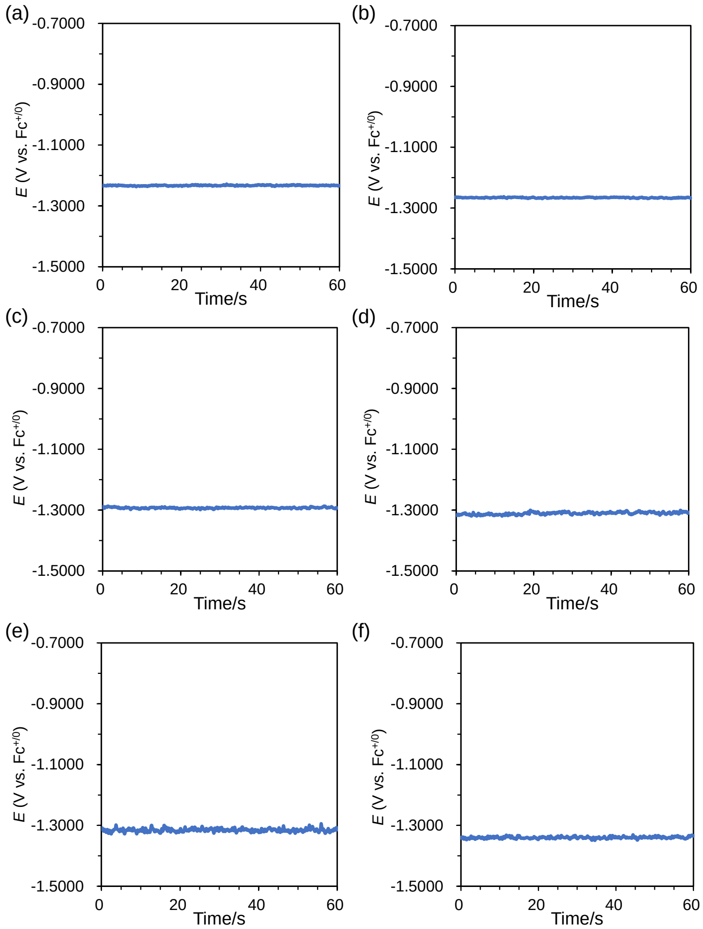

Figure S68. The blue traces show OCP of E_{H^+/H_2} in MeOH solutions under 1 atm H_2. (a) 1 mM NaOH and H_2O (0.02%, v/v); (b) 5 mM NaOH and H_2O (0.1%, v/v); (c) 10 mM NaOH and H_2O (0.2%, v/v); (d) 25 mM NaOH and H_2O (0.5%, v/v); (e) 50 mM NaOH and H_2O (1%, v/v) and (f) 100 mM NaOH and H_2O (2%, v/v).

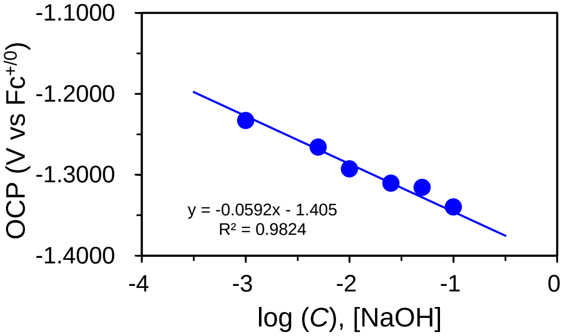

Figure S69. OCP experiment values versus log (*C*), [NaOH] in MeOH.

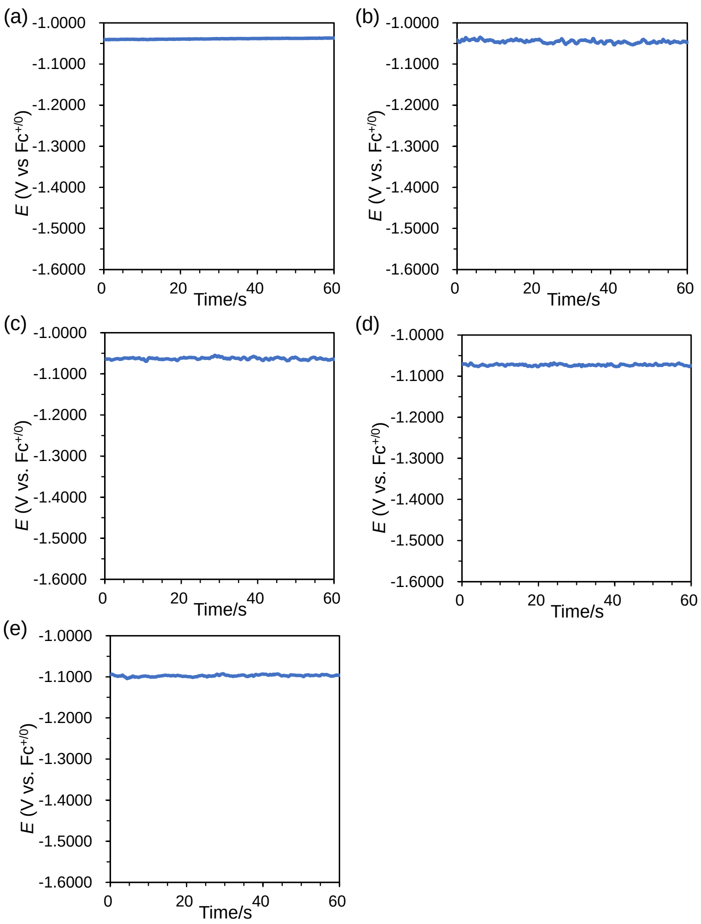

Figure S70. The blue traces show OCP of E_{H^+/H_2} in EtOH solutions under 1 atm H_2. (a) 1 mM NaOH and H_2O (0.02%, v/v); (b) 5 mM NaOH and H_2O (0.1%, v/v); (c) 10 mM NaOH and H_2O (0.2%, v/v); (d) 25 mM NaOH and H_2O (0.5%, v/v); (e) 50 mM NaOH and H_2O (1%, v/v).

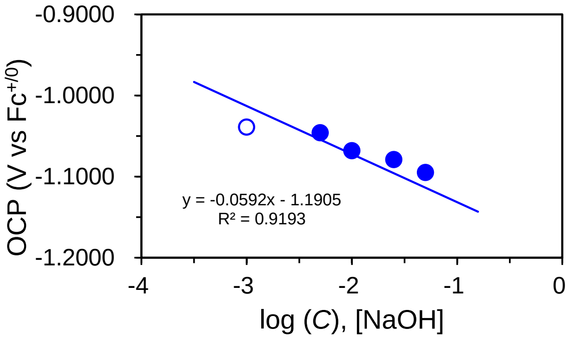

Figure S71. OCP experiment values versus log (*C*), [NaOH] in EtOH.

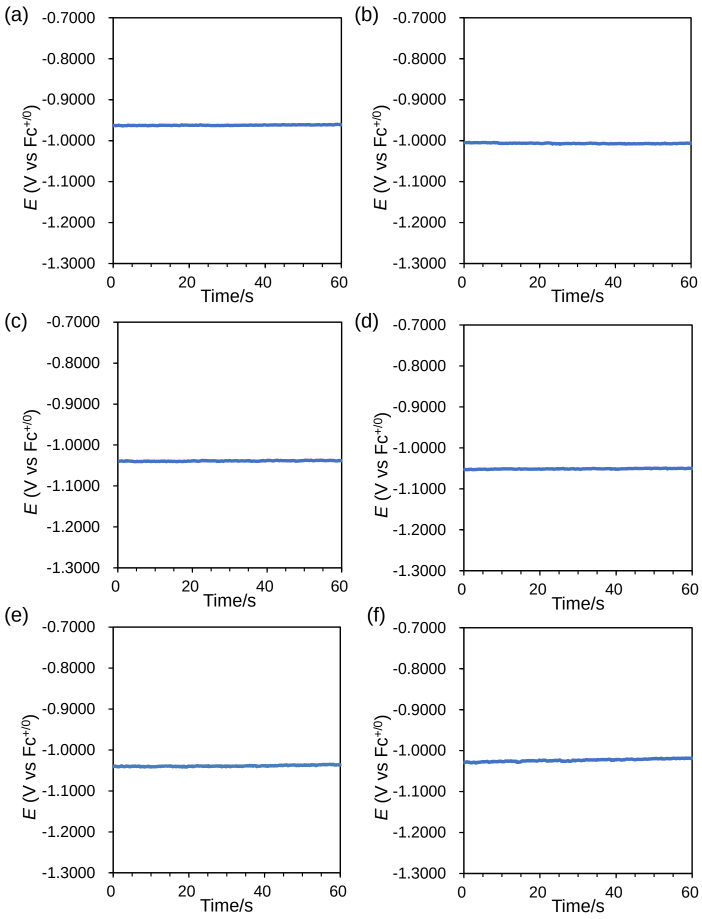

Figure S72. The blue traces show the OCP of E_{H^+/H_2} in MeCN solutions under 1 atm H_2. (a) 1 mM NaOH and H_2O (0.02%, v/v); (b) 5 mM NaOH and H_2O (0.1%, v/v); (c) 10 mM NaOH and H_2O (0.2%, v/v); (d) 25 mM NaOH and H_2O (0.5%, v/v); (e) 50 mM NaOH and H_2O (1%, v/v) and (f) 100 mM NaOH and H_2O (2%, v/v).

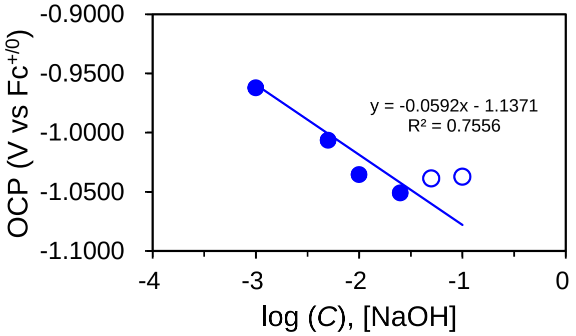

Figure S73. OCP experiment values versus log (*C*), [NaOH] in MeCN.

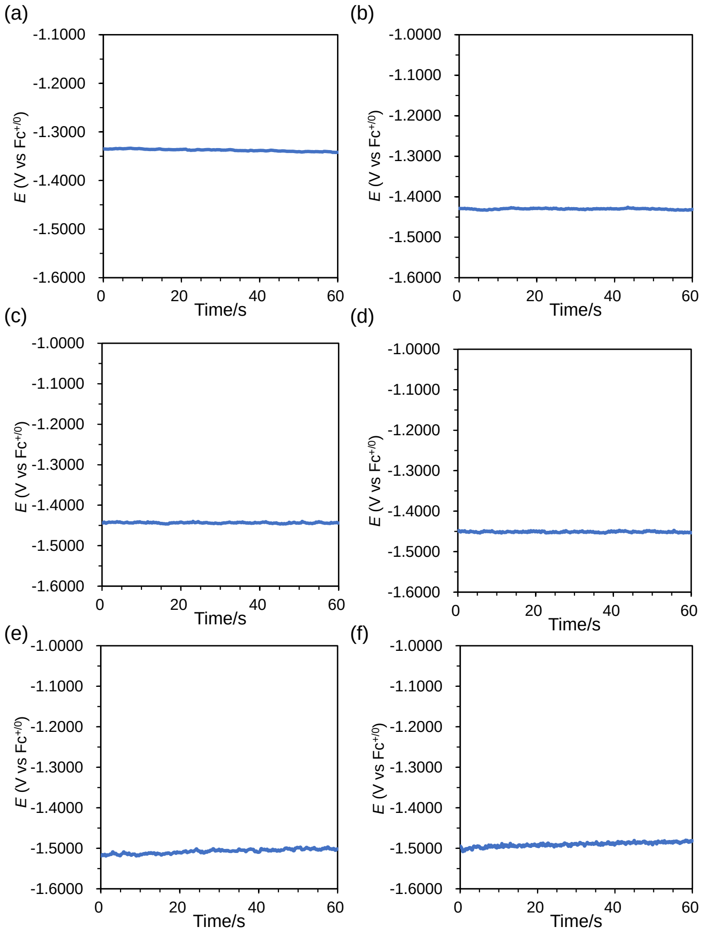

Figure S74. The blue traces show OCP of E_{H^+/H_2} in DMF solutions under 1 atm H_2. (a) 1 mM NaOH and H_2O (0.02%, v/v); (b) 5 mM NaOH and H_2O (0.1%, v/v); (c) 10 mM NaOH and H_2O (0.2%, v/v); (d) 25 mM NaOH and H_2O (0.5%, v/v); (e) 50 mM NaOH and H_2O (1%, v/v) and (f) 100 mM NaOH and H_2O (2%, v/v).

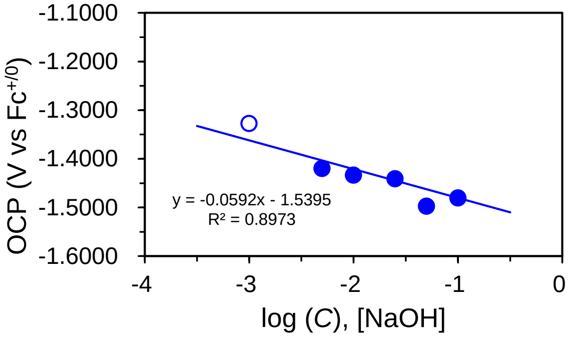

Figure S75. OCP experiment values versus log (*C*), [NaOH] in DMF.

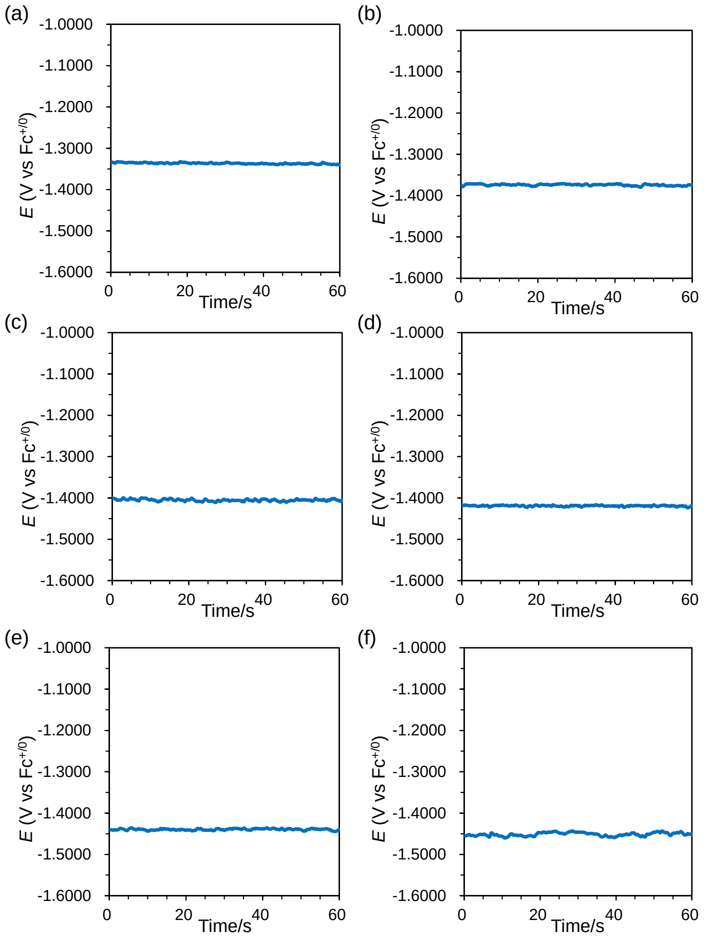

Figure S76. The blue traces show OCP of E_{H^+/H_2} in DMA solutions under 1 atm H_2. (a) 1 mM NaOH and H_2O (0.02%, v/v); (b) 5 mM NaOH and H_2O (0.1%, v/v); (c) 10 mM NaOH and H_2O (0.2%, v/v); (d) 25 mM NaOH and H_2O (0.5%, v/v); (e) 50 mM NaOH and H_2O (1%, v/v) and (f) 100 mM NaOH and H_2O (2%, v/v).

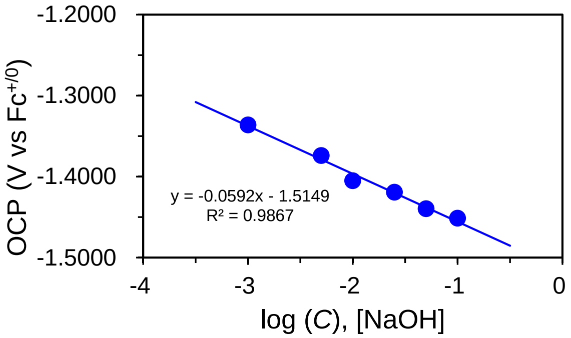

Figure S77. OCP experiment values versus log (*C*), [NaOH] in DMA.

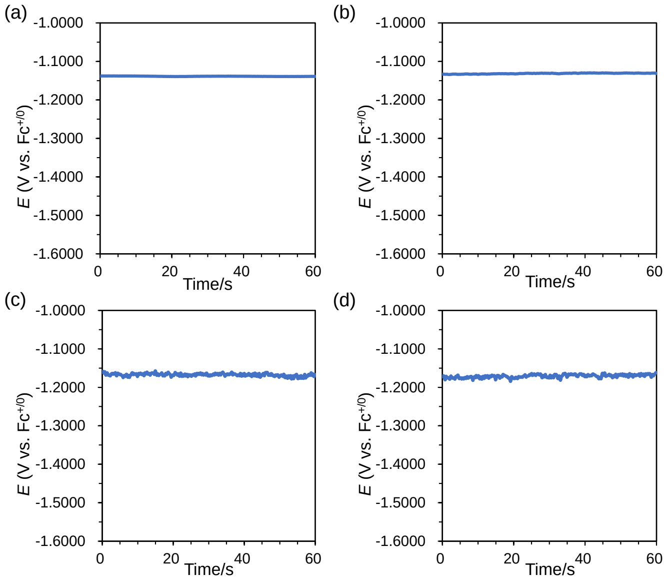

Figure S78. The blue traces show the OCP of E_{H^+/H_2} in DMSO solutions under 1 atm H_2. (a) 1 mM NaOH and H_2O (0.02%, v/v); (b) 5 mM NaOH and H_2O (0.1%, v/v); (c) 10 mM NaOH and H_2O (0.2%, v/v); (d) 25 mM NaOH and H_2O (0.5%, v/v).

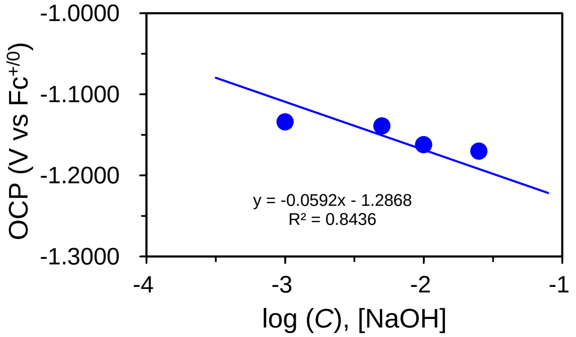

Figure S79. OCP experiment values versus log (*C*), [NaOH] in DMSO.

6e. Estimation of E_{H_2O/O_2} based on OCP measurements of E_{H^+/H_2}

An example for estimating the E_{H_2O/O_2} in MeCN (with 1 mM [DMF-H][OTf]):

The reduction potential for O_2/H_2O in MeCN may be estimated using i) the standard aqueous cell potential for $O_2 + 2\ H_2 \rightarrow 2\ H_2O$, ii) the measurement of the OCP for H^+/H_2 (E_{H^+/H_2}) with different conditions (Table S14, S15, and S16), and iii) the Gibbs free energy to transfer H_2O from H_2O to MeCN (with 1 mM [DMF-H][OTf]) (Table S12). For example, E_{H_2O/O_2} for the MeCN solution in the presence of 1 mM [DMF-H][OTf] is calculated by the following equations:

$$2\ H_2O_{(aq)} \rightleftharpoons O_{2(g)} + 2\ H_{2(g)} \quad \text{(i)}$$

$$[E^{\circ}_{H_2O/O_2(aq)} = 1.23\ \text{V vs}\ E_{H^+/H_2(aq)},\ \Delta G^{\circ}_{H_2O/O_2(aq)} = -\ 113.46\ \text{kcal mol}^{-1}]$$

$$H_{2(g)} \rightleftharpoons 2\ H^+_{(MeCN)} + 2\ e^-_{(V\ vs\ Fc^{+/0})} \quad \text{(ii)}$$

$$[E_{H^+/H_2(1\ mM\ [DMF\text{-}H][OTf]\ in\ MeCN)} = 0.041\ \text{V vs Fc}^{+/0},\ 2 \times \Delta G_{H^+/H_2(1\ mM\ [DMF\text{-}H][OTf]\ in\ MeCN)} = -3.78\ \text{kcal mol}^{-1}]$$

$2\ H_2O_{(MeCN)} \rightleftharpoons 2\ H_2O_{(aq)}$ (iii)

$[2 \times \Delta G_{H_2O(aq \rightarrow 1\ mM\ [DMF\text{-}H][OTf]\ in\ MeCN)} = 5.58\ kcal\ mol^{-1}]$

$2\ H_2O_{(1\ mM\ [DMF\text{-}H][OTf]\ in\ MeCN)} \rightleftharpoons O_{2(g)} + 4\ e^{-}_{(V\ vs\ Fc^{+/0})} + 4\ H^{+}_{(1\ mM\ [DMF\text{-}H][OTf]\ in\ MeCN)}$ (iv)

$\Delta G_{H_2O/O_2(1\ mM\ [DMF\text{-}H][OTf]\ in\ MeCN)} = -111.66\ kcal\ mol^{-1}$, $\boldsymbol{E_{H_2O/O_2(MeCN\ with\ 1\ mM\ [DMF\text{-}H][OTf])} = 1.21\ V\ vs\ Fc^{+/0}}$

The potentials from equations i) and ii) are converted into free energy using the Nernst equation. After summing and balancing the free energy equations, they are converted back into potentials. All the organic solvents listed below are calculated using this method.

Table S16. The oxidation potential for H_2O/O_2 (E_{H_2O/O_2}) with various concentrations of [DMF-H][OTf].

Solvent/ conc. (mM)	1	5	10	50	100	500	1000
IPA[b]	0.61	0.68	0.70	0.73	0.74	0.75	0.76
MeOH	0.69	0.73	0.75	0.80	0.82	0.78	0.84
EtOH[b]	0.67	0.68	0.69	0.74	0.73	0.77	0.78
MeCN	1.21	1.27	1.28	1.31	1.32	1.34	1.35
DMF	0.32	0.36	0.37	0.41	0.43	0.47	0.49
DMA[b]	0.45	0.46	0.47	0.49	0.50	0.55	0.57
DMSO[b]	0.38	0.40	0.44	0.47	0.48	0.51	0.54

[a]All potentials averaged from triplicate measurements and are referenced to $Fc^{+/0}$ in V. [b]Deviated from the Nernstian behavior (see section 6b for the OCP measurements of E_{H^+/H_2})

Table S17. The oxidation potential for H_2O/O_2 (E_{H_2O/O_2}) with various concentrations of [NaOH].

Solvent/ conc. (mM)	1	5	10	25	50	100
THF	1.05	0.93	0.92	0.89	0.88	0.83
Dioxane	0.20	0.18	0.14	0.08	0.04	0.03
Acetone	0.67	0.66	0.63	0.59	0.57	0.55
MeOH	0.00	−0.03	−0.06	−0.08	−0.08	−0.11
EtOH	0.19	0.18	0.16	0.15	0.13	insoluble
MeCN	0.21	0.16	0.13	0.12	0.13	0.13
DMF	−0.16	−0.25	−0.27	−0.27	−0.33	−0.31
DMA	−0.17	−0.21	−0.24	−0.25	−0.27	−0.28
DMSO	0.03	0.03	0.00	0.00	insoluble	

[a]All potentials averaged from triplicate measurements and are referenced to $Fc^{+/0}$ in V.

Table S18. The oxidation potential for H_2O/O_2 (E_{H_2O/O_2})[a] with various concentrations of [NaOH].

Solvent/ conc. (mM)	1	3	5	7	10
IPA	0.08	0.05	0.03	−0.01	−0.01

[a]All potentials averaged from triplicate measurements and are referenced to $Fc^{+/0}$ in V.

7. Compound Spectra

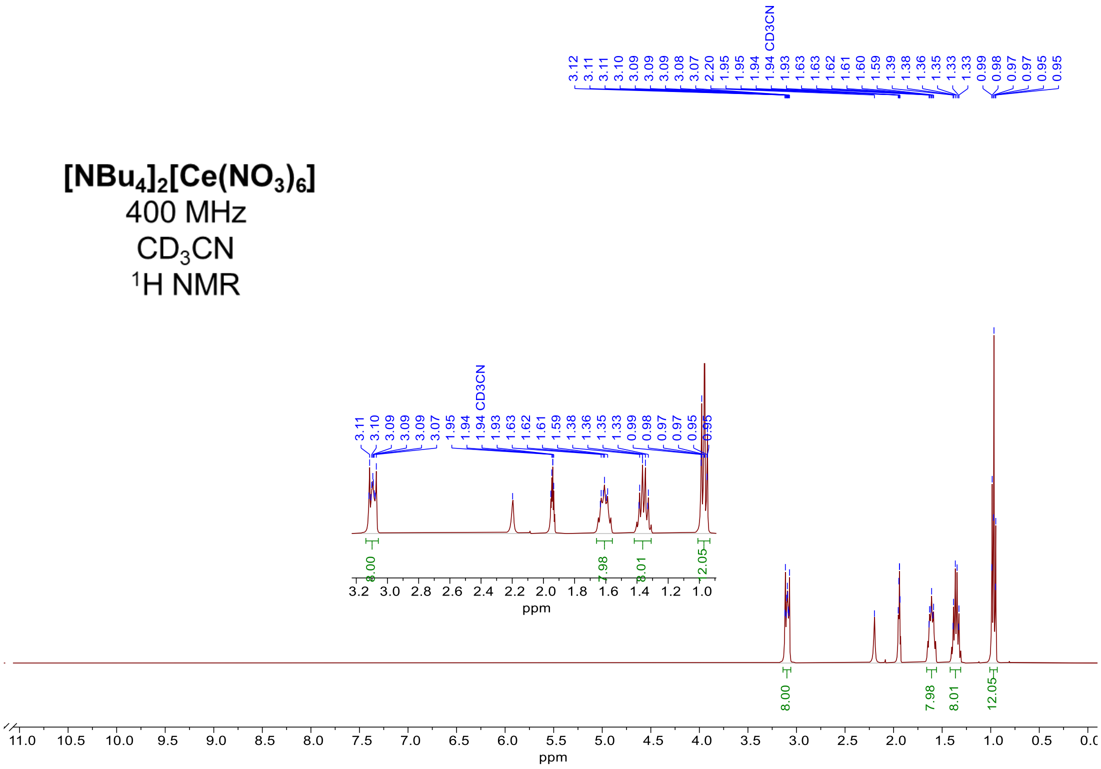

Figure S80. ^{1}H NMR spectrum of **$[NBu_4]_2[Ce(NO_3)_6]$**.

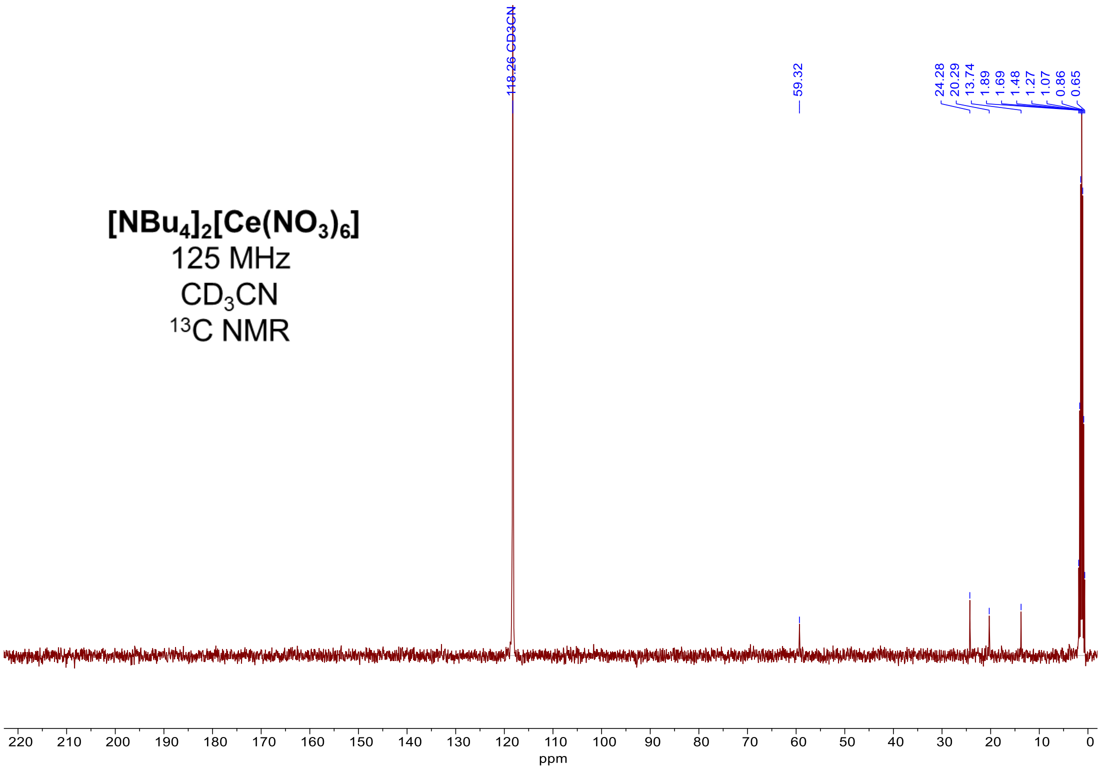

Figure S81. ^{13}C NMR spectrum of **$[NBu_4]_2[Ce(NO_3)_6]$**.

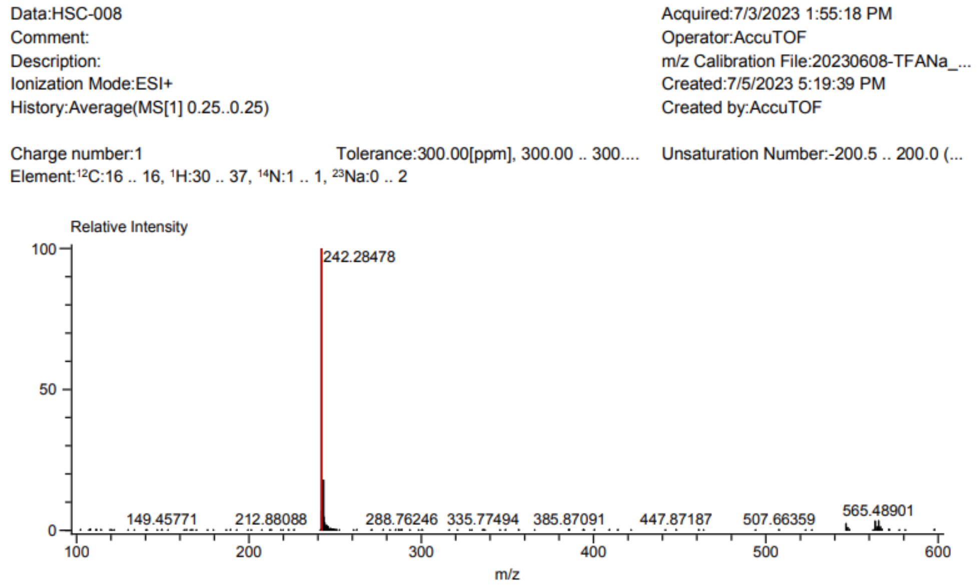

Mass	Intensity	Calc. Mass	Mass Difference [mDa]	Mass Difference [ppm]	Possible Formula
242.28478	342223.15	242.28477	0.00	0.01	$^{12}C_{16}{}^{1}H_{36}{}^{14}N_1$

Figure S82. HR-ESI(+)-MS of **$[NBu_4]_2[Ce(NO_3)_6]$**

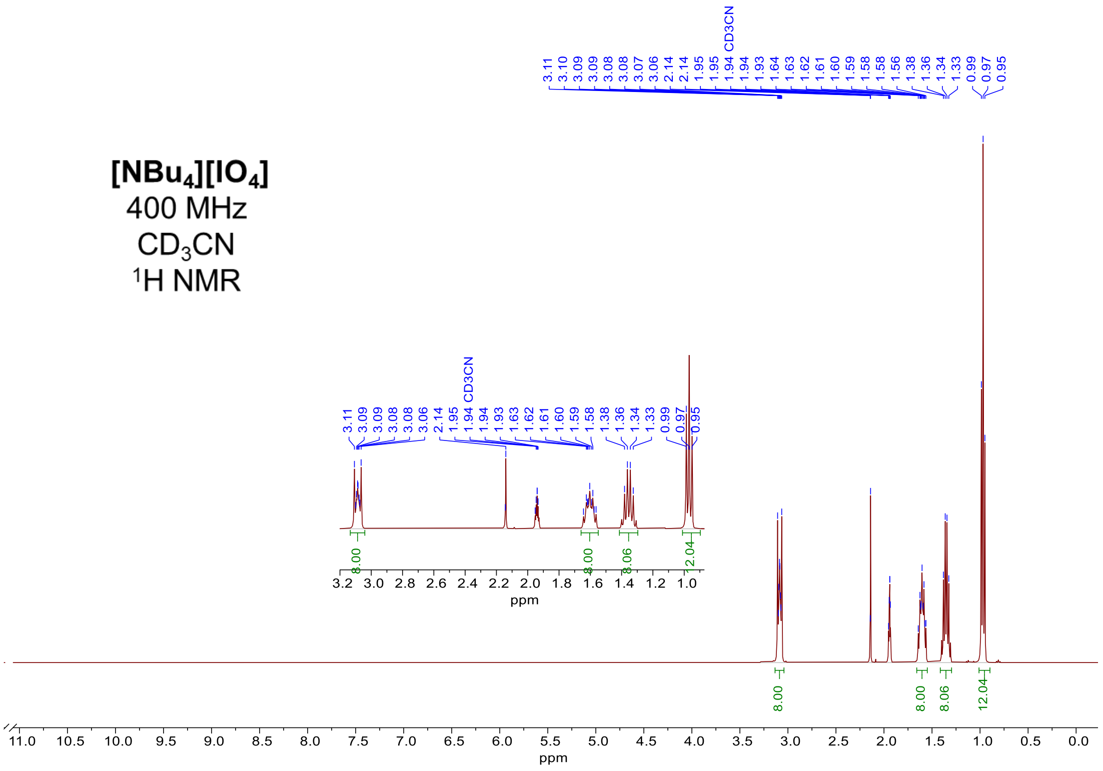

Figure S83. ^{1}H NMR spectrum of **$[NBu_4][IO_4]$**

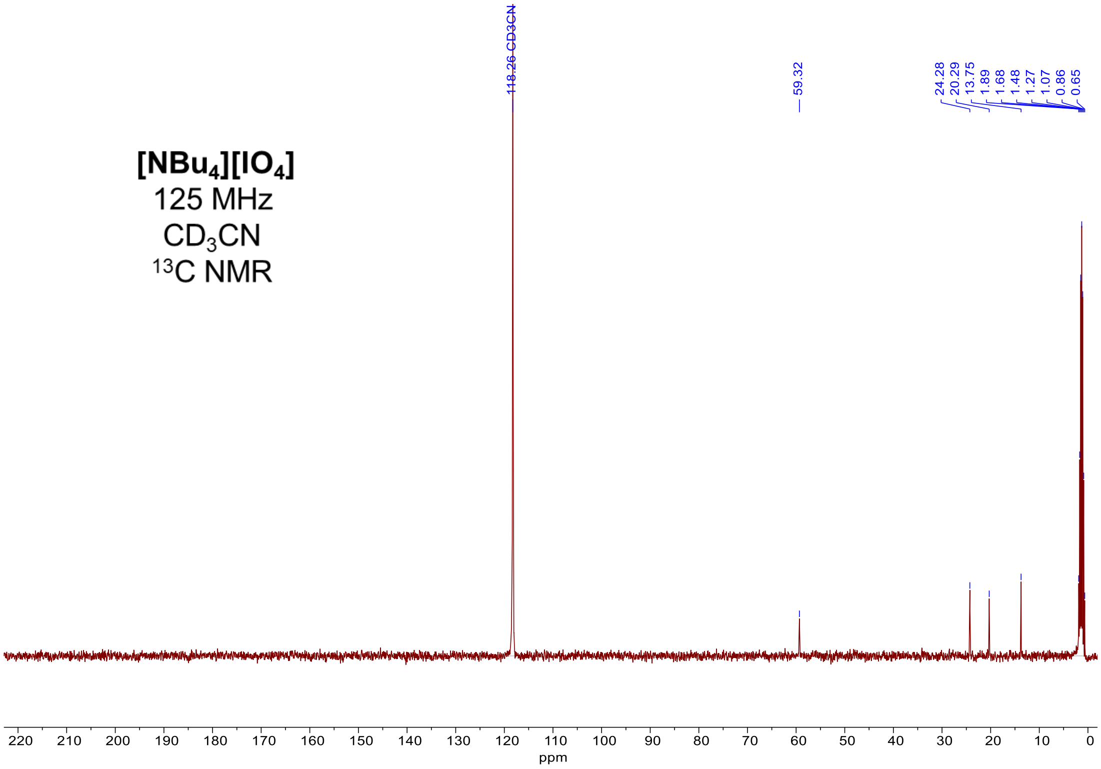

Figure S84. ^{13}C NMR spectrum of **[NBu$_4$][IO$_4$]**

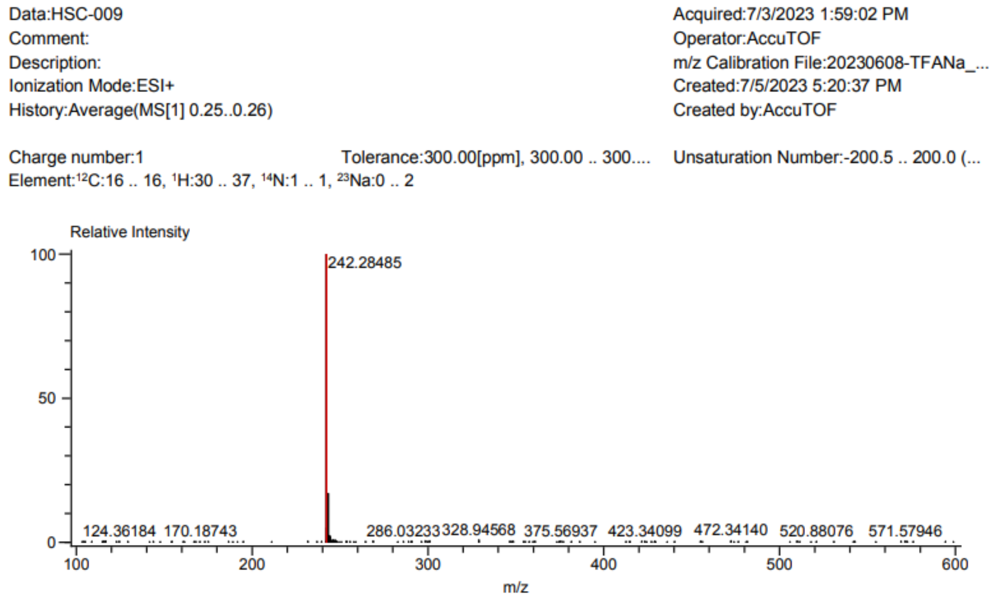

Mass	Intensity	Calc. Mass	Mass Difference [mDa]	Mass Difference [ppm]	Possible Formula
242.28485	243188.00	242.28477	0.07	0.31	$^{12}C_{16}{}^{1}H_{36}{}^{14}N_1$

Figure S85. HR-ESI(+)-MS of **[NBu$_4$][IO$_4$]**

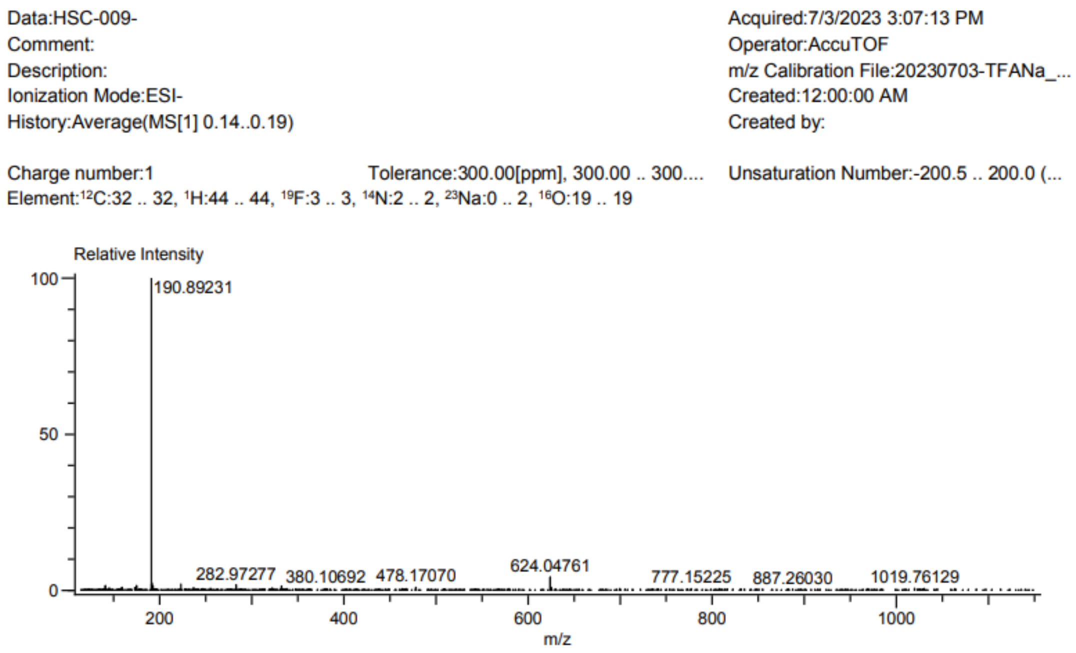

Figure S86. HR-ESI(−)-MS of **[NBu_4][IO_4]**

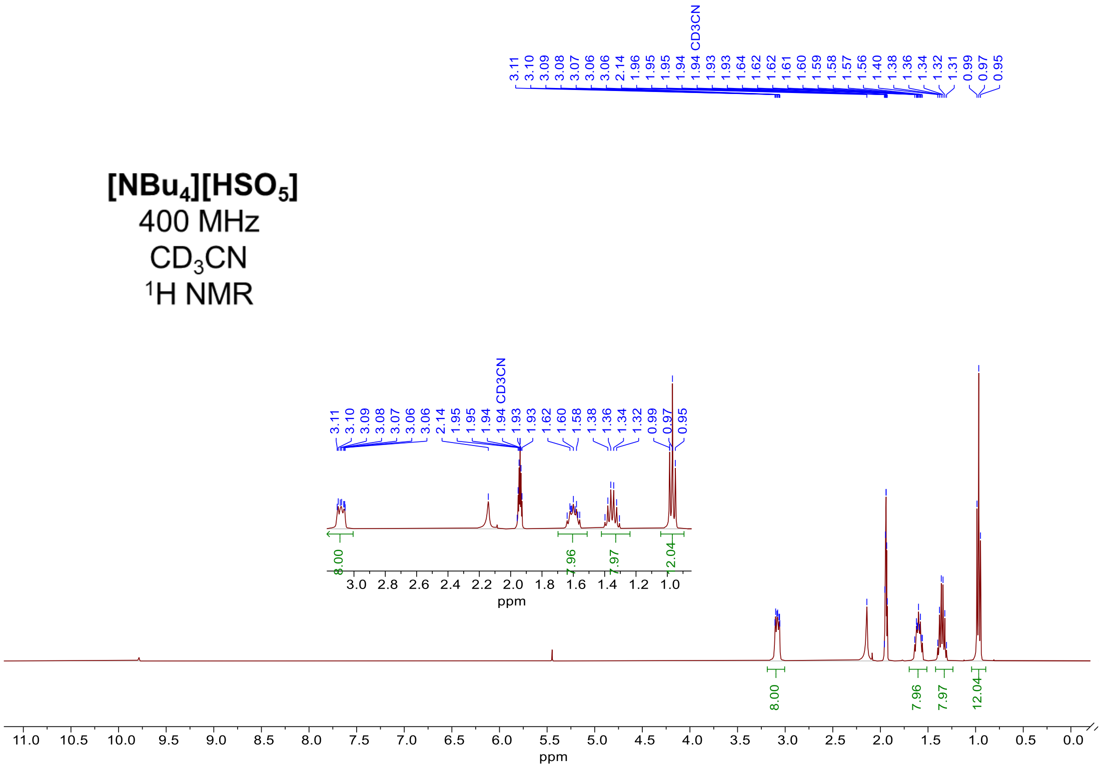

Figure S87. ^{1}H NMR spectrum of **[NBu_4][HSO_5]**.

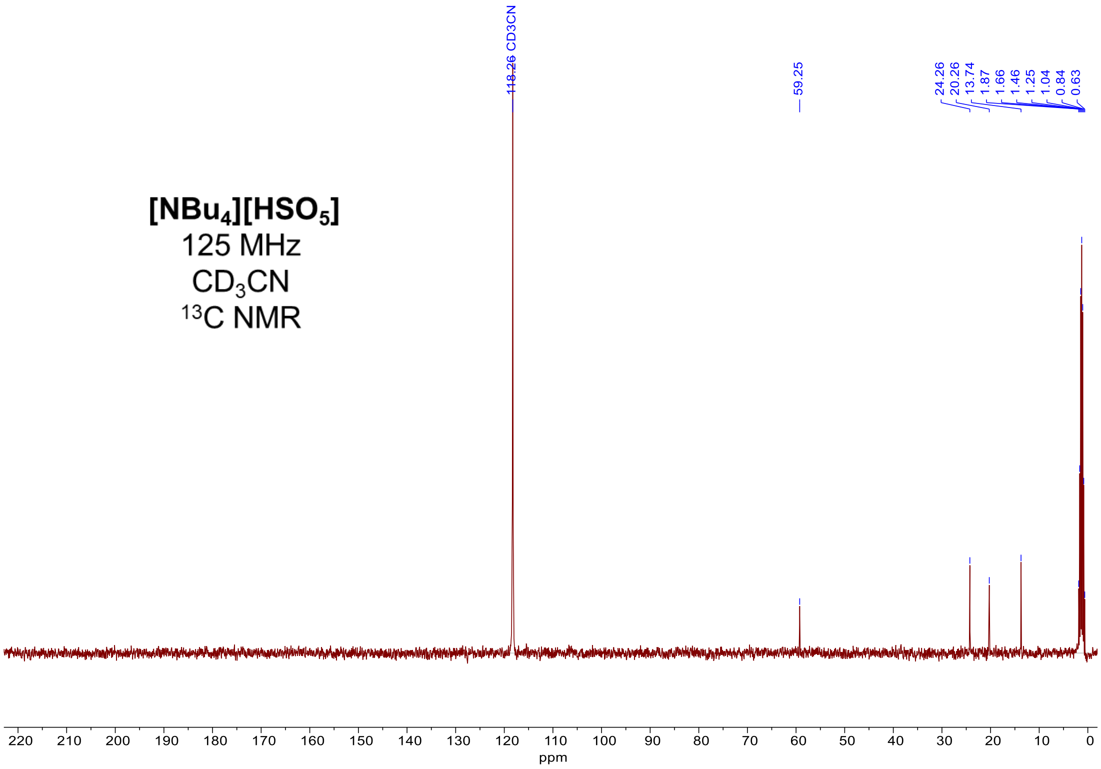

Figure S88. ^{13}C NMR spectrum of **[NBu$_4$][HSO$_5$]**

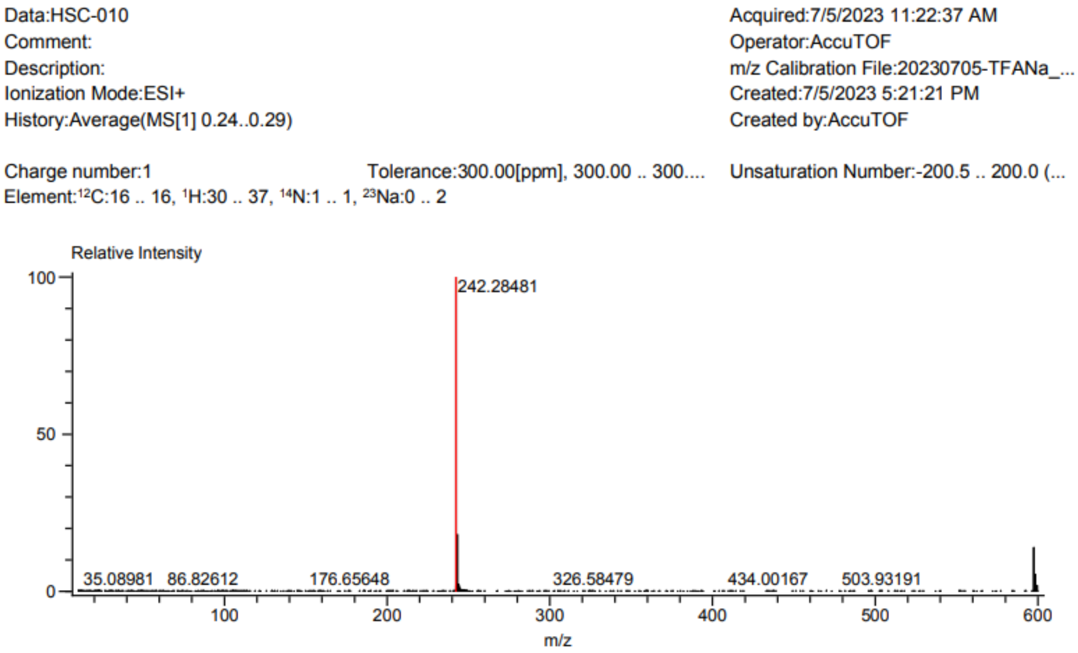

Mass	Intensity	Calc. Mass	Mass Difference [mDa]	Mass Difference [ppm]	Possible Formula
242.28481	208707.79	242.28477	0.03	0.13	$^{12}C_{16}{}^{1}H_{36}{}^{14}N_1$

Figure S89. HR-ESI(+)-MS of **[NBu$_4$][HSO$_5$]**

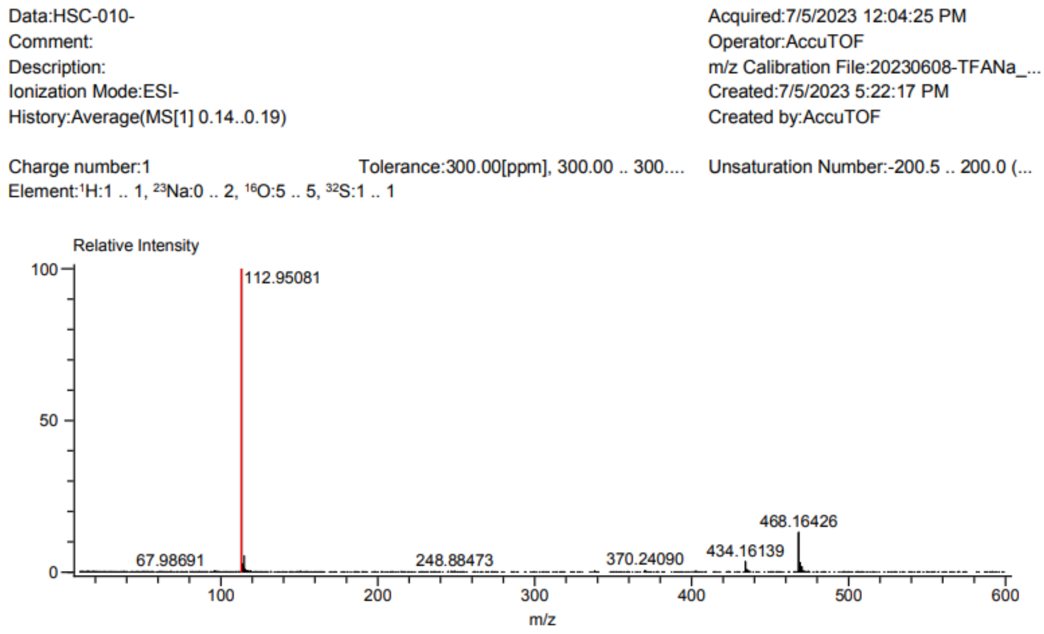

Mass	Intensity	Calc. Mass	Mass Difference [mDa]	Mass Difference [ppm]	Possible Formula
112.95081	727473.77	112.95447	-3.66	-32.41	$^{1}H_{1}{}^{16}O_{5}{}^{32}S_{1}$

Figure S90. HR-ESI(−)-MS of **[NBu$_4$][HSO$_5$]**

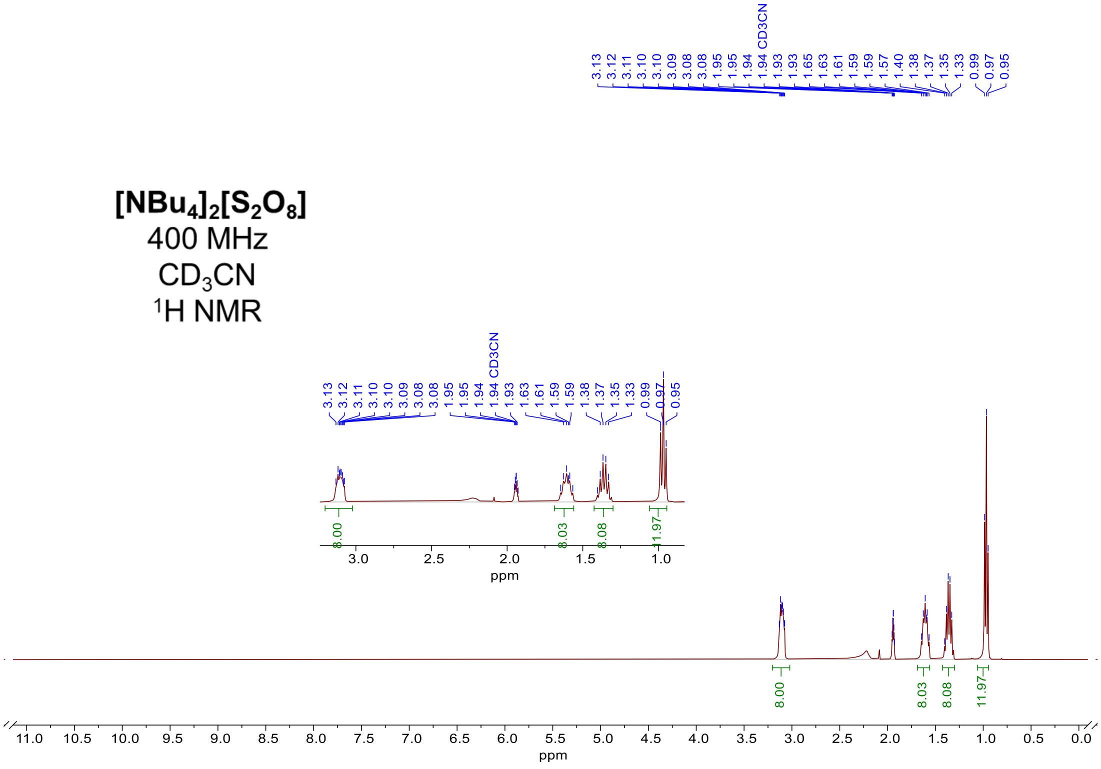

Figure S91. ^{1}H NMR spectrum of **[NBu$_4$]$_2$[S$_2$O$_8$]**

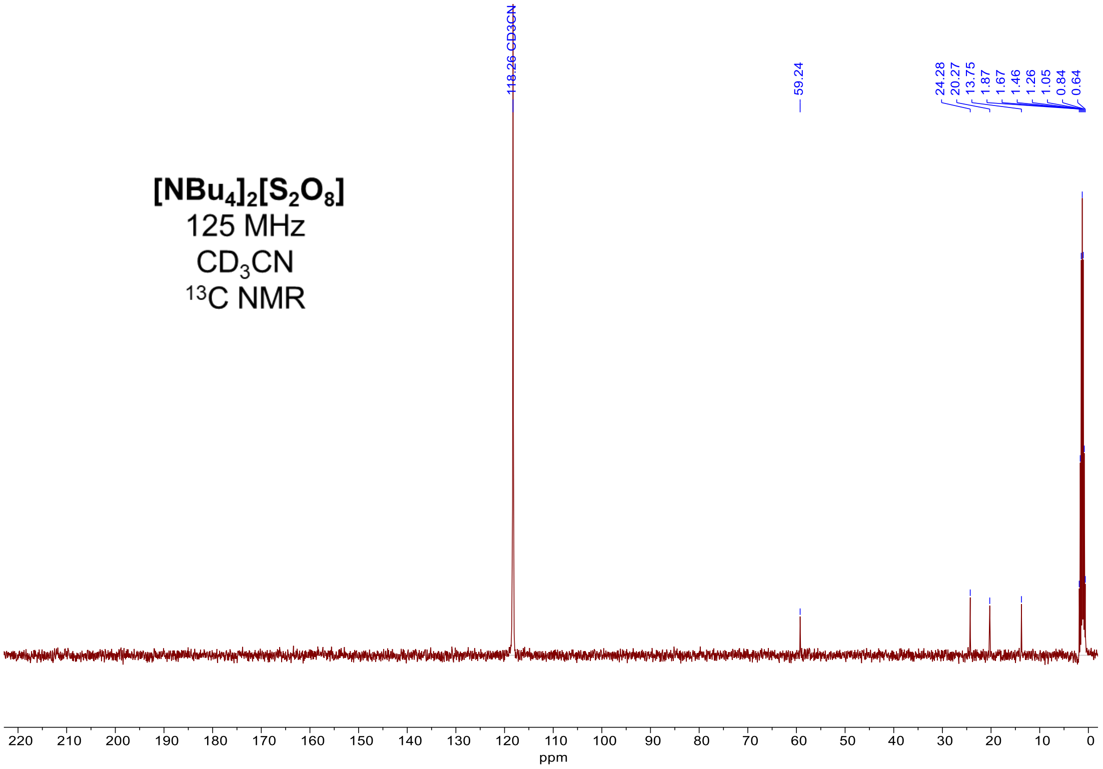

Figure S92. ^{13}C NMR spectrum of **$[NBu_4]_2[S_2O_8]$**

Data:HSC-011
Comment:
Description:
Ionization Mode:ESI+
History:Average(MS[1] 0.26..0.31)

Acquired:7/5/2023 11:26:03 AM
Operator:AccuTOF
m/z Calibration File:20230705-TFANa_...
Created:7/5/2023 5:24:01 PM
Created by:AccuTOF

Charge number:1
Tolerance:300.00[ppm], 300.00 .. 300....
Unsaturation Number:-200.5 .. 200.0 (...
Element:^{12}C:16 .. 16, ^{1}H:36 .. 36, ^{14}N:1 .. 1, ^{23}Na:0 .. 2

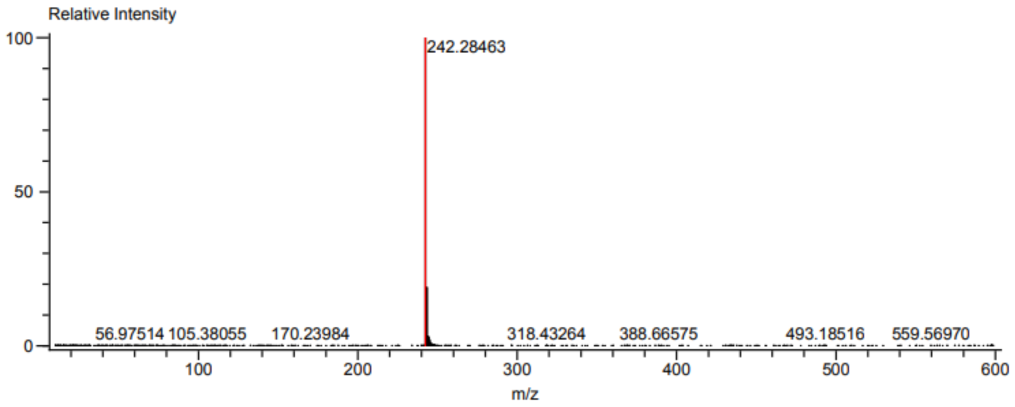

Mass	Intensity	Calc. Mass	Mass Difference [mDa]	Mass Difference [ppm]	Possible Formula
242.28463	277698.13	242.28477	-0.14	-0.59	$^{12}C_{16}{}^{1}H_{36}{}^{14}N_1$

Figure S93. HR-ESI(+)-MS of **$[NBu_4]_2[S_2O_8]$**

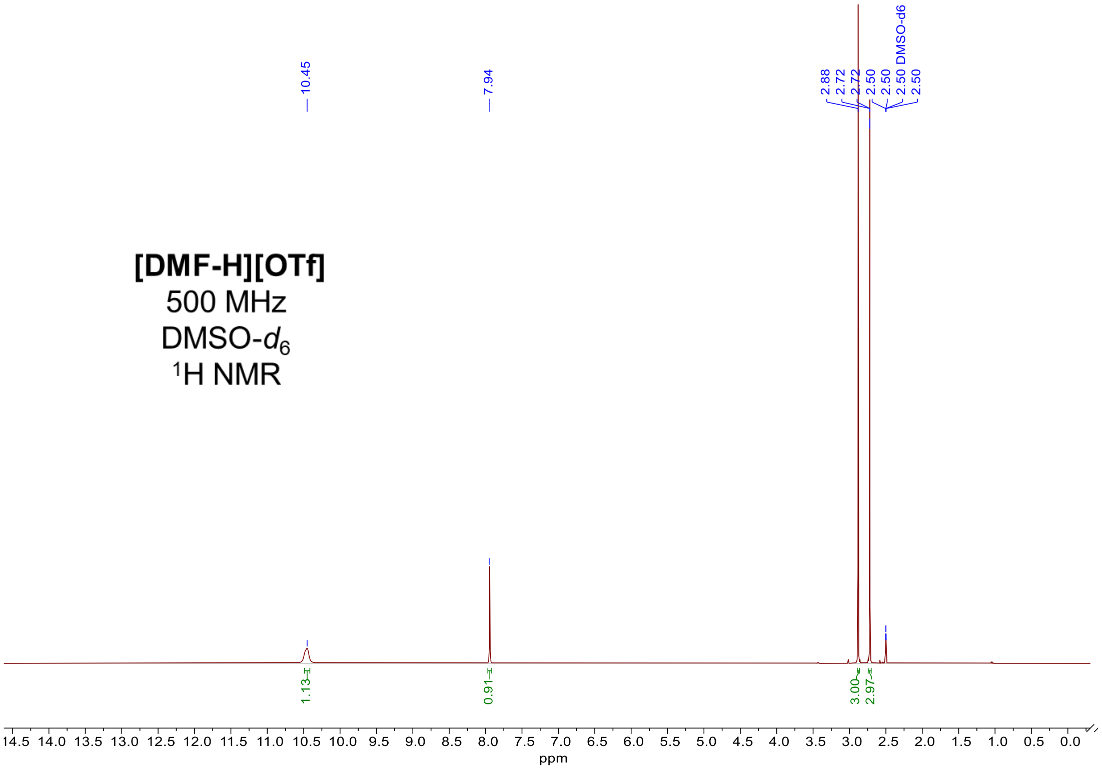

Figure S94.^{1}H NMR spectrum of **[DMF-H][OTf]**

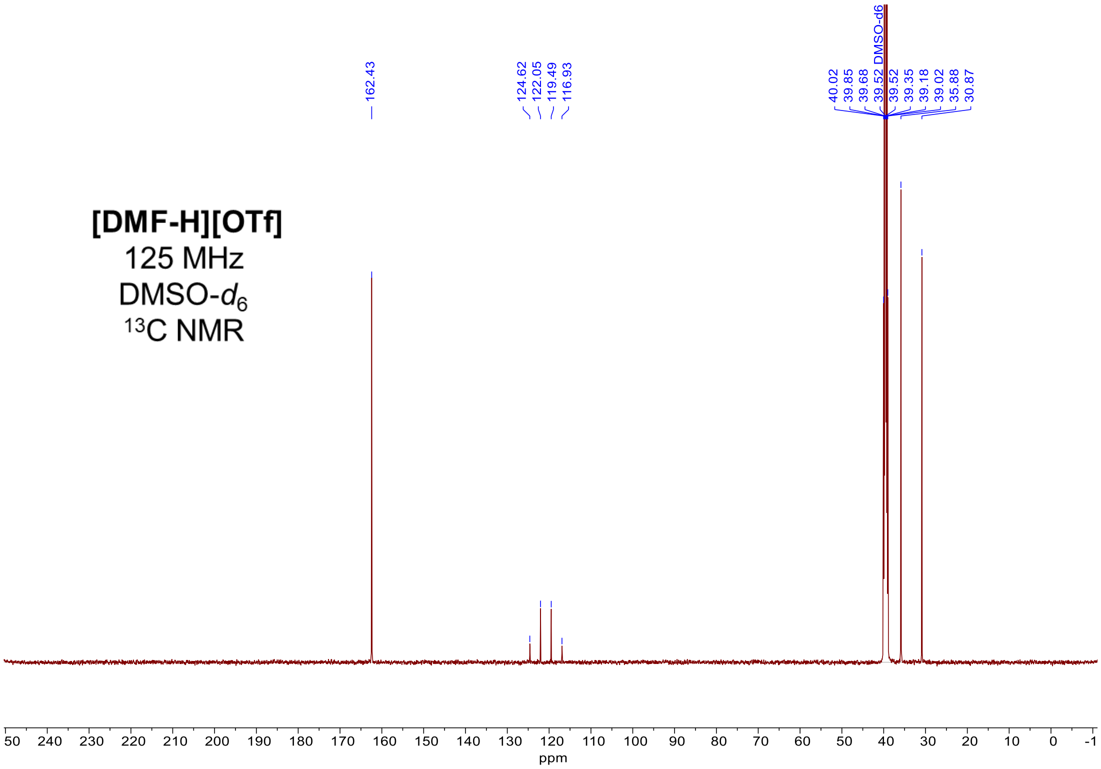

Figure S95.^{13}C NMR spectrum of **[DMF-H][OTf]**

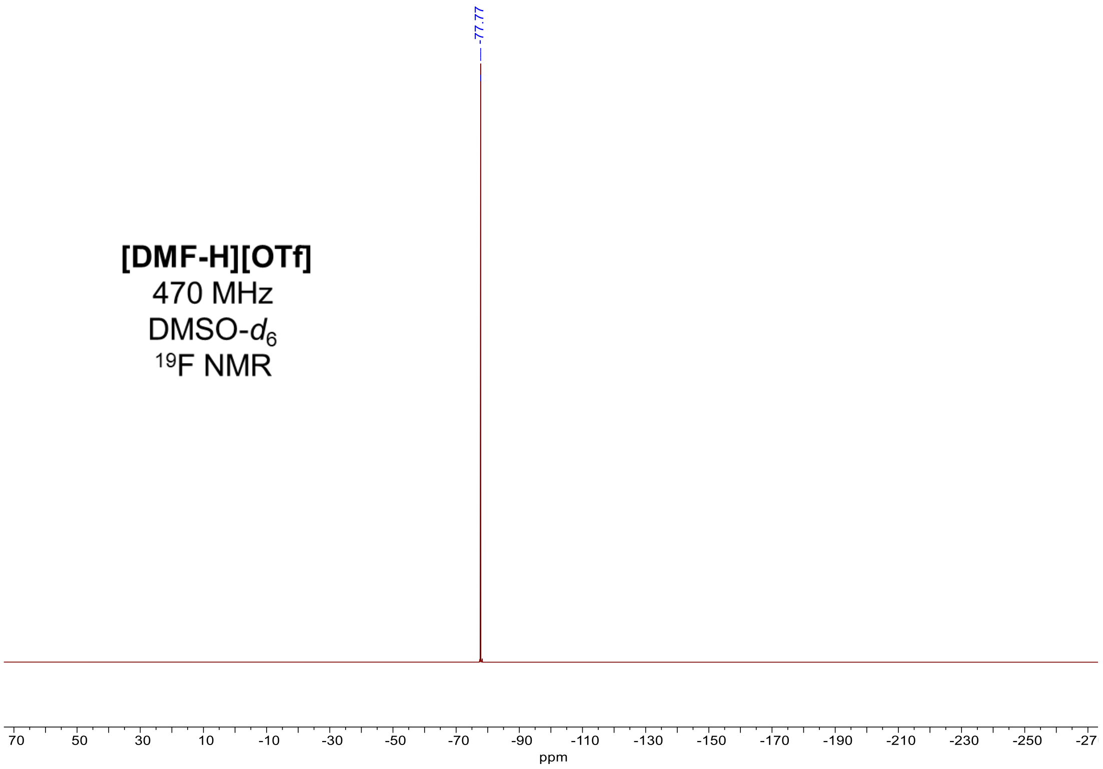

Figure S96. ^{19}F NMR spectrum of **[DMF-H][OTf]**

References

(1) Mishra, A.; Tasiopoulos, A. J.; Wernsdorfer, W.; Abboud, K. A.; Christou, G. High-Nuclearity Ce/Mn and Th/Mn Cluster Chemistry: Preparation of Complexes with $[Ce_4Mn_{10}O_{10}(OMe)_6]^{18+}$ and $[Th_6Mn_{10}O_{22}(OH)_2]^{18+}$ Cores. *Inorg. Chem.* **2007**, *46*, 3105-3115

(2) Hsu, W.-C.; Zeng, W.-Q.; Lu, I. C.; Yang, T.; Wang, Y.-H. Dinuclear Cobalt Complexes for Homogeneous Water Oxidation: Tuning Rate and Overpotential through the Non-Innocent Ligand. *ChemSusChem* **2022**, *15*, e202201317

(3) Travis, Benjamin R.; Ciaramitaro, Benjamin P.; Borhan, B. Preparation of Purified $KHSO_5 \cdot H_2O$ and nBu_4NHSO_5 from Oxone by Simple and Efficient Methods. *Eur. J. Org. Chem.* **2002**, *2002*, 3429-3434

(4) Yang, S. G.; Hwang, J. P.; Park, M. Y.; Lee, K.; Kim, Y. H. Highly efficient epoxidation of electron-deficient olefins with tetrabutylammonium peroxydisulfate. *Tetrahedron* **2007**, *63*, 5184-5188

(5) Favier, I.; Duñach, E. New protic salts of aprotic polar solvents. *Tetrahedron Lett.* **2004**, *45*, 3393-3395

(6) Connelly, N. G.; Geiger, W. E. Chemical Redox Agents for Organometallic Chemistry. *Chem. Rev.* **1996**, *96*, 877-910

(7) Pavlishchuk, V. V.; Addison, A. W. Conversion constants for redox potentials measured versus different reference electrodes in acetonitrile solutions at 25°C. *Inorg. Chim. Acta* **2000**, *298*, 97-102

(8) Roberts, J. A. S.; Bullock, R. M. Direct Determination of Equilibrium Potentials for Hydrogen Oxidation/Production by Open Circuit Potential Measurements in Acetonitrile. *Inorg. Chem.* **2013**, *52*, 3823-3835

(9) Marenich, A. V.; Cramer, C. J.; Truhlar, D. G. Universal Solvation Model Based on Solute Electron Density and on a Continuum Model of the Solvent Defined by the Bulk Dielectric Constant and Atomic Surface Tensions. *J. Phys. Chem. B.* **2009**, *113*, 6378-6396

(10) Haynes, W. M. *CRC handbook of chemistry and physics*; CRC press, 2016.

(11) Vogel, I. *Practical organic chemistry*; Citeseer, 1974.

(12) Marenich, A. V.; Cramer, C. J.; Truhlar, D. G. Generalized Born Solvation Model SM12. *J. Chem. Theory Comput.* **2013**, *9*, 609-620

(13) Kütt, A.; Tshepelevitsh, S.; Saame, J.; Lõkov, M.; Kaljurand, I.; Selberg, S.; Leito, I. Strengths of Acids in Acetonitrile. *Eur. J. Org. Chem.* **2021**, *2021*, 1407-1419

(14) Fourmond, V.; Jacques, P.-A.; Fontecave, M.; Artero, V. H_2 Evolution and Molecular Electrocatalysts: Determination of Overpotentials and Effect of Homoconjugation. *Inorg. Chem.* **2010**, *49*, 10338-10347

(15) Davis, M. M. *Acid-base behavior in aprotic organic solvents*; US National Bureau of Standards, 1968.
